# Ensemble completeness in conformer sampling: the case of small macrocycles

**DOI:** 10.1186/s13321-021-00524-0

**Published:** 2021-07-29

**Authors:** Lea Seep, Anne Bonin, Katharina Meier, Holger Diedam, Andreas H. Göller

**Affiliations:** 1 Pharmaceuticals R&D, Digital Technologies, Bayer AG, 42096 Wuppertal, Germany; 2 Engineering & Technology, Applied Mathematics, Bayer AG, 51368 Leverkusen, Germany

**Keywords:** Conformer sampling, Molecular dynamics, Conformer ensemble

## Abstract

In this study we compare the three algorithms for the generation of conformer ensembles Biovia BEST, Schrödinger Prime macrocycle sampling (PMM) and Conformator (CONF) form the University of Hamburg, with ensembles derived for exhaustive molecular dynamics simulations applied to a dataset of 7 small macrocycles in two charge states and three solvents. Ensemble completeness is a prerequisite to allow for the selection of relevant diverse conformers for many applications in computational chemistry. We apply conformation maps using principal component analysis based on ring torsions. Our major finding critical for all applications of conformer ensembles in any computational study is that maps derived from MD with explicit solvent are significantly distinct between macrocycles, charge states and solvents, whereas the maps for post-optimized conformers using implicit solvent models from all generator algorithms are very similar independent of the solvent. We apply three metrics for the quantification of the relative covered ensemble space, namely cluster overlap, variance statistics, and a novel metric, Mahalanobis distance, showing that post-optimized MD ensembles cover a significantly larger conformational space than the generator ensembles, with the ranking PMM > BEST >> CONF. Furthermore, we find that the distributions of 3D polar surface areas are very similar for all macrocycles independent of charge state and solvent, except for the smaller and more strained compound 7, and that there is also no obvious correlation between 3D PSA and intramolecular hydrogen bond count distributions.

**Supplementary Information:**

The online version contains supplementary material available at 10.1186/s13321-021-00524-0.

## Introduction

Molecules at ambient conditions are flexible fluctuating three-dimensional objects composed of atoms held together by electrons. Since there exists no appropriate and applicable description for this state, computational chemists apply different types of approximations. For tasks like QSAR/machine learning predictions, structure alignments, pharmacophores, docking, or molecular energy calculations (e.g. binding energies, relative configurational energies, conformer or reaction energies) molecular descriptors, structural fingerprints, line notations, classical molecular mechanics or quantum mechanics are applied. Most of these tasks to be performed need at least one set of 3D coordinates, and many approaches like pharmacophore searching or docking rely either on a coordinate set of the binding or minimum energy conformation or an ensemble of low-energy conformations, respectively. Only if one can create all relevant conformations or otherwise relevant representatives from the complete accessible phase space one can be sure not to introduce random errors into model setup. It is important to emphasize that the ensemble space is not only about energy minima: deep minima can correspond to small populations while shallow minima can be more populated. Conformations in binding pockets are influenced by the pocket and may not correspond to minima in solvent. The ability of a compound to pre-adapt in medium A to a conformer state relevant in medium B (A and B being solvents, membranes, binding pockets, for instance) has recently be shown again [1] but is a long-known fact [2]. Adequate conformational sampling should therefore be based on free energy (including entropic effects) rather than on the potential energy only. We are interested in relevant statistical information (most populated states).

Structure-based machine learning is typically based on descriptors from atomic connectivity or 2D structures. Nevertheless, with comparative field analysis (CoMFA) already in 1988 the first 3D-based method appeared [3]. The concept was further refined (without finding broad application) with the 4-dimensional xMap [4] approach that avoids the two main issues of CoMFa, namely reliance on only one conformer and the necessity to align the ligands onto each other, and the 5D-QSAR method [5] by Vedani and Dobler that in addition also considers different protonation states. The relevance of 3D conformation-based machine learning recently sees a revival triggered by so-called beyond rule of five compounds [6] and the observation that many ADMET properties of compounds rely on conformational flexibility determined inter alia by intramolecular hydrogen bonding [7]. One such descriptor derived for modeling solvation free energies is the MDFP by Riniker [8] which allows to transfer information from a molecular dynamics simulation in one solvent to another solvent and to derive distribution coefficients. Clever descriptions of three-dimensional features of molecules will certainly constitute one approach towards the improvement of in silico ADMET and other ML models.

Unfortunately, there is no experimental technique that consistently provides information on the accessible ensemble, especially since the surrounding medium strongly influences conformational preference. There is data for any aggregate phase and medium but experiments in different phases or media will give different results, for well-understood reasons. Gas-phase conformer coordinates describe the structure in a more or less undisturbed state but are limited to small structures able to sublimate into gas-phase without decomposition. In solvent (liquid phase for most organic molecules is not accessible) the conformer ensembles can only indirectly be determined by shifts and couplings from spectroscopic methods like NMR [9] or IR- or FTIR- spectroscopy [10, 11], often in combination with mass spectroscopy to fragment larger molecules and with quantum-chemical calculations [12]. Solid state coordinates are obtained by crystallography either for the ligand itself or for a ligand co-crystallized with a target protein. Small molecule crystals provide high-resolution coordinates which however often do not represent the global minimum conformation, as they are defined by intra—and more importantly—intermolecular interactions like hydrogen bonds, pi-stacking, dispersion, charge-charge interactions etc., which strongly influence the torsional angles in particular. Coordinates derived from protein–ligand complexes are also heavily biased by intermolecular interactions and additionally are significantly less accurate, providing only heavy-atom positions which often have non-equilibrium distances, angles and torsions [13], and even high-resolution structures often have no electron density for parts of the ligand [14]. A study by Perola [2] reported that from the 150 protein–ligand complexes evaluated, about 60% were no local minima, about 60% had strain energies of up to 5 kcal/mol and at least 10% had strain energies higher than 9 kcal/mol. Other studies, using higher levels of theory, report much lower (< 2 kcal/mol) or much higher (> 10 kcal/mol) strain energies, as summarized by Hawkins [15].

A computational process for the generation of conformers must fulfill two requirements, namely create a complete ensemble of energetically accessible conformers to allow for a selection of a representative subset and provide accurate conformer energies as a prerequisite to select the subset. In a previous publication we have benchmarked [16] two force-fields, three semi-emprical and a performance-optimized density functional method with regard to accurate relative energies. In this publication we look at the completeness of conformer ensembles from three different algorithms for conformer generation in comparison with ensembles derived from extensive molecular dynamics simulations applying multiple starting conformers in three solvents and two different charge states. The intention of our study is to identify the generator algorithm best suited to the task, since in industry we are willing to accept fast and approximate methods as long as they are reliable or at least allow to identify the breakdown of the approximation. To our knowledge there is one study by Agrafiotis et al. addressing explicitly the topic of ensemble completeness [17] and one study by Schrödinger that followed the same concept but with some limitations regarding the completeness of the MD derived ensemble [18]. Additionally to cluster-based and covariance metrics approaches to identify the conformer overlaps between MD and generators, we propose a novel measure for the quantification of overlap of ensembles of different origin but also discuss the “uncertainty principle” for measuring ensemble completeness.

In this article we focus on seven small macrocycles from a series of about 50 compounds we had synthesized in order to investigate parameters that determine cell permeation [19], influenced by the work of the groups of Jacobsen and Lokey [20–23]. In a future study we plan to extend this work to typical drug-like small molecules.

## Methods

### Dataset

**Scheme 1:**
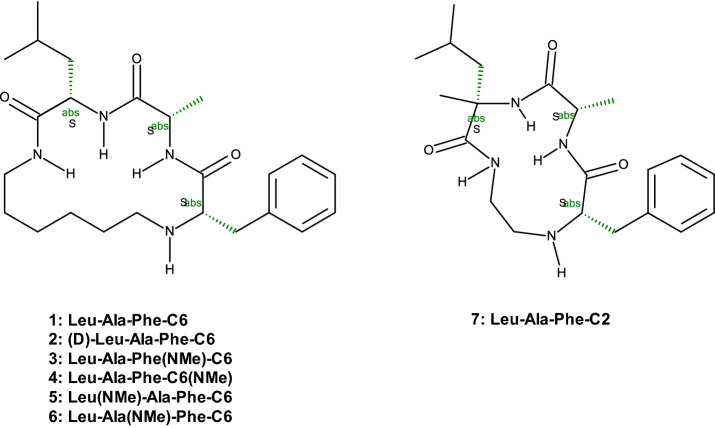
Structures of the macrocycles

One 12-membered and six 16-membered macrocycles from our recent [16] publication were selected as test cases for this work. They are representatives for the broad range of membrane permeabilities in the respective compound class. Compounds are shown in Scheme 1 and information on amino acid sequence, numbers of conformers generated, and physicochemical properties is provided in Table 1. All simulations were performed for the compounds in their neutral and protonated forms (except for N-methylated **3)**. pK_a_ values were calculated at pH 7.4 (pH in blood plasma) by the pK_a_ module [24] of the software ADMET PREDICTOR 7.1 by Simulations Plus [25]. Lipophilicity as expressed by the logD at buffer pH 7.5 was calculated by an in-house machine-learning model based on about 80,000 experimental values [26], whereas TPSA reflects the topological polar surface area according to Ertl et al. [27] Properties of the 16-membered macrocycles show low variance.

**Table 1:** Property space of the dataset, listing amino acid sequence, calculated physicochemical properties pK_a_, logD, molecular weight in g/mol and topological polar surface area TPSA in Å^2^, and numbers of conformers generated by conformer generators

cpd	sequence	pKa	Molec. weight	logD	TPSA	# conf BEST	# conf PMM	# conf CONF
1	Leu-Ala-Phe-C6	7.44	444.61	2.36	99.32	198	187	167
2	(D)-Leu-Ala-Phe-C6	7.44	444.61	2.36	99.32	198	197	162
3	Leu-Ala-Phe(NMe)-C6	6.70	458.64	2.68	90.54	199	198	155
4	Leu-Ala-Phe-C6(NMe)	7.24	458.64	2.50	90.54	200	197	163
5	Leu(NMe)-Ala-Phe-C6	7.33	458.64	2.43	90.54	200	200	136
6	Leu-Ala(NMe)-Phe-C6	7.19	458.64	2.27	90.54	200	195	196
7	Leu-Ala-Phe-C2	6.39	388.50	1.89	99.33	200	60	181

### Conformer generation

Starting 3D coordinates in SD file format were generated by CORINA [28] version 4.2.0 0026 using the driver option “write hydrogens”. For all calculations using the Schrödinger Suite we applied version 2018–4 [29].

Three sets of conformers were tested. The first set was created using the BEST algorithm [30, 31] implemented in BIOVIA Pipeline Pilot [32] generating up to 200 diverse conformers with an energy window of 50 kcal mol^−1^. In contrast to our previous work [1], here, we were concerned with thorough sampling instead of speed. The second set (abbreviated PMM) was created with Schrödinger PRIME MACROCYCLE SAMPLING with 200 requested conformers and “sample peptide bonds” and “preserve major ring shape” deselected.

The third set (abbreviated CONF) was obtained with the software CONFORMATOR [33] with 200 conformers requested, “quality” set to “best” and keeping “macrocycle_size” at 10 atoms. No other flags were used.

Each conformer set was then post-optimized by macromodel using the OPLS3e [34] forcefield with extended cut-off and FF charges, using default settings, i.e. conjugate gradient PRCG optimizer with maximum 2500 iterations, gradient convergence threshold of 0.05 kJ mol^−1^ Å^−1^ and no constraints applied.

In an alternate setting we assessed the space of non-optimized conformations obtainable from generators for the example of compound **1** in its neutral state in comparison with the non-optimized MD snapshots. For this we had to stretch the settings of the algorithms considerably, still never reaching the numbers obtained from MD. The settings applied that differ from the ones before were “-n 30,000” for conformator, “discard existing conformations = false”, “required = 30,000”, “energy threshold = 10,000 kJ mol^−1^″, separate conformer = false”, “minimization = false” for BEST, and 100,000 conformations requested in case of PMM, respectively.

### Molecular dynamics simulations

MD simulations were carried out with DESMOND [35, 36] as implemented in the Schrödinger suite in three different solvents, namely SPC water, DMSO and CHCl_3_. Since there is no pre-built CHCl_3_ solvent box, we had to create it following the procedure outlined in the Schrödinger knowledge base [37]. For this we did a 100 ns simulation at 300 K using an NPT ensemble and checked for pressure and temperature fluctuations using the simulation event analysis [38]. Additionally, we also checked for the correct macroscopic density of the solvent.

The System Builder was used to setup systems for the three solvents SPC water, DMSO and custom-created CHCl_3_ using an orthorhombic box shape, the buffer box size set to 10 Å in each direction. We used the OPLS3e forcefield without calculation of custom parameters. For charged ligands the systems were neutralized by adding a chlorine ion.

All standard simulations ran for 100 ns under NPT conditions at 300 K and 1.01325 bar and generating 10,000 snapshots, starting from five diverse input conformers, and in case of molecule **5** (neutral, solvent water) for 3 additional diverse low-energy conformers selected from the PMM ensemble. The relaxation protocol provided in the Schrödinger suite was used, with all advanced options set to defaults. Some simulations were performed at temperatures of 400 K, 500 K, and 800 K as well as one long-running job with 1000 ns. Diverse starting conformers were selected from the BEST conformer ensemble with the Schrödinger tool “Conformer Cluster” based on ring heavy atom root mean square error (RMSE) with “average linkage” and “retain mirror-image conformers” checked, yielding the centroid structure per cluster, and requesting 200 clusters.

Additionally, we did simulated annealing (SA) runs applying a custom 82 step protocol consisting of 10 heating cycles from 300 to 500 K, each cycle having a 10 ns sampling phase at 300 K and 8 ns heating phase. Simulation time was accordingly set to 172 ns resulting in 17,200 snapshots. Again, an NPT ensemble with 1.01325 bar and the upfront relaxation protocol was used. Due to differences in the DESMOND implementations for CPUs and GPGPUs, the thermostat had to be changed to Nose–Hoover [39] for calculations on GPGPUs. For one case study, we performed 5 simulated annealing runs with 5 diverse starting structures.

### Processing of dihedral angles

**Scheme 2:**
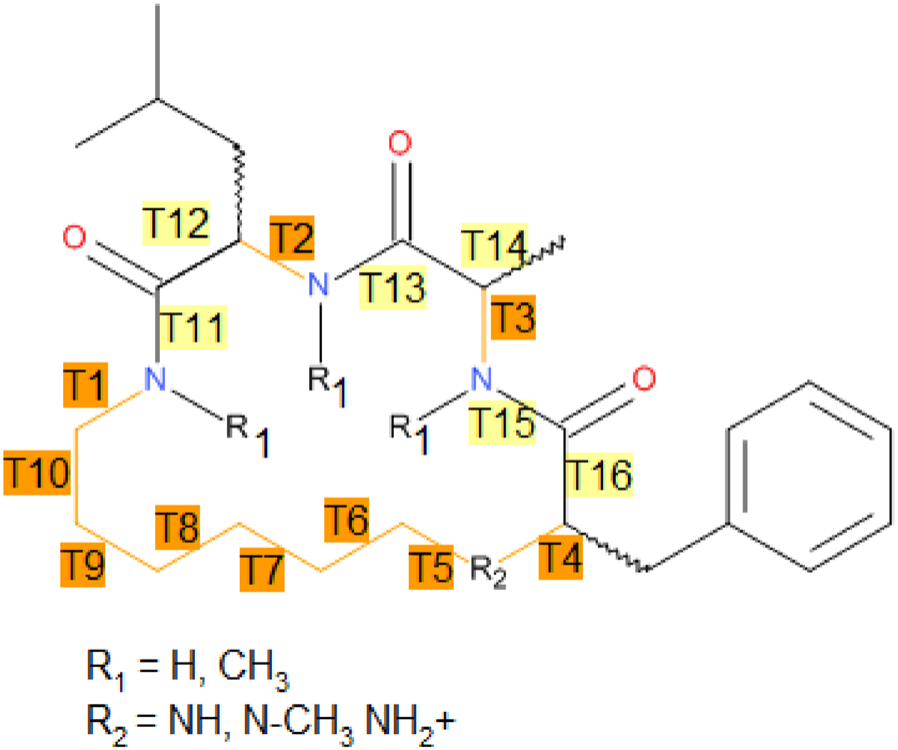
Numbering of torsions; torsions T7, T8, T9, T10 are missing for structure 7

Comparison of ensemble diversity in this publication is based on the ring dihedral angles of the macrocycles. The dihedral angles were numbered in standardized manner (see Scheme 2), leaving torsions T7, T8, T9, T10 undefined in molecule **7** with 12-membered ring.

For the MD and SA snapshots the values were extracted by the Schrödinger script analyze_simulation.py [40]. The configuration files (file type “st2”) with the dihedral definitions were created with the “Simulation Event Analysis panel” for each charge state of each macrocycle (MC).

The BEST, PMM and CONF conformers dihedral angles were calculated with the script “measure_by_numbers.py” provided by Schrödinger support and exported to csv format. All further processing was performed in R [41].

### Torsion space maps

Principal components analysis (PCA) as implemented in R base was used to create a 2-dimensional representation of the multi-dimensional space defined by the ring torsions of macrocycles **1** to **6**. Due to four missing torsions for **7** we projected the torsional space of this ligand onto the map spanned by the 16 torsions from all snapshots from the MD and SA runs performed for the other ligands. It is not possible to use the torsions themselves due to a discontinuity in their definition, i.e., dihedrals of −178° and 178° result in almost perfect superposition of two conformers (minimal atom RMSDs), but in a large distance in latent space. Therefore, we transformed the dihedral angles θ into a pair of values, namely sin(θ) and cos(θ). By this, for instance, − 178° and 178° transform into (0.035; − 0.999) and (− 0.035; − 0.999) populating the same region of latent space.

The PCA mappings were plotted using ggplot and RColorBrewer_1.1.2 [42]. A graphical user plot generation interface allowing for fast and easy comparison of the maps was implemented in Shiny [43], a web application framework for R.

### Properties for color-coding of PCA plots

For each snapshot, we counted the number of intramolecular hydrogen bonds (IMHB). We used values between 110° and 220° as angle and between 1.5 and 2.6 Å as distance thresholds, slightly softening the standard parameters often used, to account for the MD snapshots not being local minimum coordinates. A detailed analysis of prevalence of the different optional IMHB for compounds **1**, **2**, **3** was provided in a previous publication [3] and is out of scope of this work.

Relative energies were derived for each macrocycle with the OPLS3e force field after stripping off the explicit solvent molecules. Since the MD snapshots are not local energy minima but carry a certain but unknown portion of the overall system energy, the values cannot be compared even for the same ligand between the post-optimized conformers and the snapshots. We always set the values for the lowest energy snapshot for each run to zero, knowing that still the maps are only qualitatively comparable.

For comparison to the BEST, PMM and CONF conformers, we post-optimized the snapshots with the OPLS3e forcefield using implicit solvation, resulting in relative energies on the same scale as the ones from the generated and post-optimized conformers.

For the quantification of the overlapping and unique conformations from MD simulations and generator methods we clustered each combined set of minimized structures from MD and the respective generator based on the first eight principal components which have a cumulative explained variance of 67.5%.

The clustering was conducted using the function “kmeans” from the internal R-package 'stats' [44]. K-means identifies a pre-specified number of clusters that minimizes the within-cluster sum of squares. This is done by randomly picking cluster centers and assigning each point to the closest cluster (evaluated by Euclidean distance), re-calculating the new center of the cluster and assigning again each data point to its closest cluster. This is iterated till no cluster changes anymore [45]. The returned result is locally optimal. For all cases we requested 500 clusters, with maximally 1000 iterations (no issues of non convergence), and 25 random sets to start with. The underlying method is the algorithm of Hartigan and Wong [46]. Each structure was labelled by its origin being either from MD or a generator. The RMSD for each cluster was calculated based on the cluster members’ respective radTorsion data for each cluster member against each cluster member, max and median RMSD were saved. The overall max RMSD is defined as the max of all clusters’ max RMSD. The overall median RMSD is defined as the median of all median RMSD.

Statistical analysis on the significance of the differences of the post-optimized MD conformer and generator maps was performed to obtain p-values with the function betadisper [47, 48] from the R package vegan on the Euclidean distances of the respective latent variables’ coordinates. A multivariate permutation test was performed for the homogeneity of group variances using the function permutest from the same package with 10,000 permutations, pairwise comparison was set to ‘TRUE’. Note that using this permutation method with a set number of permutations computed p-values cannot be lower than 1 * 10^–5^. Utilizing the TukeyHSD function from the same package, confidence intervals for the difference between the group’s respective mean distance-to-centroid are calculated. The difference is always defined as $\mathrm{\Delta }\mathrm{D}={D}_{miniMD}-{D}_{generator}$, whereas $D$ is the mean distance-to-centroid.

The 3D polar surface areas [49] as derived in Pipeline Pilot [12] are color-coded with thresholds of 95 ± 17.7 Å^2^ and 145 ± 21.4 Å^2^ based on the correlation (slope: 1.01; intercept 5.32) between 2D TPSA and 3D PSA values for 10,000 randomly selected compounds from the Aldrich Market Select catalogue (Additional file 1: Figure S1), by using the TPSA thresholds regarding oral absorption and bioavailability considerations as published [50–52].

Further color-coding options applied include starting conformer, solvent, simulation temperature, simulation run number.

## Results and discussion

In this work, the diversity of conformational ensembles was analyzed via a map derived from principal component analysis of the torsional space of the macrocyclic ring atoms. We did so to avoid any ambiguities from root-mean-square error (RMSE) calculations for the macrocycle atoms due to structural alignment algorithm used. Apart from this, our approach allows to create one consistent map from all six 16-membered ring macrocycle structures based on all MD and SA snapshots. We excluded side chain dihedrals since we expect any algorithm to be able to comprehensively sample this torsional subspace. The map was created from the 32-dimensional space spanned after transformation of dihedral angles into sinus and cosinus values, avoiding discontinuities in mapping.

Torsions of all conformers of compound **7** as well as of all conformers from the conformer generators for compounds **1** to **7** were projected onto the global map defined by compounds **1** to **6**. The global map was derived from all snapshots for neutral and positive charge state in the three solvents at 300 K and from the simulated annealing runs to allow direct comparison between molecules, solvents and charge states. The combined PCA map from **1** to **6** provides accumulated variance of 15.1, 27.7, 37.6 and 45.2% for PC1 to PC4, respectively. We loose information with respect to the maps created from the conformers of any individual compound (between about 37 and 48% accumulated explained variance for PC1 and PC2, cf. Additional file 1: Table S1), but at the same time we yield comparability.

**Fig. 1:**
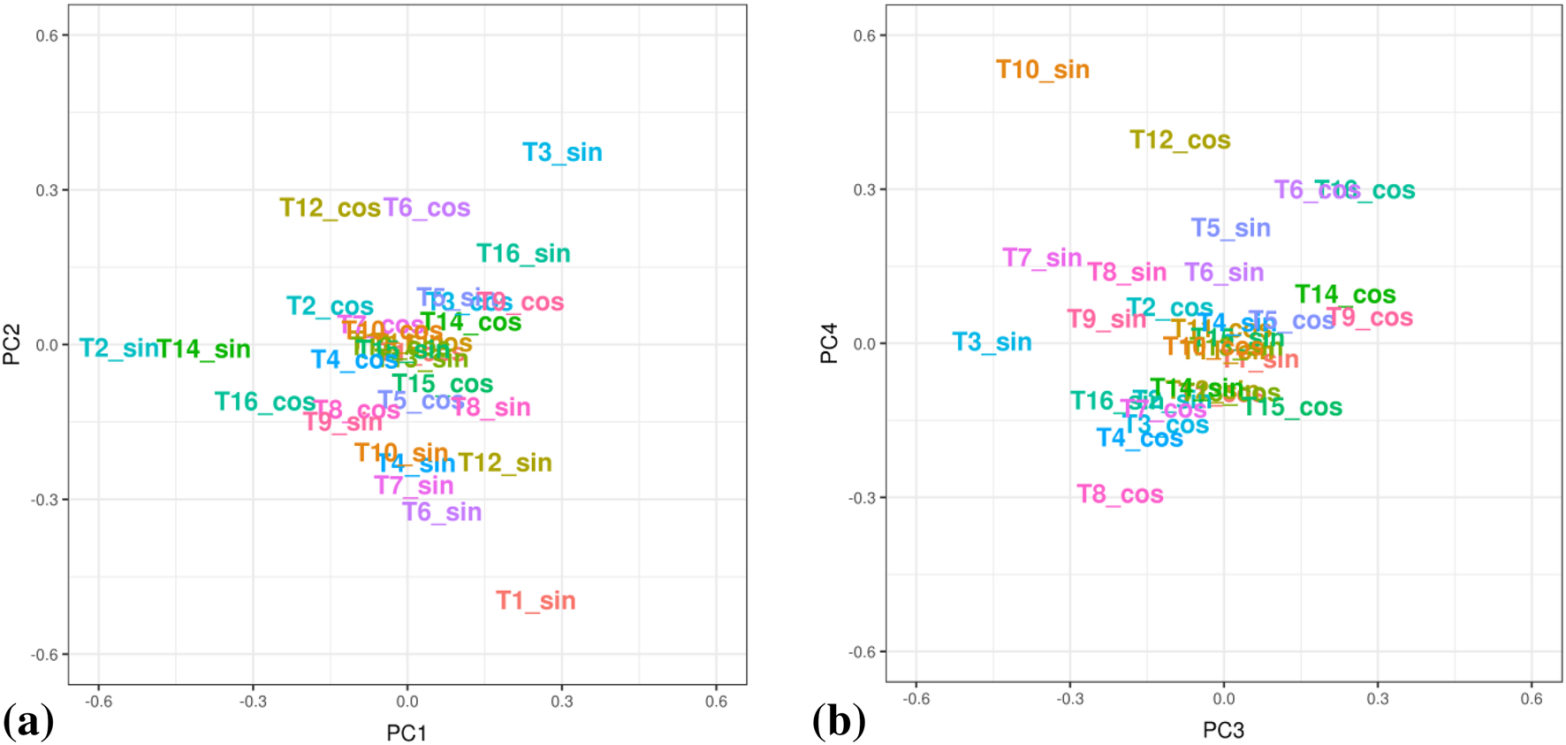
PCA loading plots for **a** the latent variables PC1 and PC2 and **b** latent variables PC3 and PC4 for the 32-dimensional space spanned by sinus and cosinus values of the 16 torsional angles as defined in Scheme 2

Figure 1a shows the loading plot for PC1 vs. PC2 and Fig. 1b for PC3 vs. PC4 and Scheme 2 the definition of the torsional angles T1 to T16. The loadings inform about the relevance of the input descriptors for the spread of the latent variables. Major contributors for PC1 are sin(T2) and for PC2 sin(T1), sin(T3), and to a lesser extend sin(T14), cos(T12) and cos(T6). Linker torsions sin(T10), sin(T7), cos(T8) show some significance for PC3 and PC4 only. T2 and T14 define the flexibility of the central amino acid, whereas T1 connects linker and amino acid 1 and T3 connects amino acids 2 and 3. The torsion angle distribution profiles (see Additional file 1: Figure S2) for sin(T1), sin(T2), and sin(T3) are dominated by the extremes -1 and 1, i.e. 90° and 270°. To a lesser extent, this is true for the other torsions being relevant for the spread of the loading plots. Nevertheless, one has to keep in mind that the map is only a low-dimensional projection and also that the torsions identified are the ones that define distinct conformations but not torsions that define flexibility by itself. Some of the profiles look more like the ones expected from atrop-isomers.

In the following paragraphs we will first compare the individual macrocycle maps for both charge states in solvent water. Next, on the example of macrocycle **1**, we will demonstrate the influence of starting coordinates, i.e. multiple starting conformers versus simulated-annealing run, on the conformer landscape. Third, we will look into population differences depending on the solvent.

We will then map the conformer generator ensembles onto the MD-derived maps and discuss ensemble completeness based on mapping overlap, conformer energy overlap, intramolecular hydrogen bonding and polar surface areas.

### Ensemble completeness of molecular dynamics snapshots

#### Dependence on input coordinates

**Fig. 2:**
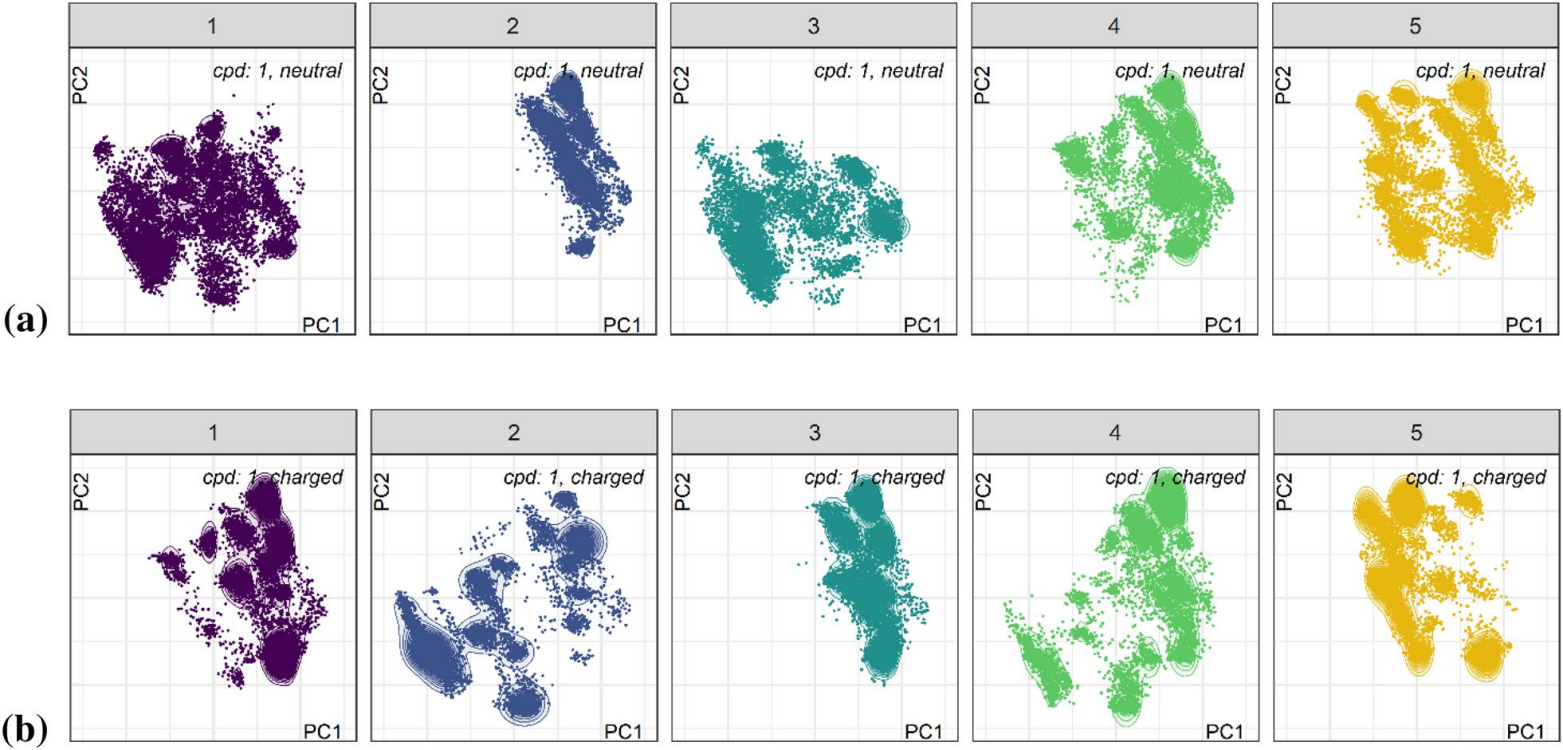
Maps for compound **1** resulting from five 100 ns MD runs at 300 K in solvent SPC water; **a** neutral, and **b** positively charged. Color-coding reflects starting coordinates

We expected the macrocycles to be quite rigid compared to typical small molecule drugs. But since cyclization is the last synthesis step one can expect that due to variable preorientation before actual ring-closing multiple sub-ensembles each “frozen” in a deep potential energy well may co-exist. Therefore, we ran five 100 ns MD simulations at 300 K in the solvents water, DMSO and CHCl_3_, from five diverse starting conformations obtained from the BEST ensemble. For any molecule we were able to confirm that each starting conformer covers only a subset of the overall conformer space. Figure 2 shows two exemplary sets of maps for neutral and positively charged compound **1** in water, color-coded by the starting conformers (consider that spaces respective dots overlap partially).

#### Dependence on charge state

**Fig. 3:**
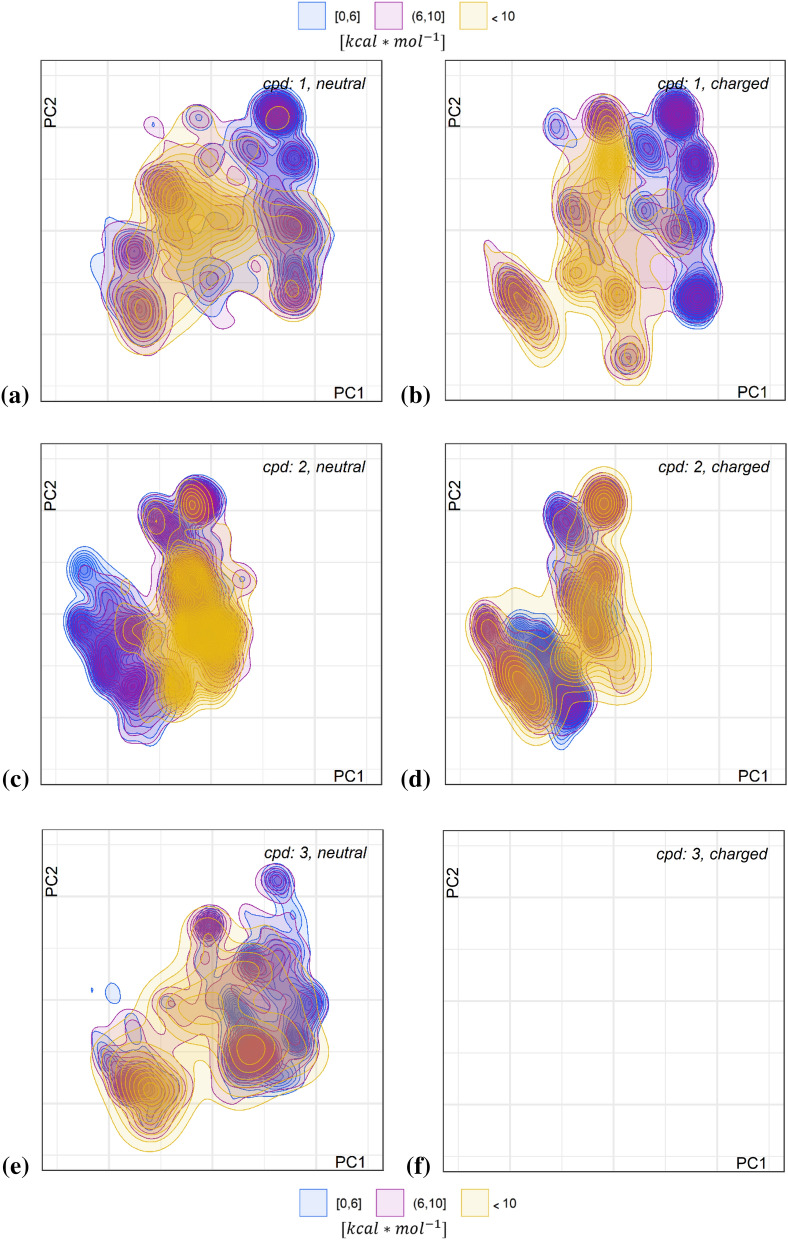

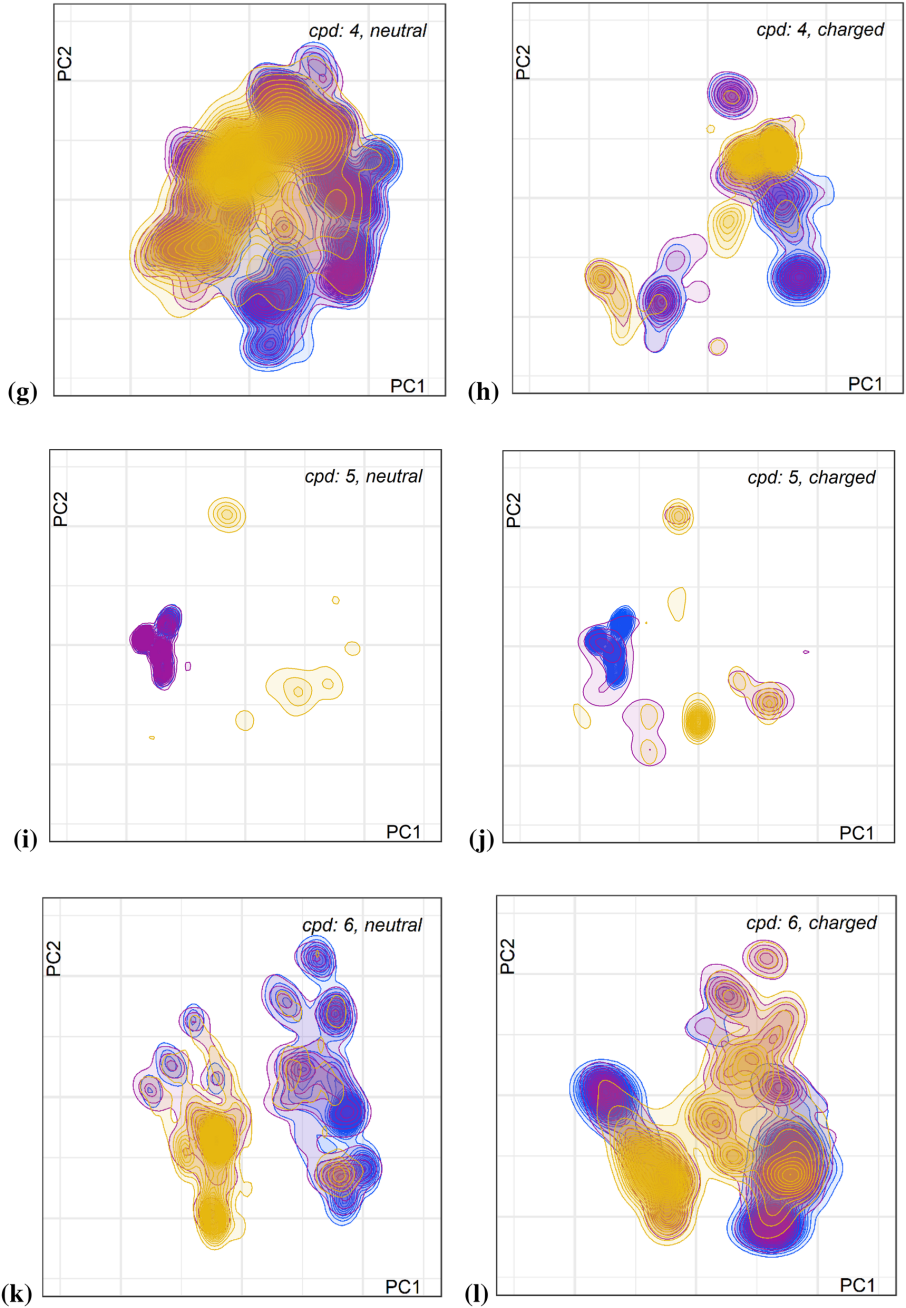
Conformer density maps for six macrocycles in solvent water, color-coded by binned raw conformer relative energies with thresholds < 6.0 (blue), ≤ 10.0 kcal mol^−1^ (pink) and > 10.0 kcal mol^−1^ (orange). Conformers with relative energies higher than 100 kcal mol^−1^ were filtered out. Plots **a**, **c**, **e**, **g**, **i**, **k** in the left column show maps for neutral and plots **b**, **d**, **h**, **j**, **l** in the right column for positively charged ligands (charged ligand **3** is missing due to *N*-methylation)

From Fig. 2 one can see that the maps look different for neutral and positively charged ligands. We explore this behaviour further in Fig. 3 providing the energy landscape maps (line and color density reflecting density of conformers) for either neutral or positively charged macrocycles **1** to **6** (Note that **3** is *N*-methylated at amino acid 3, the phenylalanine, and therefore is not protonated) derived from 50,000 snapshots of five simulations with diverse starting conformers. Color-coding here is by binned OPLS3e energies for the not post-optimized snapshots and reflects the accessible conformational space for the compounds in explicit water which is significantly larger than that for the local minima (more on this later). The snapshot energies obtained in implicit solvent after stripping off the explicit water box are binned into three groups with thresholds of 6 and 10 kcal mol^−1^ (25.1 and 41.8 kJ mol^−1^) as deduced from literature (note that there are multiple controversial values discussed) for biorelevant conformations [9].

One must be aware that these energies are not comparable to the energies obtained for post-optimized structures, since they always contain an unknown portion of the thermal energy from the MD simulation of the ligand-solvent system. In the final section of this work, we will compare the conformer energy distributions of the post-optimized and RMSD-clustered snapshots with the distributions derived from the ones from conformer generators.

The maps clearly show that each macrocycle has its distinct conformational profile and that the profile is also dependent on charge state. This indicates that a change in charge state which is a prerequisite to cross a lipophilic cell membrane, will be more probable for structures with highly populated overlapping low energy conformer ensembles between charged and neutral states. For most structures, the map for the charged ligand is more constrained, with the unexpected exception of compound **2**, which differs to 1 only by the change in stereochemistry from l-Leu to d-Leu. Drastic changes in the minimum conformations upon change in one stereogenic center were previously also reported by the group of Lokey [20], which allow us to expect the observed differences in the ensemble maps. Also, the distribution between low, medium and high-energy conformations differs between compounds, and more pronounced between charge states. Compound **5** exhibits mostly high-energy conformers (for both neutral and charged state) and only small restricted low energy islands, indicating incomplete sampling, as discussed in the next section. Methylation of Leu in position 1 restricts the overall flexibility much more than the other N-methylations.

Analysis of the respective maps for the other solvents (Additional file 1: Figures S3, S4) reveals a certain tendency for more constrained maps for charged compared to neutral ligands in DMSO but not in CHCl_3_. We explicitly mention here that protonation of macrocycles will probably not play a role in DMSO and CHCl_3_ experimentally_._ Nevertheless, the computer experiment allows to get insights as far as the force field parametrization is meaningful in this respect.

Overall, we can conclude that the population of conformational space significantly differs for any ligand between the different solvents.

**Fig. 4:**
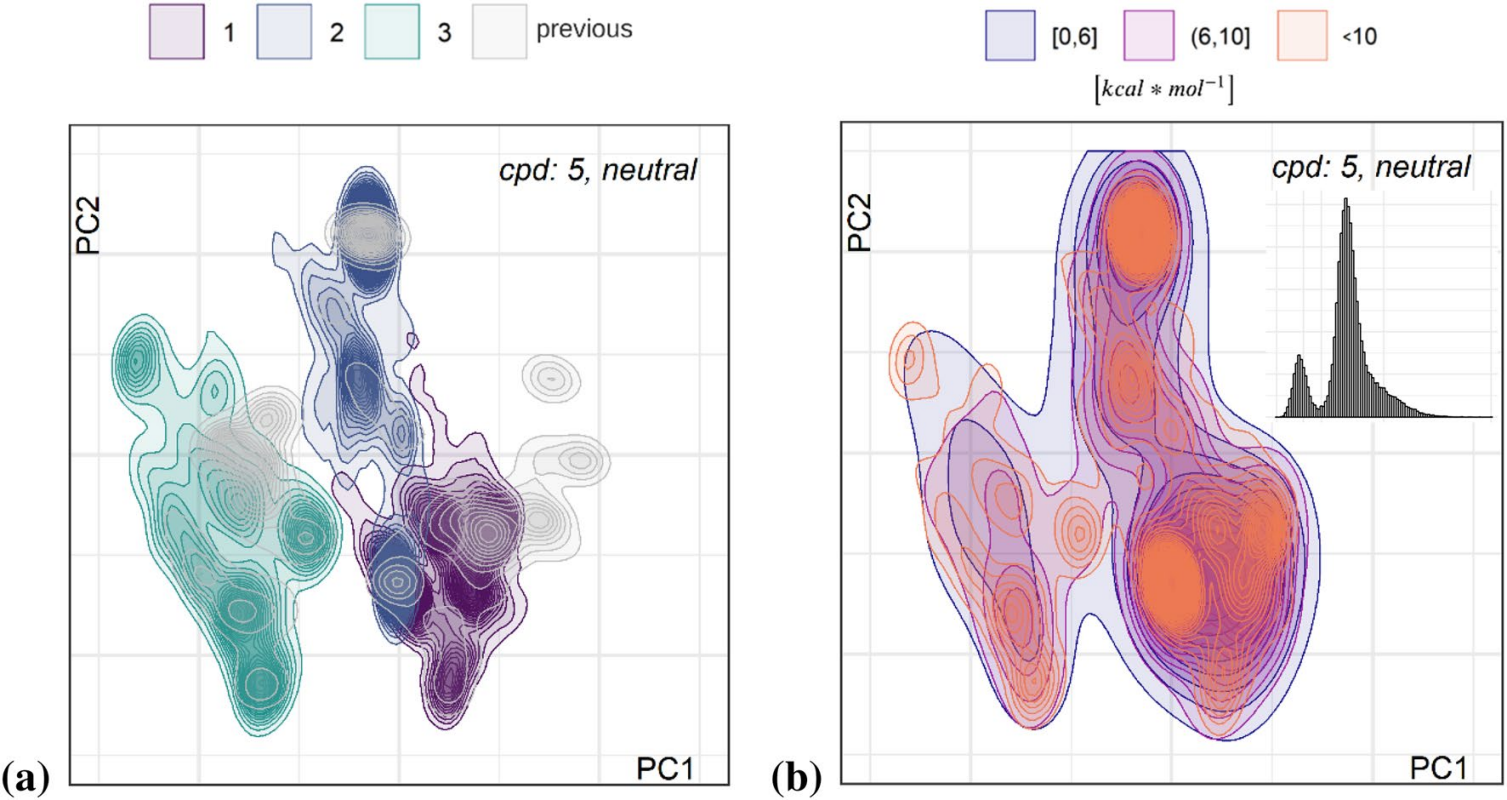
Conformer map for additional MD runs with three low energy starting conformers of compound **6** in solvent water. **a** color-coded by starting conformer; **b** color-coded by binned raw relative energies with thresholds < 6.0 kcal mol^−1^ (blue), <  = 10.0 kcal mol^−1^ (pink) and > 10.0 kcal mol^−1^ (orange). Conformers with relative energies higher than 100 kcal mol^−1^ were filtered out; the insert in **b** shows the distribution of snapshot energies over 8 MD runs (energy range 40 kcal mol^−1^)

The map for compound **5** is very restricted and dominated by high energy snapshots with small low-energy islands, which hints to incomplete sampling. We therefore performed three additional MD runs with diverse low energy starting conformers from the PMM generator. The runs yield three distinct ensembles (Fig. 4a) with raw energy distribution on the map (Fig. 4b) more comparable to the other compounds. The overall energy distribution for snapshots from 8 runs shows two peaks (see insert) centered around 5 and 15 kcal mol^−1^ compared to distributions with only one peak for the other macrocycles (see Additional file 1: Figure S5), and even for the three additional runs the distribution between low and high energy snapshots is inverted compared to all other macrocycles.

Though the maps for all compounds except **5** appear to be complete regarding sampling, exhibiting similar shapes and balanced distributions of snapshot energies, we nevertheless performed some more experiments.

To test for the effect of sampling time, we additionally ran 10 simulations of 1000 ns each for compound **2** in CHCl_3_. Longer sampling did not yield new conformational states, suggesting that our standard settings are appropriate, whereas additional diverse starting points are needed in some cases, as shown for **5**. Alternatively, we test for the risk of partial coverage of torsional space not caused by sampling time, but by too high barriers to be overcome at 300 K. Simulations for 100 ns at 400 K and 500 K yielded increasingly more overlap between the regions covered by the different starting conformers, providing evidence for high barrier hypothesis (at 800 K the simulations stopped after some time due to evaporating solvent), but also proof that even elevated temperatures do not allow to sample with only one starting conformer as shown in Additional file 1: Figure S6.

To further confirm the hypothesis, we did simulated annealing molecular dynamics runs with 10 heating and cooling phases up to 500 K for the lowest energy conformer of each molecule, and exemplary also with multiple starting conformers for positively charged **2** in water (see Additional file 1: Figure S7). We found that (i) there were no new basins explored anymore after four to five heating and cooling phases and (ii) the conformational space explored is significantly smaller with missed areas on the map, compared to diverse starting conformer MD runs. Our setup thus allows for exchange between neighbor basins but probably many more cycles and higher temperatures would be needed, making the diverse starting conformer setup the method of choice.

Based on our findings for compound **5**, we emphasize here that we cannot provide final evidence that we were able to identify complete ensembles by our approach. The similar proportions of low, medium and high energy conformation snapshots when applying higher temperature, longer simulation time and simulated annealing suggest complete or near-to-complete sampling, but there is no rigorous approach to quantify completeness.

#### Dependence on solvent

We performed the simulations in three solvents, namely SPC water, DMSO, and CHCl_3_, with dielectric constants [53] ε of 78.35, 46.83, and 4.71, respectively. Based on the ε values, and that water is a polar protic and DMSO a polar aprotic solvent, we speculated the conformational space in DMSO to be somewhere in the middle, but with more overlap to water.

**Fig. 5:**
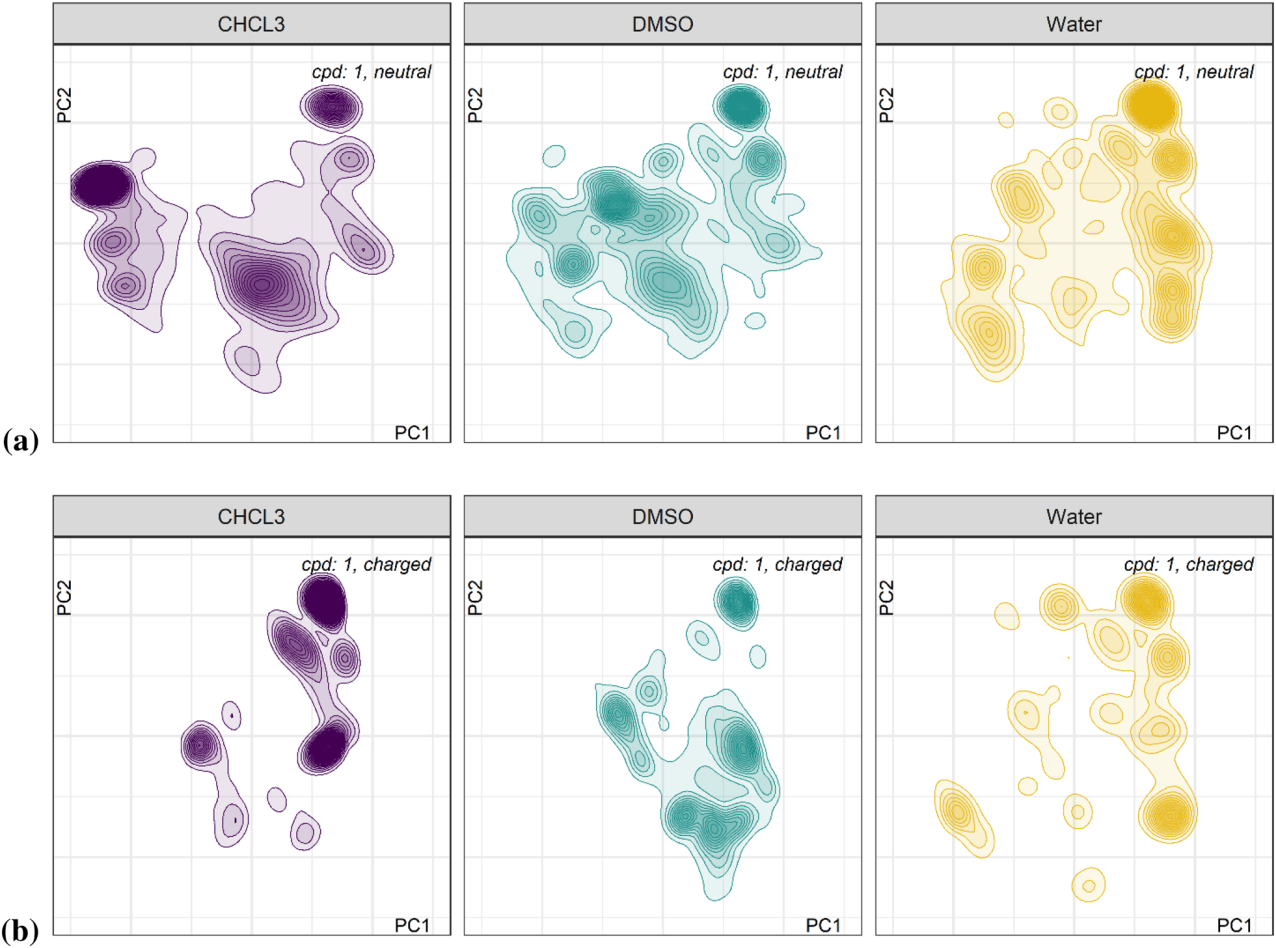
Density maps for distributions of accumulated conformers derived from five MD simulations at 300 K with five different starting coordinates for **1** in solvent water (yellow), DMSO (green), and CHCl_3_ (purple), **a** for neutral and **b** for positively charged species

The conformer distributions for the three solvents for neutral and charged ligand **1** are shown in the density plots of Fig. 5. The plots provide information on the density of conformations at each point in latent space similarly to a geographical map.

The denser the lines, the more populated. For solvent water there are densely populated areas and distinct islands, and overall only one area of dense overlap between conformers from all solvents, in the upper right corner (latent coordinates of center: 1.3; 2.4). There is more overlap between water and DMSO conformational space. The situation is similar for the neutral and charged species, but with more pronounced water islands for the charged case. We here stress again that experimentally the charged species will not be existent in the organic solvent and play only a minor role in DMSO, whereas in silico we can look into the solvent dependence of such states. The observations can be generalized to the other compounds (cf. Additional file 1: Figure S8).

The clear separation of conformational spaces in water and chloroform indicates that there will barely be any metastable states pre-formed in water that would allow for rapid entrance and permeation through cell membranes, if we follow the conclusions from the work of the Riniker group [1, 54, 55]. Nevertheless, to test this hypothesis, we would need to apply Markov-state modelling based on much larger numbers of diverse starting conformers.

Our motivation to perform MD simulations also in DMSO was that this solvent plays a major role as solvent in pharmaceutical research, especially in NMR experiments as the ones performed in our earlier work [13]. Since DMSO based results are always somewhere between water and chloroform results and since there is no implicit solvent model for DMSO for OPLS3e, we decided to not further include DMSO results here.

### Ensemble completeness of conformer generators

In the previous section we described the general shapes and properties of the conformational space accessible to the compounds at room temperature as simulated by molecular dynamics. The said space of conformations spanned from 50,000 MD snapshots is significantly larger than the space of minimized conformers generated by conformer search algorithms. To make the spaces comparable, the MD snapshots were all minimized in implicit solvent, always resulting in a collapse of various MD snapshots onto one local minimum conformer. Nevertheless, the plots are still dominated by MD snapshots. No attempt on a meaningful clustering by different algorithms was successful due to the many smaller clusters we would have lost. We therefore decided to stay with the original set sizes.

#### Torsion map overlaps

**Fig. 6:**
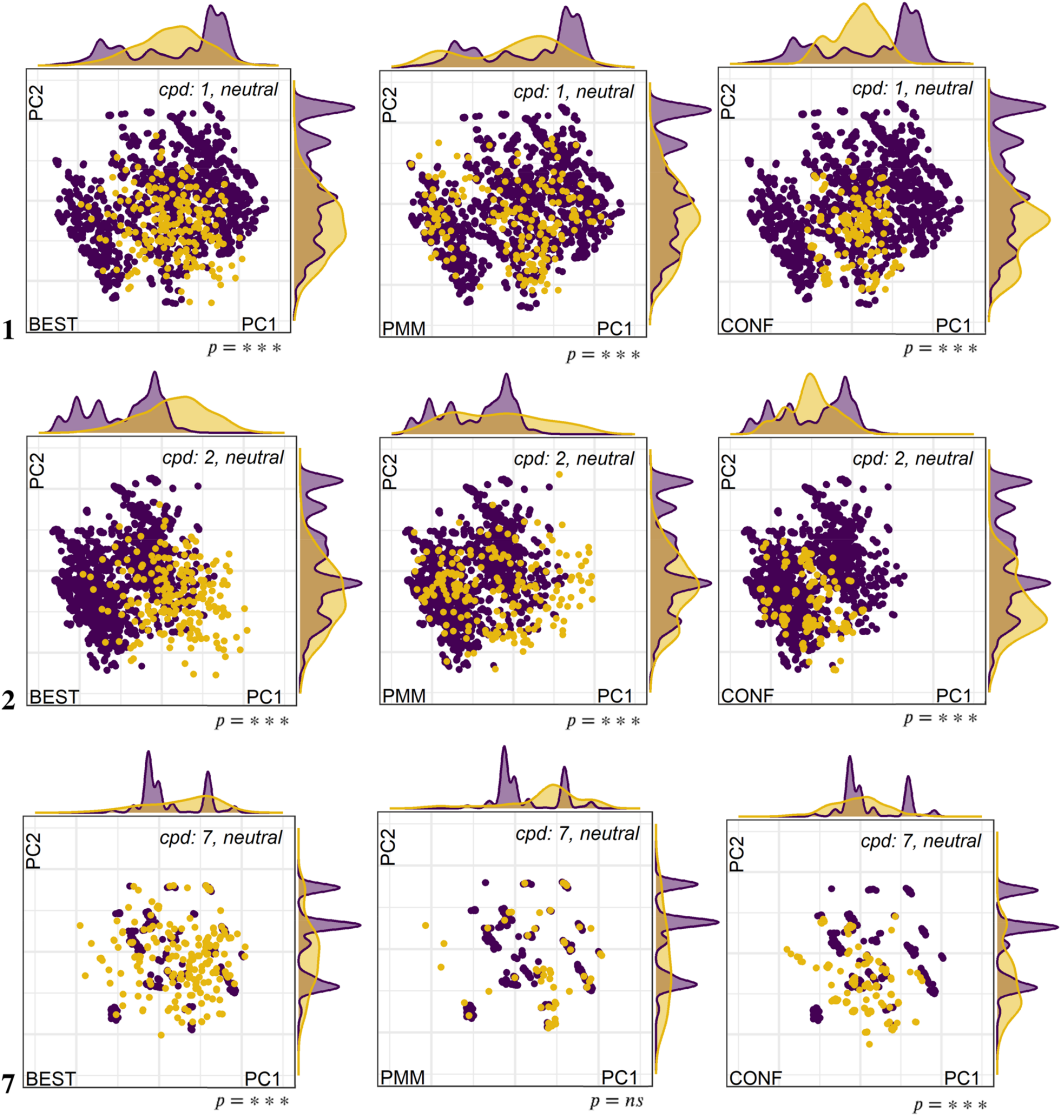
Maps of latent torsional space accessed by post-minimized MD snapshots (5 starting conformers, 300 K, 100 ns each) for neutral compounds **1**, **2** and **7** in SPC water in purple, overlaid with conformers created by BEST (left), PMM (middle) and CONF (right) in yellow. Asterisks indicate level of significance (*** = p-value < 0.0001, ** = p-value < 0.001, * = p-value < 0.01, ns = non-significant)

Overlays of the post-optimized snapshots from the MD with the geometry optimized sets from BEST, PMM and CONF allow to visually compare the coverage of torsional space. Optimizations were performed using the implicit solvent models for water and chloroform provided with OPLS3e. Figure 6 shows the overlay plots for neutral molecules **1**, **2** and **7** in water, and Additional file 1: Figures S9–S11 the respective plots for all seven macrocycles in water (neutral and charged) and CHCl_3_.

For compounds **1** to **6**, the general observation is that the space covered by MD is larger than for the other methods, at least in the 2-dimensional space of the first principal components. In the following we will discuss in how far our conclusions are true if considering the other 30 dimensions. We observe that the coverage of the maps is highest for PMM, followed by BEST, and most restricted for CONF. The deviations between the torsional spaces covered are highly significant for almost all maps (p-value < 0.0001), with significantly higher mean distance-to-centroid for all MD maps (see also Additional file 1: Table S4). The exception is **2** with significant uncharted territory explored by BEST and PMM compared to MD, but at the same time BEST missing a large part of the MD space. The same is true for the charged species in water and the neutral one in chloroform. This was totally unexpected, since **1** and **2** are enantiomers. Non-overlapping PCA map areas are seen also for compounds **5** in chloroform and positively charged **7** in water (cf. Additional file 1: Figures S10, S11).

Another major finding that is even more critical for the application in drug design, is that the post-optimized conformer maps from BEST, PMM and CONF are highly similar independently of solvent and charge state, whereas the MD-derived maps show the expected variability. Whereas explicit solvent molecules interact with each other and the solute and by this influence preference of conformational states, implicit solvent models do only modulate the energy function during optimization. Therefore, one can probably not expect to identify solvent-specific low-energy conformers for a particular charge state with a conformer generator if the raw conformer is not preferable based on the “scoring function”, may it be some gas-phase like energetics or RMSD or whatever is used.

Since one could argue that the plots in Fig. 6 could be biased by the imbalance of the number of conformations between MD and the generator methods, we attempted to create the same number of 50,000 raw unoptimized conformations for each method. Even though we stretched settings up to extreme, beyond meaningful values like e.g. an energy threshold of 100,000 kJ mol^−1^ in case of BEST, we were still not able to arrive at the desired numbers. The computation times increased significantly to multiple hours per run. We obtained only 6,337 conformations for BEST, 12,142 for PMM (38,897 redundant ones that were automatically reduced to the final number), and 24,775 in case of CONF. The respective plots are shown in Additional file 1: Figure S12. The plots clearly indicate that the latent space covered by all generator methods is smaller than that of the MD snapshots, with ranges of about − 1.5 to 2.3 for PC1 and − 2.7 to 2.1 for PC2 of the BEST and PMM maps, compared to about − 2.6 to 2.8 for PC1 and − 2.7 to 2.5 for PC2 of the MD snapshot plot. The map for CONF is drastically different in that it shows only about 20 distinct ring shapes, which means about 1000 side chain conformations obtained per distinct ring conformation.

#### Quantification of the map overlaps

In the last paragraph, we provided the qualitative picture based on the 2-dimensional overlap maps. The question is now in how far a quantification of the map overlap is possible. In the following we provide three measures to quantify the overlap, namely statistics on mixed and unique clusters, variance statistics, and a novel metric, the Mahalanobis distance for the coverage of torsion space.

As a first metric, we tried k-means clustering for each combined set of post-optimized conformers from MD and generators for neutral and charged state in water and CHCl_3_ (for more details and results see Additional file 1: Table S3). With exception of the charged state of the smaller and more rigid macrocycle **7**, we find significantly less mixed clusters for CONF than for BEST and PMM. Overall, we have to state here that a reliable quantification of map overlap, and not even a qualitative description, is at all possible by clustering, especially given the strong dependence on arbitrary parameters like required cluster size or RMSD.

An alternative to clustering that is not dependent on the method and its settings is the quantification of the variance explained by the PCA projection used for the maps. There are two metrics commonly used, namely the total variance and generalized variance, i.e. the trace and the determinant of the eigenvalues of the covariance matrix of the latent variables, the latter generally being interpreted as the volume of the point cloud [56].

Though it is sometimes claimed that only the metrics considering all principal components are able to describe the ensemble variance, one has to keep in mind that especially higher order principal components might be misleading. Since any geometry optimization is determined by the energy threshold applied, the numerical precision of the dihedral angles obtained will introduce some numerical noise. Such expected smaller variance is also captured, most likely within the higher order principal components. And that is exactly what we see in the total and generalized variance plots.

**Fig. 7:**
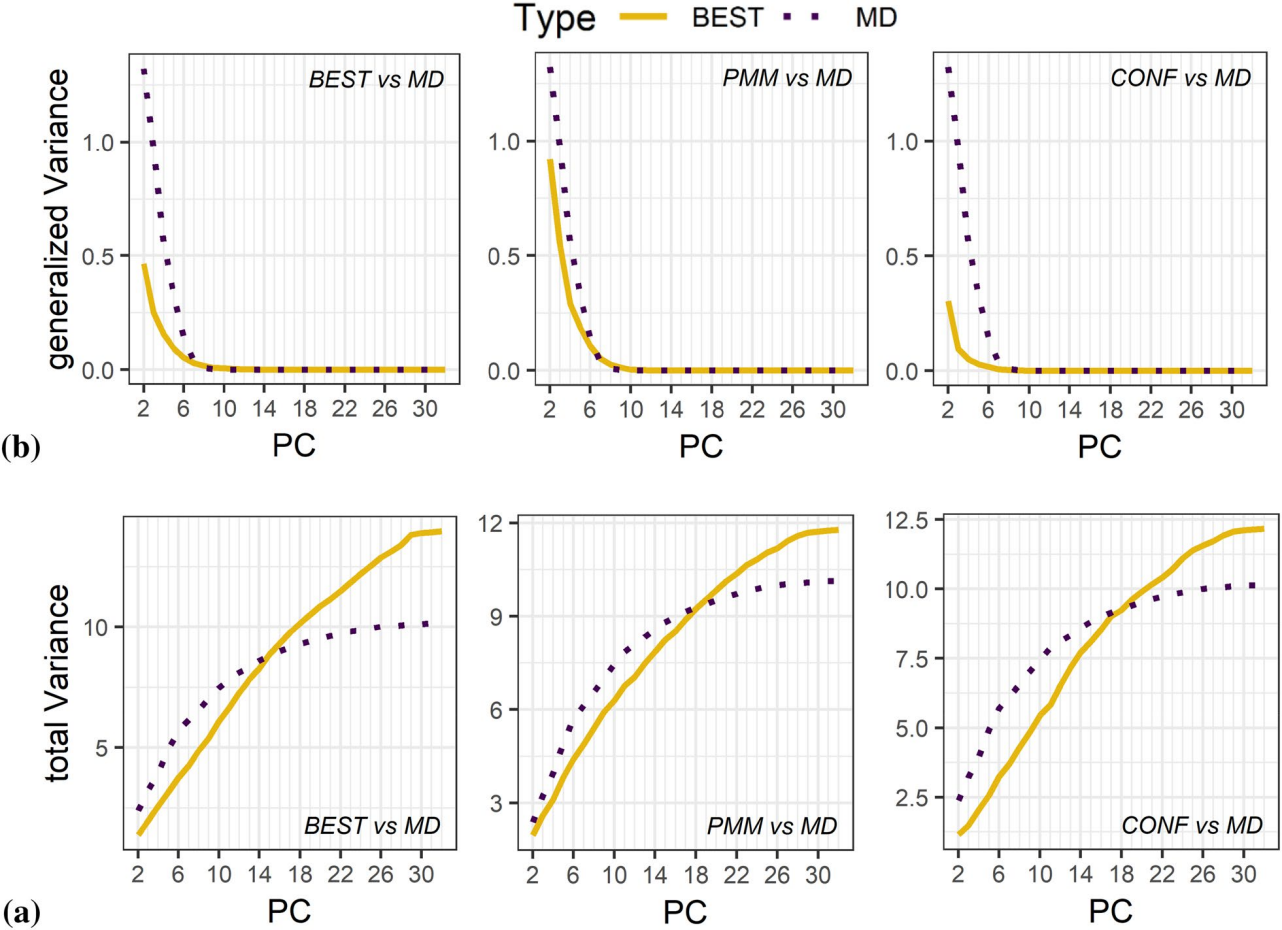
Plots of the variance explained from the PCA analyses. **a** Reported is the generalized variance vs. the numbers of the latent variables used for the three methods BEST, PMM and CONF in comparison to the total variance of the MD simulations; **b** total variance vs. the numbers of the latent variables

The generalized variance plots (Fig. 7a) yield curves that start at higher values and are much steeper for MD, i.e. more variance in earlier PCs than for generator methods as one would have expected by the design of the principal component analysis. After inclusion of about seven to eight latent variables the generalized variances virtually reach zero, with final values of 10^–20^ and below. The generalized variance thus turns out to not be very useful because of the linear dependencies in the transformed dihedrals, resulting in near zero eigenvalues.

Figure 7b on total variance, i.e. the trace of the covariance matrix, analogously shows an earlier and steeper increase for the MD curve than for the generator curves but a crossing of the curves at 14 (BEST), 18 (PMM) or 17 (CONF) latent variables used for the total variance calculation. Perfectly in line with plots of Fig. 6, for the earlier PCs that carry most of the information (67% for PCs 1 to 8) on conformer diversity the total variance is higher for the MD conformational space than for the others. The crossing of the curves was nevertheless unexpected. To identify the root cause of the crossing, we created plots of each pair of latent variables for the example of neutral compound 1 in water (Additional file 1: Figure S13). We find that after 10 latent dimensions, the spaces of the generator and the MD conformers start to separate more and more. As those PCs contain 3% and less of the information content of the conformer space we suspect that such observed variances display more likely uncertainties than meaningful variation.

To further investigate if that crossing we observe really reflects the noise from the spurious contributions of the higher latent variables, we added artificial normal distributed noise (mean = 0, sd = 2 * pi/180) to the dihedrals. Respective plots are shown in Additional file 1: Figure S14. The trace for the minimized MD conformers describes a logarithmic behavior just like the accumulated variance of the entire space. This is expected as the underlying PCA space is based on the raw MD conformers which are obviously closely related to the minimized samples. Also, as observed already in Fig. 7 the BEST trace describes a more linear curve. This is most likely due to the fact, that the principal components are constructed to maximize the variance of the raw MD conformers. Adding noise results for both sets in reduced traces and in a linearization of the minimized MD trace, affecting the minimized MD trace stronger due to the much higher number of samples with added noise and is therefore difficult to interpret. However, the differentiation between curves with and without noise for the generator starts after the first eight latent variables, that we consider to carry relevant information.

Both, total and generalized variance provide some indication that the diversity of MD conformer space is indeed higher than for the generators. The variance is concentrated on the early PCs. The starting values of the generalized variance plots indicate the order PMM > BEST >> CONF in accordance to maps in Fig. 6 and the cluster analysis, whereas the curves for the total variance are more or less identical. Since both total and generalized variance do not provide the desired quantification, we looked into a third alternate metric.

We here apply a concept from machine learning, namely the Mahalanobis distance [57] which is a measure for outliers and thus for the applicability domain of models for a specific data point. The idea here is that we define the conformer space from the generators as the “training set” and the conformer space of the post-optimized conformers from MD as the “prediction set”. The more extended the MD space is compared to the generator space, the more “outliers” and the higher the median and maximal Mahalanobis distances.

The Mahalanobis distance is defined as

$$D{M}_{M}\left(x\right)=\sqrt{{\left(x-\mu \right)}^{T}{S}^{-1}(x-\mu )}$$

With x = ( x1, x2…xN) and µ = (µ1, µ2, … µN) being the latent variables (principal component coordinates, i.e. cartesian coordinates) derived from the original 16-dimensional torsional space (as transformed to 32 sin and cos values) of either a specific conformer or the mean vector of the “training set” and the covariance matrix S. We stress here that for each dimension, i.e. number of latent variables considered, the Mahalanobis distance is scaled independently and the absolute numbers are not strictly comparable. Nevertheless, since the principal components decrease when adding more and more components, and each PC is greater equal zero, the curves will increase monotonically. Both the MD maps and the generator maps are projections on the PCAs from all raw conformers (we define the more restricted map as “training set”).

**Fig. 8:**
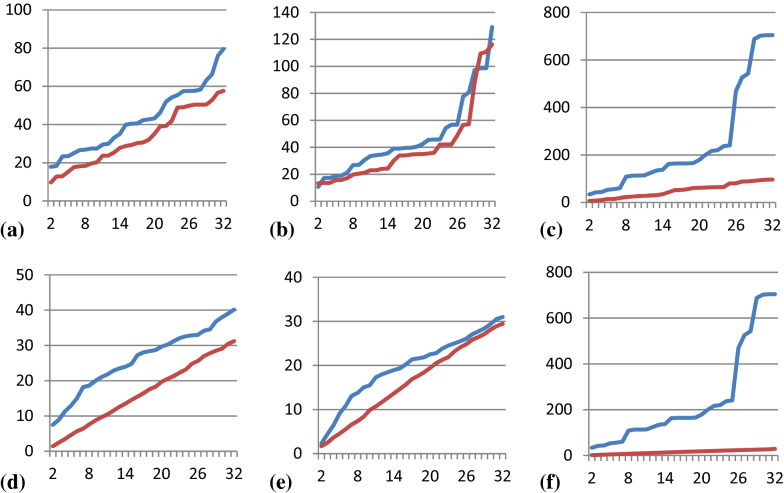
Plots of maximum (top row) and median (bottom row) Mahalanobis distances (y-axis) for generator conformers (red) and MD conformers (blue) for increasing numbers of PCA latent variables (x-axis; between 2 and 32) for neutral compounds in water. Plots **a** and **d** show Mahalanobis distances for BEST conformers, **b** and **e** for PMM conformers and **c** and **f** for CONF conformers

Figure 6 shows that the MD conformers span a larger space in the first two PCA latent dimensions than the generator conformers. In principle, this could be misleading, since the first two dimensions explain only a fraction of the overall variance. Therefore, we calculated the Mahalanobis distances for increasing numbers of latent dimensions between 2 to 32, regardless of the results from the variance statistics that would indicate to stop at eight dimensions. Figure 8 shows the maximum Mahalanobis distance of any conformer to the center in the top row and the median Mahalanobis distance in the bottom row, and to the left BEST, in the middle PMM and to the right CONF. Since we did not normalize the data, the scales are different between plots but comparable for the two series displayed on each plot. Larger populated shapes will overall result in similar Mahalanobis distances of their members as smaller similarly populated shapes, since we always compare the center and data distribution of the “local model”.

For any number of latent dimensions and any dataset (with the one exception of dimensionalities of 30 and 31 for PMM), the Mahalanobis distances for the MD conformers are always higher than the distances of the conformers for the “training sets” derived from the generators, and the median values are always about half or less of the maximum values. The median Mahalanobis distance values for the conformers for all three generator methods are roundabout 30 for the 32-dimensional space, i.e. the core regions of all spaces are similar in size. The maximum Mahalanobis distances on the other hand differ considerably, with 58 in case of BEST, 116 for PMM and 96 for CONF. Though not quantitatively comparable, this is a hint that the BEST space is overall more compact and that PMM and CONF at least have islands of distinct conformations.

If we now compare the Mahalanobis distances for the MD conformers with the ones for the generator conformers we find that the MD conformer covered multi-dimensional shapes are always larger. In the cases of BEST and PMM the distances are more or less shifted in parallel, whereas in the case of CONF there is a tremendous distance increase for spaces higher than 7-dimensional. We only observe this change in profile but have no explanation.

One could argue that Mahalanhobis distance did not indicate the amount of spread were larger for MD, but only that the MD set were different from the conformer generators in some way. We again refer to Additional file 1: Figure S13 that provides the individual maps for compound 1 that shows that the projections indeed overlap in space for all latent dimensions that carry significant information.

In summary, the plots confirm that BEST and PMM show a better overlap with MD conformers than the very compact conformer ensemble from CONF. Nevertheless, none of them is a complete ensemble. Again, we explicitly state, that there is no guarantee that the MD ensembles, though more diverse, are complete.

Finally, we should mention that in this work we provide strong evidence that it is important to consider typically more than 2 latent dimensions to represent the data correctly. In the past there were many publications on mapping of chemical spaces like ChemGPS [58], protein–protein interaction ligands [59], or modeling of solubility [60] that did not address this issue.

#### Intramolecular hydrogen bonds

It is common understanding that compounds capable of forming intramolecular hydrogen bonds (IMHB) will do so more likely in apolar solvents to expose their lipophilic surface, whereas in polar and especially protic solvents they will expose their donor and acceptor functions. The phenomenon is frequently referred to as chameleonic behavior [61, 62]. We therefore calculated the numbers of IMHB for each ensemble of snapshots, based on slightly relaxed angle and distance constraints to account for the non-minimum nature of the snapshot coordinates.

**Fig. 9:**
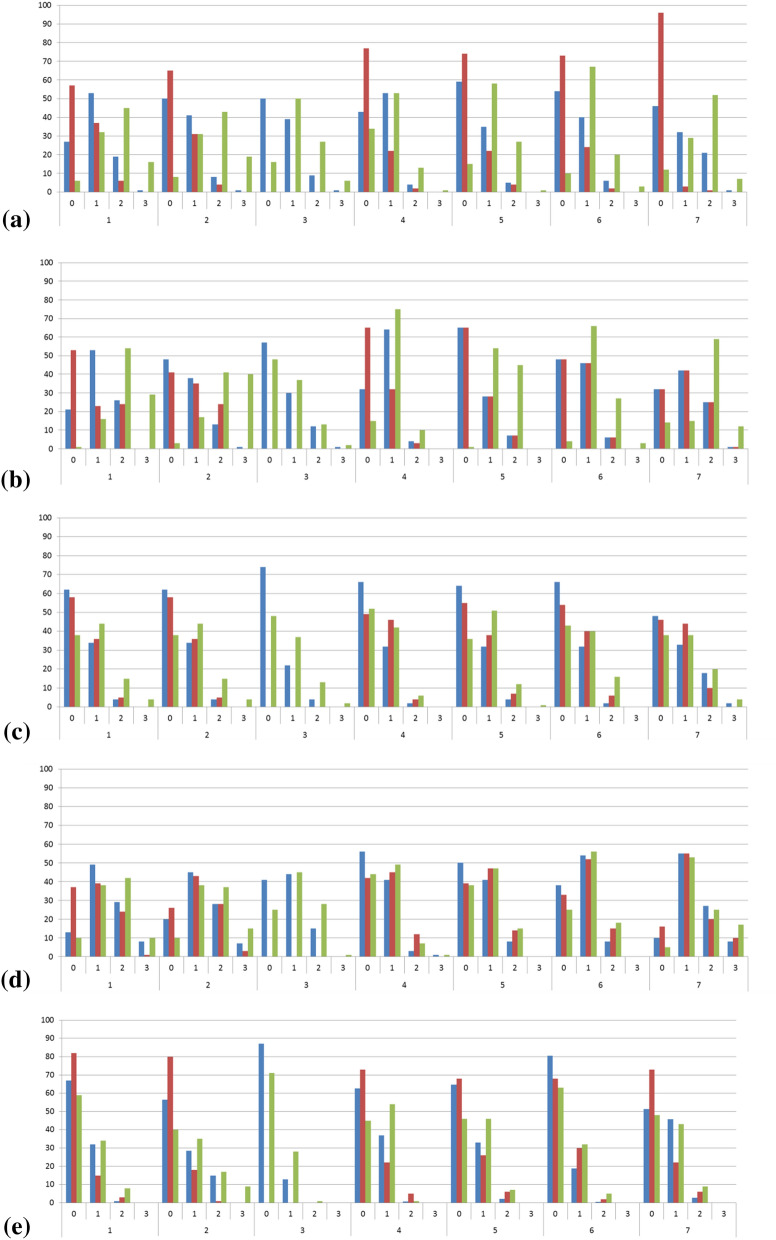
Histograms of percentages of intramolecular hydrogen bonds (0 to 3 IMHB, top x-axis labels) for the conformers of molecules **1** to **7** (bottom x-axis labels) as derived from five MD simulations at 300 K with five different starting coordinates in different media; blue bars for neutral compounds in SPC water, red bars for positively charged compounds in SPC water, green bars for neutral compounds in CHCl_3_; **a** MD snapshots; **b** post-optimized MD snapshots; **c** BEST conformers; **d** PMM conformers; **e** CONF conformers

The profiles for the individual compounds in three different media considered are given in Fig. 9 and Additional file 1: Table S2 for conformer ensembles from MD or generator algorithms. As before, the 50,000 snapshots considered are taken from the five MD runs at 300 K with different starting coordinates.

The IMHB population profiles for all molecules based on MD snapshots in Fig. 9a proof the assumption that in CHCl_3_ there is a higher proportion of snapshots with two and even with three IMHB. Unexpectedly, the charged structures in water have a higher proportion of snapshots with zero IMHB, whereas the neutral species have more snapshots with one or two IMHB than the charged species though formally possessing one less hydrogen donor. Visual inspection of the 3D structures reveals that the additional proton disturbs the ring geometry in a way that reduces the interactions of the NH donor at the linker and carbonyl acceptors.

Post-optimizing the snapshots with OPLS3e and implicit solvent increases the proportions of snapshots with higher numbers of IMHBs in most cases (Fig. 9b).

The conformers derived from BEST and CONF shown in Fig. 9c, e, which were also post-optimized with OPLS3e and implicit solvent provide a different picture. For all structures, the IMHB population statistics is zero > one > two >> three. On the other hand, the PMM profiles in Fig. 9d are somehow in between the profiles from not-optimized MD and the other generators.

We conclude that the differences in profiles for PMM (which is using the same force field than the MD) are mostly governed by the influence of the implicit solvent used for post-optimization, whereas the differences for BEST and CONF mostly originate from conformer generation and are additionally influenced by post-optimization. Nevertheless, all generator conformer populations differ from the MD population.

#### Polar surface areas

The chameleonic behaviour seen with regards to IMHB—at least from the MD snapshots—should also be reflected in the distributions of polar surface areas calculated from the 3-dimensional structure.

Obviously, compound **7** has significantly higher 3D-PSA values than the other macrocycles (Table 2). From the IMHB bond profiles we would not expect this behaviour, with 32% and 21% of neutral water snapshots having one or two IMHB, respectively, which in sum is similar or higher compared to the other molecules. Therefore, IMHB statistics cannot be the main source of polar surface area values. Earlier, the group of Lokey had reported side-chains as being more important for the modulation of polar surface areas than IMHB [23, 24]. Visual inspection of the snapshots reveals that the smaller ring size constrains the polar groups to point more to the outside of the molecule.

**Table 2:** Median, minimum and maximum 3D-PSA values in Å^2^ for MD snapshots in different media and charge states

Mol. No	Median 3D-PSA			Minimum 3D-PSA			Maximum 3D-PSA		
	w/n^a^	w/c^b^	c/n^c^	w/n	w/c	c/n	w/n	w/c	c/n
1	98.3	96.9	91.3	66.1	60.8	64.8	123.6	131.4	124.3
2	98.3	98.7	85.5	62.8	65.3	55.4	126.4	131.2	123.3
3	87.0		83.1	58.8		55.1	114.3		116.1
4	83.3	84.6	82.7	53.4	49.1	42.0	117.1	121.5	115.9
5	83.1	85.7	84.6	55.1	50.9	53.7	116.1	117.2	115.2
6	89.9	92.1	80.0	51.5	56.2	51.4	119.0	122.5	117.6
7	115.9	114.4	111.2	88.4	91.7	79.1	138.9	140.8	135.5

^a^Water, neutral

^b^Water, positively charged

^c^Chloroform, neutral

The other driver is as expected *N*-methylation as in compounds **3, 4, 5, 6** which reduces polar surface area by median 8.9 Å^2^ (+−5.6) compared to the highest value in each column.

**Fig. 10:**
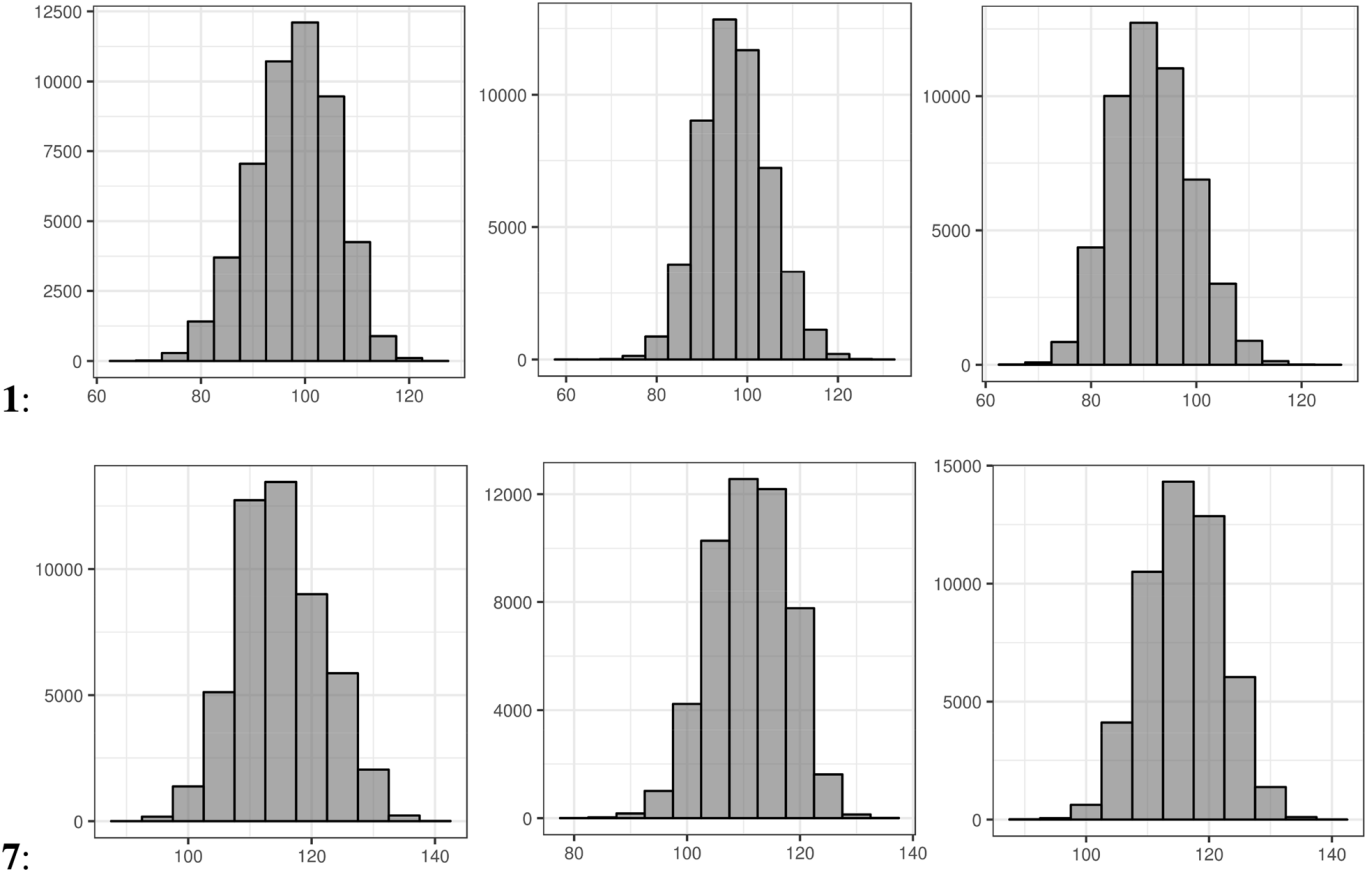
Histograms for the frequencies of 3D polar surface areas in Å^2^ of the conformers derived from five MD simulations at 300 K with five different starting coordinates for compounds **1** (top row) and **7** (bottom row); **a** solvent water, neutral, **b** water, positively charged, **c** CHCl3, neutral

We next looked into the distributions of the 3D-PSA values as exemplified in the histograms shown in Fig. 10 and Additional file 1: Figure S15. Obviously, there is a consistent shift to higher 3D-PSA profiles going from molecule **1** to **7** over the whole time series, but overall, the shapes of the profiles look extremely similar, except maybe for charged 4 in water (cf. Additional file 1: Figure S15), again with a grouping for **1** and **2** as well for **3**, **4**, **5**, **6**. On the other hand, there is not the expected drop going from polar protic water to aprotic unpolar CHCl_3_, and also no significant difference between protonated and neutral form in water.

The changes in 3D-PSA are in fact to a high degree governed by side chain and backbone movements hiding polar functional groups and not so much by stability and changes of the intramolecular hydrogen bond networks.

## Conclusions

The completeness of the conformational ensemble one obtains by a computational method will significantly influence the outcome of any computational study based on the ensemble. In this work we provide a thorough investigation on the multiple parameters that determine the resulting conformer ensembles from molecular dynamics simulations and from three algorithms for the generation of conformers for seven small macrocycles resulting from a collaboration with the University of Sherbrooke.

We show that multiple molecular dynamics simulations on diverse starting conformations per molecule are needed to generate ensembles covering the accessible conformational space, but even such procedure does not guarantee complete sampling, i.e. the intended ensemble completeness, in the case of such highly rigid macrocycles. This is in line with other publications on molecular dynamics and Markov-state modelling.

The conformer map projections from principal components analysis on the ring torsions differ between molecules, for different charge states and for different solvents. Especially the maps for compounds **1** and **2** which are enantiomers (l-Leu vs. d-Leu) differ much more than anticipated, which can be rationalized by the loadings plots from the PCA showing that only a small number of torsions determine the conformer distributions.

The conformer maps in the three solvents considered, namely water, DMSO and CHCl_3_ differ strongly for each molecule and there is low overlap of the densely populated spaces, what, according to current work in the group of Riniker, would be a prerequisite for pre-orientation and smooth transfer through cell-membranes.

Ensembles from molecular dynamics at room temperature cover a conformational space significantly larger than the space of local minima. The maps of post minimized MD derived snapshots span a larger space than maps derived from algorithms for conformer generation. In addition to performing a cluster-based analysis and the evaluation of variance metrics we here propose a novel metric for the quantification of the space spanned by such algorithms compared to MD derived space by applying the Mahalanobis distance used in machine learning as an applicability domain measure and for outlier detection. We show that the space covered by PMM is more complete than the BEST space and that the CONF space is the most restricted one.

Furthermore, we find that whereas the MD ensembles from different explicit solvent simulations look distinct, the implicit solvents used during the post optimization of the raw conformers only slightly influence the final coordinates. Therefore, conformational states in implicit solvents will not reflect the true interactions between solute and solvent and the ensemble obtained from explicit solvent calculations. Any results from such ensembles might be doubted.

Finally, since our investigation aimed at an understanding of parameters influencing membrane permeation as an important parameter in the design of drug candidates, we looked into the two parameters often related to permeation, namely polar surface area and intramolecular hydrogen bonding. For molecules **1** to **6** we see no significant differences in the 3D-PSA profiles over the MD snapshots between the molecules but also for different charge states or solvents for one molecule. Compound **7** with the smaller ring system shows the shift in 3D-PSA, but again there is no differentiation on charge state or solvent. Overall, the molecules are too rigid to react on the exterior. There is no correlation between 3D-PSA and distributions of intramolecular hydrogen bond patterns but at least as expected more IMHB in nonpolar solvents. In contrast, we find that the 3D-PSA of **1** with the smaller ringsize is 20 Å^2^ higher than that of the larger ring **5** but at the same time **1** has a higher mean population of intramolecular hydrogen bonds. Though unintuitive, this can be rationalized by looking at the conformer coordinates. Whereas the small ring is so constrained that it has to expose polar groups to the exterior, the less constrained larger ring can partially mask the polar functionalities by side chains like the aromatic ring of phenylalanine or the leucine chain.

The work performed here is concerned with small rigid macrocycles. Though we expect the findings to be transferable, we will perform a follow-up study on open-chain small drug-like molecules.

## Supplementary Information

**Additional file 1: Table S1.** Data on explained variance by the latent variables PC1 to PC4 from the principal component analyses for the macrocycle ensemble (**1** to **6**: all) and for individual macrocycles for the different charge states. Solvents considered are mixed all solvents, water, DMSO and chloroform. **Table S2.** Percentages of conformers with between 0 and 3 IMHB in different media and charge state. **Table S3.** Cluster populations for compounds **1** to **7** for neutral (n) and charged (c) species in water (W) and chloroform (C).** Table S4. **Results of Homoscedasticity Test with post Hoc Tukey HSD Test. **Figure S1.** Correlation between 2D TPSA and 3D PSA values for 10,000 randomly selected compounds from the Aldrich Market Select catalogue. **Figure S2.** Torsion angle distribution profiles expressed as sinus and cosinus distributions of the original 32-dimensional space after transformation. **Figure S3.** Conformer maps for macrocycles **1** to **6 **in solvent DMSO**,** color-coded by binned raw conformer relative energies with thresholds of 6 and 10 kcal mol^-1^ (conformers with relative energies higher than 100 kcal mol^-1^ were filtered out). **Figure S4. **Conformer maps for macrocycles **1** to **6 **in solvent CHCl_3_**,** color-coded by binned raw conformer relative energies with thresholds of 6 and 10 kcal mol^-1^ (conformers with relative energies higher than 100 kcal mol^-1^ were filtered out). **Figure S5.** Histograms of the energy distributions of the MD snapshots for macrocycles **1** to **7 **in solvent water.** Figure S6.** Conformer ensembles for charged compound **1 **in solvent CHCL_3_.** Figure S7.** Ensembles for compound **2** in water generated by a simulated annealing protocol; a) and c) show the neutral, b) and d) the charged state.** Figure S8.** Density maps for distributions of accumulated conformers derived from five MD simulations at 300 K with five different starting coordinates in solvent water (orange), DMSO (blue), and CHCl_3_ (pink), for neutral compounds **1** to **6**.** Figure S9. **Maps of latent torsional space accessed by post-minimized MD snapshots (5 starting conformers, 300 K 100 ns each) for neutral compounds in SPC water in purple, overlaid with conformers created by BEST (left column), PMM (middle) and CONF (right) in orange for macrocycles **1** to **7** (see row labels).** Figure S10.** Maps of latent torsional space accessed by post-minimized MD snapshots (5 starting conformers, 300 K 100 ns each) for charged compounds **1** to **7** in SPC water in purple, overlaid with conformers created by BEST (left column), PMM (middle) and CONF (right) in orange. Note that compound **3** is missing since it is N-methylated.** Figure S11.** Maps of latent torsional space accessed by post-minimized MD snapshots (5 starting conformers, 300 K 100 ns each) for neutral compounds **1** to **7** in CHCl_3_ in purple, overlaid with conformers created by BEST (left column), PMM (middle) and CONF (right) in orange.** Figure S12.** Maps of latent torsional space accessed by a) raw MD snapshots (5 starting conformers, 300 K 100 ns each, solvent water) for neutral compound **1**, b) raw (non-minimized) conformations by BEST, c) raw conformations by PMM, d) raw conformations by CON.** Figure S13.** 16 plots providing the complete conformer space mapping in 32 latent dimensions for **1**, neutral state in water.** Figure S14. **Dependence of total variance (trace) on the number of principal components considered; a) traces for minimized MD (blue) and BEST (green), both projected on the map created from all raw conformers, and the accumulated variance (cum. Proportion, red), showing that the curves cross at the point of about 90% of explained variance; b) traces for minimized MD (pink) and BEST (blue), with dashed lines for normal distributed noise (mean=0, sd=2*pi/180) generated for each sample for each dihedral angle.** Figure S15.** Histograms of 3D-PSA distributions for the MD snapshots (5 starting conformers, 300 K 100 ns each) of compounds 1 to 7; left column shows neutral compounds in water, center column charged compounds in water, right column neutral compounds in CHCl3. For N-methylated compound three there is no charged species.

**Additional file 2.** Text file with 3D coordinates of starting conformers in SDF file format.

## Data Availability

The following files will be made available free of charge. Additional tables and figures (PDF), 3D coordinates of starting conformers (SDF). The Shiny app with input data for the plots and documentation (shiny_app_and_data.zip) is available from the repository https://github.com/LeaSeep/MC_EnsembleCompletness. All software used is either commercially or under open source licenses available.
