# Supplementary material for: Ensemble completeness in conformer sampling: the case of small macrocycles
Source: J Cheminform. 2021 Jul 29;13:55. doi: 10.1186/s13321-021-00524-0 (PMC8320181; doi:10.1186/s13321-021-00524-0)
Supplement: Supplementary file 1 — Additional file 1: Table S1. Data on explained variance by the latent variables PC1 to PC4 from the principal component analyses for the macrocycle ensemble (1 to 6: all) and for individual macrocycles for the different charge states. Solvents considered are mixed all solvents, water, DMSO and chloroform. Table S2. Percentages of conformers with between 0 and 3 IMHB in different media and charge state. Table S3. Cluster populations for compounds 1 to 7 for neutral (n) and charged (c) species in water (W) and chloroform (C). Table S4. Results of Homoscedasticity Test with post Hoc Tukey HSD Test. Figure S1. Correlation between 2D TPSA and 3D PSA values for 10,000 randomly selected compounds from the Aldrich Market Select catalogue. Figure S2. Torsion angle distribution profiles expressed as sinus and cosinus distributions of the original 32-dimensional space after transformation. Figure S3. Conformer maps for macrocycles 1 to 6 in solvent DMSO, color-coded by binned raw conformer relative energies with thresholds of 6 and 10 kcal mol-1 (conformers with relative energies higher than 100 kcal mol-1 were filtered out). Figure S4. Conformer maps for macrocycles 1 to 6 in solvent CHCl3, color-coded by binned raw conformer relative energies with thresholds of 6 and 10 kcal mol-1 (conformers with relative energies higher than 100 kcal mol-1 were filtered out). Figure S5. Histograms of the energy distributions of the MD snapshots for macrocycles 1 to 7 in solvent water. Figure S6. Conformer ensembles for charged compound 1 in solvent CHCL3. Figure S7. Ensembles for compound 2 in water generated by a simulated annealing protocol; a) and c) show the neutral, b) and d) the charged state. Figure S8. Density maps for distributions of accumulated conformers derived from five MD simulations at 300 K with five different starting coordinates in solvent water (orange), DMSO (blue), and CHCl3 (pink), for neutral compounds 1 to 6. Figure S9. Maps of latent torsional space accessed by po [file 13321_2021_524_MOESM1_ESM.docx]

Additional Material

Ensemble Completeness in Conformer Sampling: Small Macrocycles

*Lea Seep,^1^ Anne Bonin, ^1^ Katharina Meier, ^1^ Holger, Diedam^2^, Andreas H. Göller^1 *^*

^1^Bayer AG, Pharmaceuticals R&D, Digital Technologies, 42096 Wuppertal, Germany

^2^Bayer AG, Engineering & Technology, Applied Mathematics, 51368 Leverkusen, Germany

**Table S1**: Data on explained variance by the latent variables PC1 to PC4 from the principal component analyses for the macrocycle ensemble (**1** to **6**: all) and for individual macrocycles for the different charge states. Solvents considered are mixed all solvents, water, DMSO and chloroform.

| cpd | Solv. | Charge state | PC 1 | PC2 | PC 3 | PC4 |
| --- | --- | --- | --- | --- | --- | --- |
| all | mixed | 0 | 15.1 | 12.6 | 9.9 | 7.6 |
| 1 | mixed | 0 | 33.8 | 14.6 | 11.5 | 6.3 |
|  | mixed | 1 | 26.5 | 19.6 | 12.3 | 10.4 |
| 2 | mixed | 0 | 22.4 | 18.9 | 11.6 | 9.3 |
|  | mixed | 1 | 26.1 | 22.6 | 11.8 | 8.8 |
| 3 | mixed | 0 | 26.5 | 17.1 | 11.7 | 6.3 |
| 4 | mixed | 0 | 27.8 | 16.9 | 11.4 | 7.1 |
|  | mixed | 1 | 20.9 | 17.5 | 11.8 | 8.7 |
| 5 | mixed | 0 | 22.9 | 16.0 | 9.9 | 7.2 |
|  | mixed | 1 | 20.4 | 17.2 | 14.3 | 9.3 |
| 6 | mixed | 0 | 27.1 | 17.1 | 13.0 | 11.5 |
|  | mixed | 1 | 22.9 | 17.7 | 12.6 | 8.4 |
| 7 | mixed | 0 | 47.4 | 23.7 | 8.3 | 6.5 |
|  | mixed | 1 | 62.8 | 14.4 | 9.9 | 4.5 |
| 1 | water | 0 | 32.5 | 14.1 | 9.5 | 6.5 |
|  | DMSO | 0 | 25.1 | 12.9 | 10.5 | 8.9 |
|  | CHCl_3_ | 0 | 35.3 | 19.8 | 10.8 | 6.2 |
|  | water | 1 | 35.8 | 19.6 | 8.6 | 7.4 |
|  | DMSO | 1 | 30.7 | 16.8 | 12.9 | 11.2 |
|  | CHCl_3_ | 1 | 29.6 | 25.2 | 17.4 | 5.8 |

**Table S2:** Percentages of conformers with between 0 and 3 IMHB in different media and charge state. Conformers are derived from molecular dynamics (MD), molecular dynamics with post-optimization (MD/o), or post-optimized conformers from the generator algorithms. The highest populated bin for each medium is reported in bold.

|  |  | Water/neutral | | | | Water/charged | | | | CHCl_3_/neutral | | | |
| --- | --- | --- | --- | --- | --- | --- | --- | --- | --- | --- | --- | --- | --- |
|  |  | 0 | 1 | 2 | 3 | 0 | 1 | 2 | 3 | 0 | 1 | 2 | 3 |
| 1 | MD | 27 | **53** | 19 | 1 | **57** | 37 | 6 | 0 | 6 | 32 | **45** | 16 |
|  | MD/o | 21 | **53** | 26 | 0 | **53** | 23 | 24 | 0 | 1 | 16 | **54** | 29 |
|  | BEST | **62** | 34 | 4 | 0 | **58** | 36 | 5 | 0 | 38 | **44** | 15 | 4 |
|  | PMM | 13 | **49** | 29 | 8 | 37 | **39** | 24 | 1 | 10 | 38 | **42** | 10 |
|  | CONF | **67** | 32 | 1 | 0 | **82** | 15 | 3 | 0 | **59** | 34 | 8 | 0 |
| 2 | MD | **50** | 41 | 8 | 1 | **65** | 31 | 4 | 0 | 8 | 31 | **43** | 19 |
|  | MD/o | **48** | 38 | 13 | 1 | **41** | 35 | 24 | 0 | 3 | 17 | **41** | 40 |
|  | BEST | **62** | 34 | 4 | 0 | **58** | 36 | 5 | 0 | 38 | **44** | 15 | 4 |
|  | PMM | 20 | **45** | 28 | 7 | 26 | **43** | 28 | 3 | 10 | **38** | 37 | 15 |
|  | CONF | **57** | 29 | 15 | 0 | **80** | 18 | 1 | 0 | **40** | 35 | 17 | 9 |
| 3 | MD | **50** | 39 | 9 | 1 |  |  |  |  | 16 | **50** | 27 | 6 |
|  | MD/o | **57** | 30 | 12 | 1 |  |  |  |  | 1 | **60** | 28 | 10 |
|  | BEST | **74** | 22 | 4 | 0 |  |  |  |  | **48** | 37 | 13 | 2 |
|  | PMM | 41 | **44** | 15 | 0 |  |  |  |  | 25 | **45** | 28 | 1 |
|  | CONF | **87** | 13 | 0 | 0 |  |  |  |  | **71** | 28 | 1 | 0 |
| 4 | MD | 43 | **53** | 4 | 0 | **77** | 22 | 2 | 0 | 34 | **53** | 13 | 1 |
|  | MD/o | 32 | **64** | 4 | 0 | **65** | 32 | 3 | 0 | 15 | **75** | 10 | 0 |
|  | BEST | **66** | 32 | 2 | 0 | **49** | 46 | 4 | 0 | **52** | 42 | 6 | 0 |
|  | PMM | **56** | 41 | 3 | 1 | 42 | **45** | 12 | 0 | 44 | **49** | 7 | 1 |
|  | CONF | **63** | 37 | 1 | 0 | **73** | 22 | 5 | 0 | 45 | **54** | 1 | 0 |
| 5 | MD | **59** | 35 | 5 | 0 | **74** | 22 | 4 | 0 | 15 | **58** | 27 | 1 |
|  | MD/o | **65** | 28 | 7 | 0 | **65** | 28 | 7 | 0 | 1 | **54** | 45 | 0 |
|  | BEST | **64** | 32 | 4 | 0 | **55** | 38 | 7 | 0 | 36 | **51** | 12 | 1 |
|  | PMM | **50** | 41 | 8 | 0 | 39 | **47** | 14 | 0 | 38 | **47** | 15 | 0 |
|  | CONF | **65** | 33 | 2 | 0 | **68** | 26 | 6 | 0 | **46** | **46** | 7 | 0 |
| 6 | MD | **54** | 40 | 6 | 0 | **73** | 24 | 2 | 0 | 10 | **67** | 20 | 3 |
|  | MD/o | **48** | 46 | 6 | 0 | **48** | 46 | 6 | 0 | 4 | **66** | 27 | 3 |
|  | BEST | **66** | 32 | 2 | 0 | **54** | 40 | 6 | 0 | **43** | 40 | 16 | 0 |
|  | PMM | 38 | **54** | 8 | 0 | 33 | **52** | 15 | 0 | 25 | **56** | 18 | 0 |
|  | CONF | **81** | 19 | 1 | 0 | **68** | 30 | 2 | 0 | **63** | 32 | 5 | 0 |
| 7 | MD | **46** | 32 | 21 | 1 | **96** | 3 | 1 | 0 | 12 | 29 | **52** | 7 |
|  | MD/o | 32 | **42** | 25 | 1 | 32 | **42** | 25 | 1 | 14 | 15 | **59** | 12 |
|  | BEST | **48** | 33 | 18 | 2 | **46** | 44 | 10 | 0 | **38** | **38** | 20 | 4 |
|  | PMM | 10 | **55** | 27 | 8 | 16 | **55** | 20 | 10 | 5 | **53** | 25 | 17 |
|  | CONF | **51** | 46 | 3 | 0 | **73** | 22 | 6 | 0 | **48** | 43 | 9 | 0 |

**Table S3:** Cluster populations for compounds **1** to **7** for neutral (n) and charged (c) species in water (W) and chloroform (C). K-means clustering is based on the first 8 principal components (from sin and cos of the 16 dihedral angles defining compounds 1 to 6) of the combined post-optimized conformers from the MD simulation and the respective generator (GEN). Based on experiments and visual inspection of elbow plots (not shown; RMSD vs numbers of clusters) we decided on 500 clusters, resulting in 100 conformations on average per cluster. k-means creates quite homogenous clusters with a median cluster size of 87.6 ± 6.1 and a mean cluster size of 100.3 ± 0.1. The median RMSD over all clusters of each method is quite low, but there are clusters that are not so homogenous, but still have a low maximum RMSD. With exception of the charged state of the smaller and more rigid macrocycle **7**, we find significantly less mixed clusters for CONF than for BEST and PMM. There is no obvious pattern for the numbers of unique GEN clusters. But for any clustering there are about ten times more unique MD clusters than mixed and unique GEN clusters, quantifying the observations from Figure 6 (based on 2 PCs only) on the uncovered conformer space and demonstrating that there is space not covered by MD

Reported are the numbers of unique clusters (structures from one approach only), the mixed clusters and singletons, the median and maximum RMSD over all clusters, the numbers of conformers in the GEN unique clusters and the median cluster size.

| **Conf** | **Solv** | **CS** | **unique GEN** | **unique MD** | **mixed clusters** | **# single tons** | **max RMSD** | **median RMSD** | **# conf. unique GEN** | **median Cluster Size** |  |
| --- | --- | --- | --- | --- | --- | --- | --- | --- | --- | --- | --- |
| **1** | | | | | | | | | | |  |
| Conf | W | n | 1 | 463 | 36 | 1 | 1.5687 | 0.0037 | 13 | 80 |  |
| BEST | W | n | 7 | 429 | 64 | 1 | 1.6876 | 0.0044 | 51 | 83 |  |
| Prime | W | n | 4 | 406 | 90 | 1 | 1.3301 | 0.0032 | 7 | 82 |  |
| Conf | C | n | 8 | 468 | 24 | 2 | 1.5402 | 0.0048 | 75 | 77 |  |
| BEST | C | n | 9 | 438 | 53 | 3 | 1.6268 | 0.0045 | 91 | 80 |  |
| Prime | C | n | 10 | 397 | 93 | 3 | 1.3407 | 0.0054 | 41 | 72 |  |
| Conf | W | c | 4 | 476 | 20 | 2 | 1.6677 | 0.0012 | 66 | 89 |  |
| BEST | W | c | 15 | 426 | 59 | 4 | 1.5928 | 0.0014 | 84 | 83 |  |
| Prime | W | c | 9 | 418 | 73 | 2 | 1.3421 | 0.0013 | 51 | 88 |  |
| Conf | C | c | 10 | 480 | 10 | 3 | 1.5146 | 0.0011 | 111 | 91 |  |
| BEST | C | c | 20 | 438 | 42 | 3 | 1.6174 | 0.0011 | 122 | 89 |  |
| Prime | C | c | 16 | 432 | 52 | 3 | 1.3765 | 0.0011 | 82 | 90 |  |
| **2** | | | | | | | | | | |  |
| Conf | W | n | 3 | 457 | 40 | 3 | 1.5614 | 0.0030 | 18 | 73 |  |
| BEST | W | n | 11 | 445 | 44 | 0 | 1.6875 | 0.0031 | 107 | 78 |  |
| Prime | W | n | 7 | 416 | 77 | 0 | 1.3903 | 0.0030 | 54 | 77 |  |
| Conf | C | n | 6 | 459 | 35 | 3 | 1.6524 | 0.0025 | 65 | 79 |  |
| BEST | C | n | 13 | 451 | 36 | 2 | 1.6678 | 0.0033 | 116 | 79 |  |
| Prime | C | n | 7 | 421 | 72 | 3 | 1.3937 | 0.0022 | 47 | 82 |  |
| Conf | W | c | 12 | 466 | 22 | 2 | 1.4794 | 0.0016 | 73 | 80 |  |
| BEST | W | c | 15 | 438 | 47 | 1 | 1.6647 | 0.0017 | 121 | 79 |  |
| Prime | W | c | 23 | 420 | 57 | 5 | 1.3840 | 0.0017 | 98 | 81 |  |
| Conf | C | c | 9 | 470 | 21 | 2 | 1.4685 | 0.0014 | 66 | 84 |  |
| BEST | C | c | 29 | 441 | 30 | 2 | 1.6341 | 0.0016 | 141 | 79 |  |
| Prime | C | c | 21 | 424 | 55 | 3 | 1.3379 | 0.0016 | 78 | 84 |  |
| **3** | | | | | | | | | | |  |
| Conf | W | n | 10 | 462 | 28 | 15 | 1.4956 | 0.0062 | 37 | 61 |  |
| BEST | W | n | 13 | 394 | 93 | 2 | 1.6161 | 0.0049 | 51 | 71 |  |
| Prime | W | n | 5 | 396 | 99 | 4 | 1.3088 | 0.0047 | 10 | 72 |  |
| Conf | C | n | 5 | 465 | 30 | 2 | 1.5376 | 0.0044 | 70 | 80 |  |
| BEST | C | n | 16 | 422 | 62 | 3 | 1.6680 | 0.0049 | 101 | 76 |  |
| Prime | C | n | 9 | 404 | 87 | 4 | 1.4094 | 0.0044 | 40 | 76 |  |
| **4** | | | | | | | | | | |  |
| Conf | W | n | 4 | 464 | 32 | 2 | 1.4532 | 0.0030 | 32 | 73 |  |
| BEST | W | n | 13 | 401 | 86 | 1 | 1.5695 | 0.0029 | 57 | 77 |  |
| Prime | W | n | 10 | 398 | 92 | 1 | 1.3666 | 0.0036 | 35 | 79 |  |
| Conf | C | n | 5 | 455 | 40 | 1 | 1.5682 | 0.0060 | 30 | 78 |  |
| BEST | C | n | 18 | 419 | 63 | 0 | 1.5919 | 0.0078 | 92 | 74 |  |
| Prime | C | n | 11 | 415 | 74 | 1 | 1.5594 | 0.0077 | 53 | 80 |  |
| Conf | W | c | 10 | 472 | 18 | 1 | 1.4969 | 0.0010 | 91 | 83 |  |
| BEST | W | c | 21 | 433 | 46 | 0 | 1.5260 | 0.0013 | 102 | 84 |  |
| Prime | W | c | 11 | 420 | 69 | 2 | 1.4212 | 0.0011 | 58 | 83 |  |
| Conf | C | c | 15 | 453 | 32 | 5 | 1.4682 | 0.0015 | 85 | 85 |  |
| BEST | C | c | 26 | 436 | 38 | 4 | 1.5837 | 0.0017 | 114 | 84 |  |
| Prime | C | c | 23 | 415 | 62 | 2 | 1.5015 | 0.0017 | 87 | 87 |  |
| **5** | | | | | | | | | | |  |
| Conf | W | n | 6 | 453 | 41 | 1 | 1.4169 | 0.0023 | 42 | 81 |  |
| BEST | W | n | 21 | 412 | 67 | 1 | 1.5660 | 0.0028 | 95 | 80 |  |
| Prime | W | n | 16 | 402 | 82 | 2 | 1.5132 | 0.0028 | 69 | 79 |  |
| Conf | C | n | 10 | 470 | 20 | 5 | 1.4283 | 0.0024 | 90 | 62 |  |
| BEST | C | n | 45 | 423 | 32 | 6 | 1.6123 | 0.0023 | 155 | 62 |  |
| Prime | C | n | 36 | 425 | 39 | 7 | 1.5006 | 0.0026 | 135 | 58 |  |
| Conf | W | c | 12 | 461 | 27 | 6 | 1.5210 | 0.0013 | 99 | 81 |  |
| BEST | W | c | 22 | 430 | 48 | 0 | 1.6364 | 0.0013 | 112 | 79 |  |
| Prime | W | c | 20 | 427 | 53 | 2 | 1.4346 | 0.0013 | 90 | 77 |  |
| Conf | C | c | 12 | 454 | 34 | 3 | 1.4262 | 0.0016 | 89 | 84 |  |
| BEST | C | c | 26 | 427 | 47 | 4 | 1.6057 | 0.0018 | 134 | 85 |  |
| Prime | C | c | 26 | 423 | 51 | 6 | 1.4237 | 0.0016 | 106 | 82 |  |
| **6** | | | | | | | | | | |  |
| Conf | W | n | 8 | 466 | 26 | 1 | 1.5290 | 0.0022 | 100 | 80 |  |
| BEST | W | n | 12 | 433 | 55 | 0 | 1.6153 | 0.0024 | 86 | 78 |  |
| Prime | W | n | 12 | 415 | 73 | 0 | 1.4536 | 0.0025 | 76 | 74 |  |
| Conf | C | n | 6 | 467 | 27 | 0 | 1.4946 | 0.0021 | 73 | 90 |  |
| BEST | C | n | 6 | 440 | 54 | 1 | 1.6025 | 0.0020 | 57 | 90 |  |
| Prime | C | n | 8 | 423 | 69 | 1 | 1.6103 | 0.0021 | 52 | 90 |  |
| Conf | W | c | 5 | 460 | 35 | 1 | 1.5516 | 0.0018 | 51 | 84 |  |
| BEST | W | c | 20 | 412 | 68 | 1 | 1.6421 | 0.0024 | 104 | 79 | |
| Prime | W | c | 13 | 408 | 79 | 1 | 1.4215 | 0.0019 | 61 | 83.5 | |
| Conf | C | c | 8 | 463 | 29 | 4 | 1.4635 | 0.0016 | 71 | 88.5 | |
| BEST | C | c | 25 | 431 | 44 | 0 | 1.6315 | 0.0017 | 125 | 86 | |
| Prime | C | c | 22 | 413 | 65 | 4 | 1.3856 | 0.0017 | 80 | 84.5 | |
| **7** | | | | | | | | | | | |
| Conf | W | n | 17 | 470 | 13 | 4 | 1.3274 | 0.0008 | 158 | 92 | |
| BEST | W | n | 27 | 444 | 29 | 1 | 1.3820 | 0.0008 | 135 | 91 | |
| Prime | W | n | 6 | 470 | 24 | 6 | 0.9258 | 0.0008 | 13 | 89.5 | |
| Conf | C | n | 14 | 470 | 16 | 1 | 1.3402 | 0.0009 | 152 | 88 | |
| BEST | C | n | 32 | 435 | 33 | 6 | 1.3486 | 0.0009 | 139 | 86.5 | |
| Prime | C | n | 3 | 466 | 31 | 1 | 0.7560 | 0.0009 | 14 | 86.5 | |
| Conf | W | c | 24 | 459 | 17 | 2 | 1.1914 | 0.0005 | 152 | 94 | |
| BEST | W | c | 32 | 453 | 15 | 3 | 1.3685 | 0.0005 | 160 | 93 | |
| Prime | W | c | 7 | 476 | 17 | 4 | 0.9104 | 0.0005 | 14 | 96 | |
| Conf | C | c | 19 | 468 | 13 | 5 | 1.4123 | 0.0007 | 153 | 94 | |
| BEST | C | c | 29 | 458 | 13 | 3 | 1.3785 | 0.0007 | 170 | 94 | |
| Prime | C | c | 7 | 478 | 15 | 3 | 0.9303 | 0.0007 | 17 | 93 | |

**Table S4.** Results of Homoscedasticity Test with post Hoc Tukey HSD Test. To test whether energy-minimized conformations sampled by either MD or a Conformation-Generator differ with respect to their variability, the multivariate distributions where tested using the function *betadisper* from the R package *vegan*. Post Hoc Tukey HSD were conducted to quantify a significant difference, $\Delta D$, describing the difference between the mean distance-to-centroid of respective group: $\Delta D=D_{miniMD}-D_{generator}$ (note for non-significant ‘pvalBetadisper’ the Tukey HSD results are expected to be non-significant). Results are shown for the plots within the main manuscript.

| cpd | Method vs miniMD | solvent | charge | pVal | Tukey HSD | | | |
| --- | --- | --- | --- | --- | --- | --- | --- | --- |
|  |  |  |  |  | $\Delta D$ | lower | upper | pVal(adj) |
| 1 | BEST | Water | charged | 1.00E-04 | 8.27E-01 | 7.45E-01 | 9.10E-01 | 0.00E+00 |
| 1 | BEST | CHCL3 | neutral | 1.00E-04 | 7.32E-01 | 6.67E-01 | 7.97E-01 | 0.00E+00 |
| 1 | BEST | Water | neutral | 1.00E-04 | 7.14E-01 | 6.31E-01 | 7.98E-01 | 0.00E+00 |
| 1 | Conformator | Water | charged | 1.00E-04 | 6.12E-01 | 5.15E-01 | 7.09E-01 | 0.00E+00 |
| 1 | Conformator | CHCL3 | neutral | 1.00E-04 | 5.38E-01 | 4.67E-01 | 6.10E-01 | 0.00E+00 |
| 1 | Conformator | Water | neutral | 1.00E-04 | 5.13E-01 | 4.22E-01 | 6.05E-01 | 1.64E-14 |
| 1 | PMM | Water | charged | 1.00E-04 | 4.98E-01 | 4.13E-01 | 5.82E-01 | 0.00E+00 |
| 1 | PMM | CHCL3 | neutral | 1.00E-04 | 3.92E-01 | 3.25E-01 | 4.60E-01 | 0.00E+00 |
| 1 | PMM | Water | neutral | 1.00E-04 | 3.58E-01 | 2.72E-01 | 4.45E-01 | 2.51E-14 |
| 2 | BEST | Water | charged | 1.00E-04 | 7.24E-01 | 6.82E-01 | 7.66E-01 | 0.00E+00 |
| 2 | BEST | CHCL3 | neutral | 1.00E-04 | 1.38E+00 | 1.17E+00 | 1.58E+00 | 0.00E+00 |
| 2 | BEST | Water | neutral | 1.00E-04 | 6.53E-01 | 6.02E-01 | 7.03E-01 | 0.00E+00 |
| 2 | Conformator | Water | charged | 1.00E-04 | 5.70E-01 | 5.20E-01 | 6.20E-01 | 0.00E+00 |
| 2 | Conformator | CHCL3 | neutral | 1.00E-04 | 1.07E+00 | 8.43E-01 | 1.30E+00 | 2.22E-14 |
| 2 | Conformator | Water | neutral | 1.00E-04 | 3.53E-01 | 2.97E-01 | 4.08E-01 | 0.00E+00 |
| 2 | PMM | Water | charged | 1.00E-04 | 4.23E-01 | 3.79E-01 | 4.66E-01 | 0.00E+00 |
| 2 | PMM | CHCL3 | neutral | 1.00E-04 | 1.09E+00 | 8.86E-01 | 1.30E+00 | 1.77E-14 |
| 2 | PMM | Water | neutral | 1.00E-04 | 3.57E-01 | 3.06E-01 | 4.07E-01 | 0.00E+00 |
| 3 | BEST | CHCL3 | neutral | 1.00E-04 | 6.96E-01 | 6.50E-01 | 7.42E-01 | 0.00E+00 |
| 3 | BEST | Water | neutral | 1.00E-04 | 6.02E-01 | 5.35E-01 | 6.69E-01 | 0.00E+00 |
| 3 | Conformator | CHCL3 | neutral | 1.00E-04 | 4.52E-01 | 4.00E-01 | 5.04E-01 | 0.00E+00 |
| 3 | Conformator | Water | neutral | 1.00E-04 | 3.50E-01 | 2.73E-01 | 4.26E-01 | 1.35E-14 |
| 3 | PMM | CHCL3 | neutral | 1.00E-04 | 3.33E-01 | 2.87E-01 | 3.79E-01 | 0.00E+00 |
| 3 | PMM | Water | neutral | 1.00E-04 | 2.37E-01 | 1.69E-01 | 3.05E-01 | 6.18E-12 |
| 4 | BEST | Water | charged | 1.00E-04 | 4.53E-01 | 3.85E-01 | 5.22E-01 | 0.00E+00 |
| 4 | BEST | CHCL3 | neutral | 1.00E-04 | 5.37E-01 | 4.96E-01 | 5.78E-01 | 0.00E+00 |
| 4 | BEST | Water | neutral | 1.00E-04 | 4.86E-01 | 4.12E-01 | 5.61E-01 | 0.00E+00 |
| 4 | Conformator | Water | charged | 1.00E-04 | 1.76E-01 | 1.05E-01 | 2.46E-01 | 9.18E-07 |
| 4 | Conformator | CHCL3 | neutral | 1.00E-04 | 1.86E-01 | 1.40E-01 | 2.32E-01 | 2.84E-14 |
| 4 | Conformator | Water | neutral | 5.00E-04 | 1.44E-01 | 6.17E-02 | 2.27E-01 | 6.12E-04 |
| 4 | PMM | Water | charged | 1.00E-04 | 2.74E-01 | 2.04E-01 | 3.43E-01 | 2.60E-14 |
| 4 | PMM | CHCL3 | neutral | 1.00E-04 | 3.13E-01 | 2.72E-01 | 3.55E-01 | 0.00E+00 |
| 4 | PMM | Water | neutral | 1.00E-04 | 2.62E-01 | 1.87E-01 | 3.37E-01 | 7.21E-12 |
| 5 | BEST | Water | charged | 1.00E-04 | 4.86E-01 | 4.33E-01 | 5.38E-01 | 0.00E+00 |
| 5 | BEST | CHCL3 | neutral | 1.00E-04 | 9.48E-01 | 8.80E-01 | 1.02E+00 | 0.00E+00 |
| 5 | BEST | Water | neutral | 1.00E-04 | 3.45E-01 | 3.06E-01 | 3.83E-01 | 0.00E+00 |
| 5 | Conformator | Water | charged | 2.00E-04 | 1.10E-01 | 5.29E-02 | 1.66E-01 | 1.51E-04 |
| 5 | Conformator | CHCL3 | neutral | 1.00E-04 | 6.79E-01 | 5.96E-01 | 7.62E-01 | 0.00E+00 |
| 5 | Conformator | Water | neutral | 1.65E-01 | 3.28E-02 | -1.34E-02 | 7.91E-02 | 1.64E-01 |
| 5 | PMM | Water | charged | 1.00E-04 | 3.12E-01 | 2.59E-01 | 3.66E-01 | 0.00E+00 |
| 5 | PMM | CHCL3 | neutral | 1.00E-04 | 7.98E-01 | 7.30E-01 | 8.67E-01 | 0.00E+00 |
| 5 | PMM | Water | neutral | 1.00E-04 | 1.77E-01 | 1.39E-01 | 2.15E-01 | 2.49E-14 |
| 6 | BEST | Water | charged | 1.00E-04 | 5.77E-01 | 5.20E-01 | 6.34E-01 | 0.00E+00 |
| 6 | BEST | CHCL3 | neutral | 1.00E-04 | 1.06E+00 | 8.13E-01 | 1.30E+00 | 1.95E-14 |
| 6 | BEST | Water | neutral | 1.00E-04 | 4.86E-01 | 4.46E-01 | 5.26E-01 | 0.00E+00 |
| 6 | Conformator | Water | charged | 1.00E-04 | 3.07E-01 | 2.49E-01 | 3.64E-01 | 2.12E-14 |
| 6 | Conformator | CHCL3 | neutral | 1.00E-04 | 8.86E-01 | 6.38E-01 | 1.13E+00 | 2.39E-12 |
| 6 | Conformator | Water | neutral | 1.00E-04 | 3.26E-01 | 2.85E-01 | 3.66E-01 | 0.00E+00 |
| 6 | PMM | Water | charged | 1.00E-04 | 3.94E-01 | 3.36E-01 | 4.52E-01 | 0.00E+00 |
| 6 | PMM | CHCL3 | neutral | 1.00E-04 | 8.46E-01 | 5.98E-01 | 1.09E+00 | 2.35E-11 |
| 6 | PMM | Water | neutral | 1.00E-04 | 2.83E-01 | 2.42E-01 | 3.23E-01 | 0.00E+00 |
| 7 | BEST | Water | charged | 1.00E-04 | 6.43E-01 | 4.47E-01 | 8.39E-01 | 1.31E-10 |
| 7 | BEST | CHCL3 | neutral | 1.00E-04 | 6.93E-01 | 5.45E-01 | 8.41E-01 | 2.39E-14 |
| 7 | BEST | Water | neutral | 1.00E-04 | 7.30E-01 | 5.94E-01 | 8.65E-01 | 1.85E-14 |
| 7 | Conformator | Water | charged | 2.00E-04 | 4.53E-01 | 2.46E-01 | 6.60E-01 | 1.73E-05 |
| 7 | Conformator | CHCL3 | neutral | 1.00E-04 | 4.57E-01 | 3.01E-01 | 6.13E-01 | 9.30E-09 |
| 7 | Conformator | Water | neutral | 1.00E-04 | 4.94E-01 | 3.51E-01 | 6.36E-01 | 1.12E-11 |
| 7 | PMM | Water | charged | 8.37E-01 | 4.14E-02 | -3.47E-01 | 4.30E-01 | 8.34E-01 |
| 7 | PMM | CHCL3 | neutral | 3.55E-01 | 1.30E-01 | -1.41E-01 | 4.01E-01 | 3.48E-01 |
| 7 | PMM | Water | neutral | 2.53E-01 | 1.45E-01 | -1.03E-01 | 3.92E-01 | 2.52E-01 |


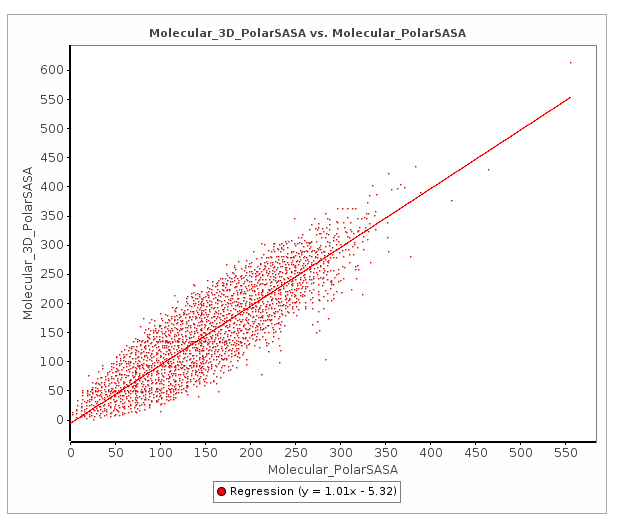


**Figure S1** Correlation between 2D TPSA and 3D PSA values for 10,000 randomly selected compounds from the Aldrich Market Select catalogue


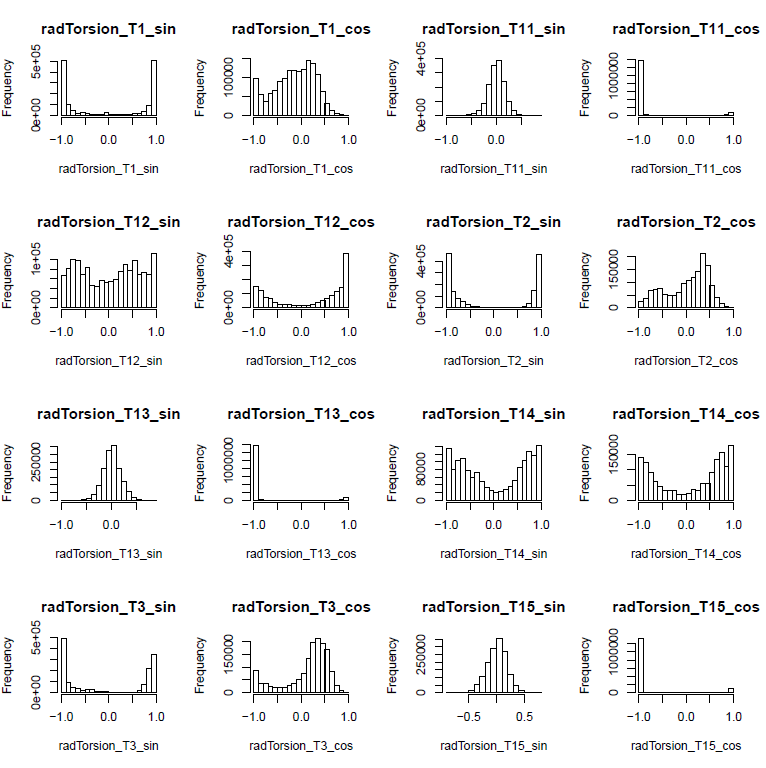


**Figure S2:** Torsion angle distribution profiles expressed as sinus and cosinus distributions of the original 32-dimensional space after transformation. The values are derived from the global map spanned by all MD snapshots (300K, 100 ns, 5 diverse starting conformers) of neutral and positivley charged compounds **1** to **6** in three solvents.


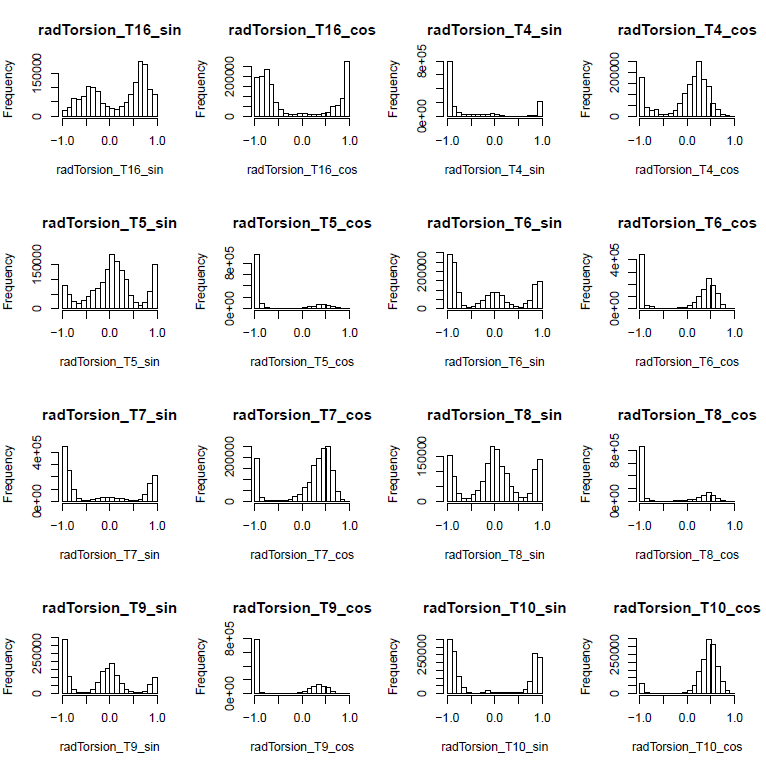


Figure S2, continued.


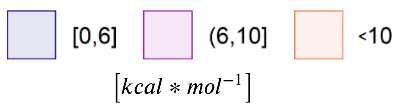
a)
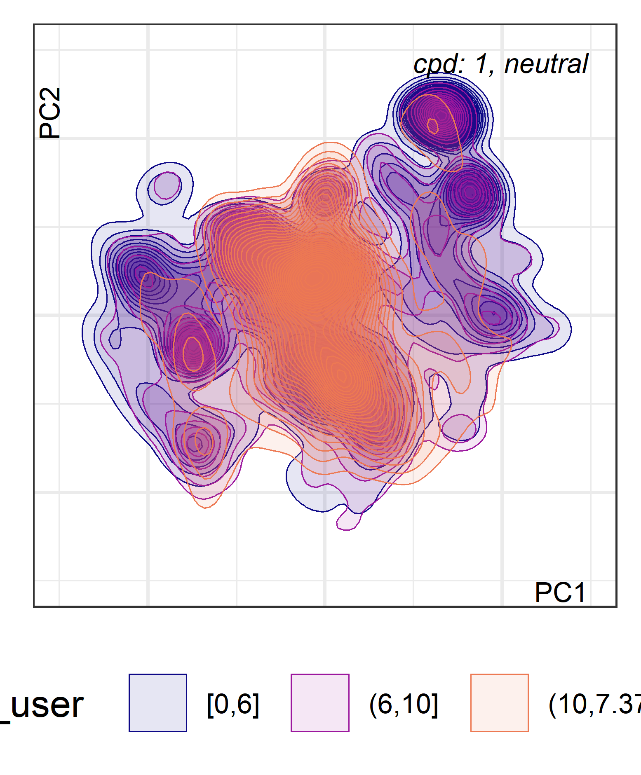
b)
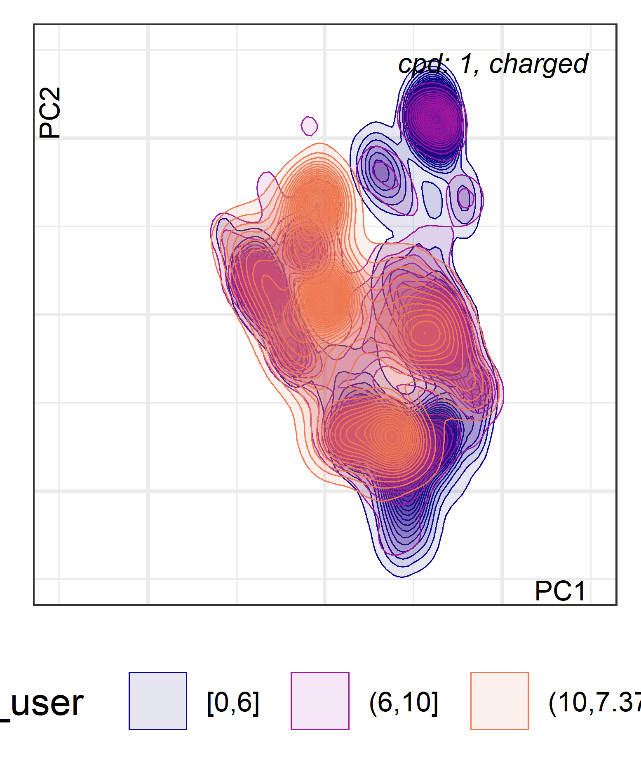


c)
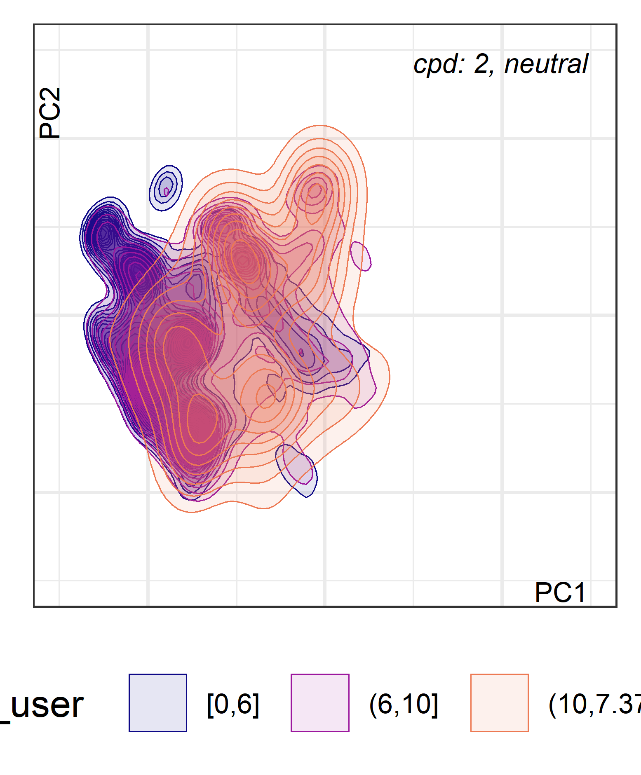
d)
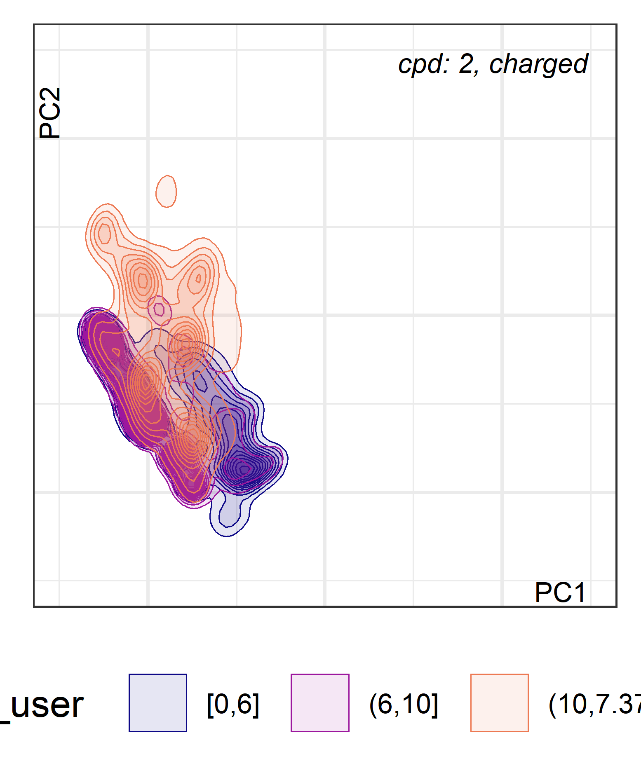


e)
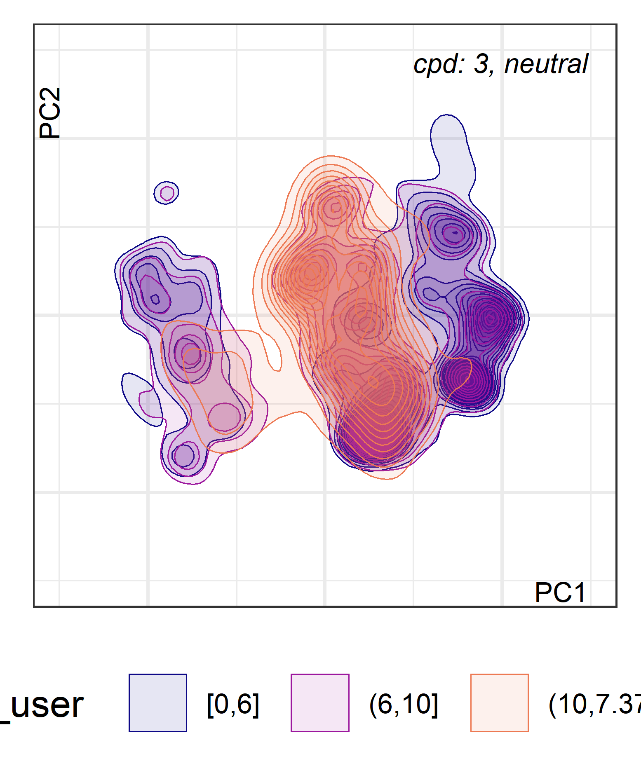
 f)
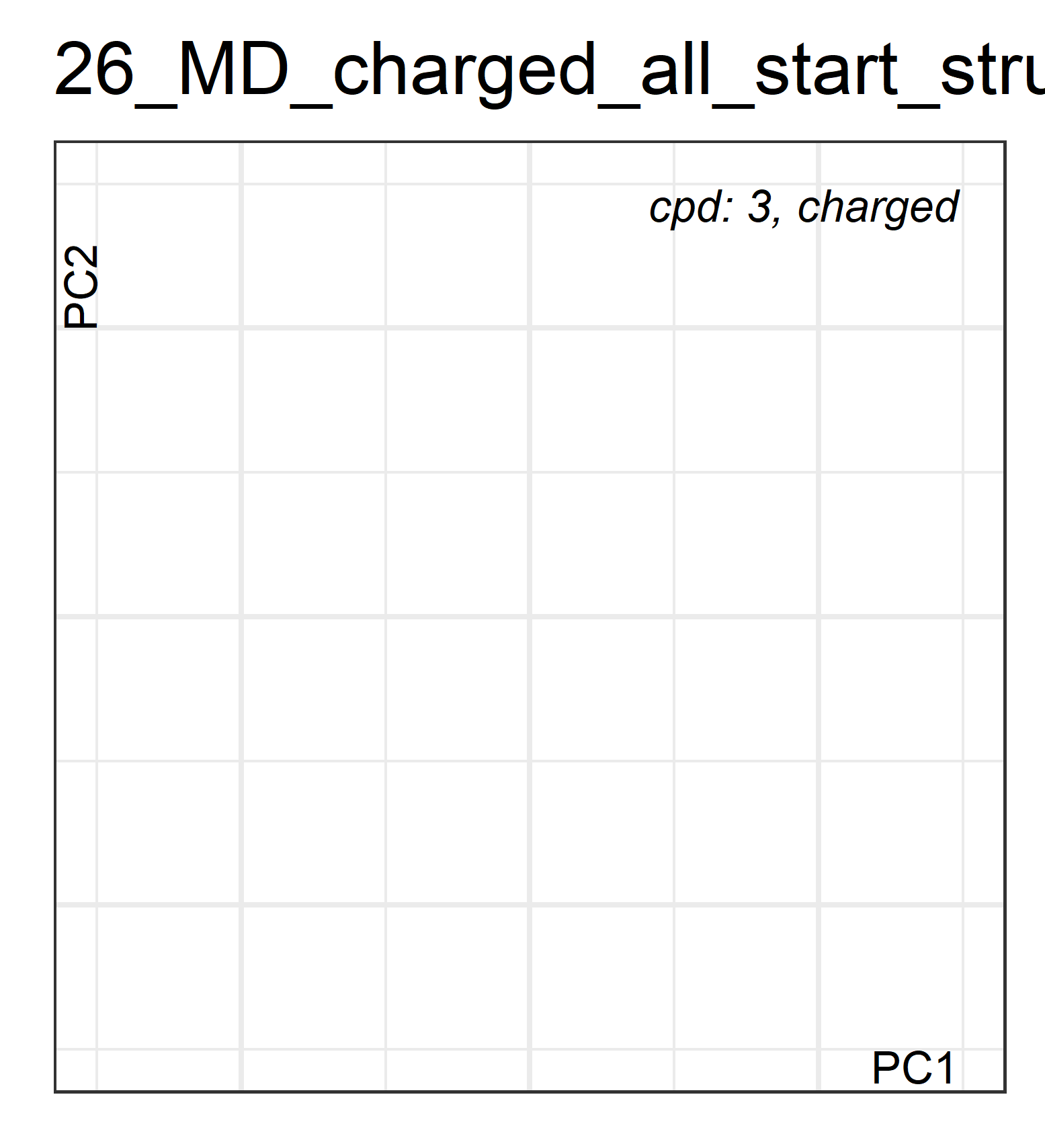


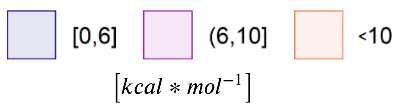
g)
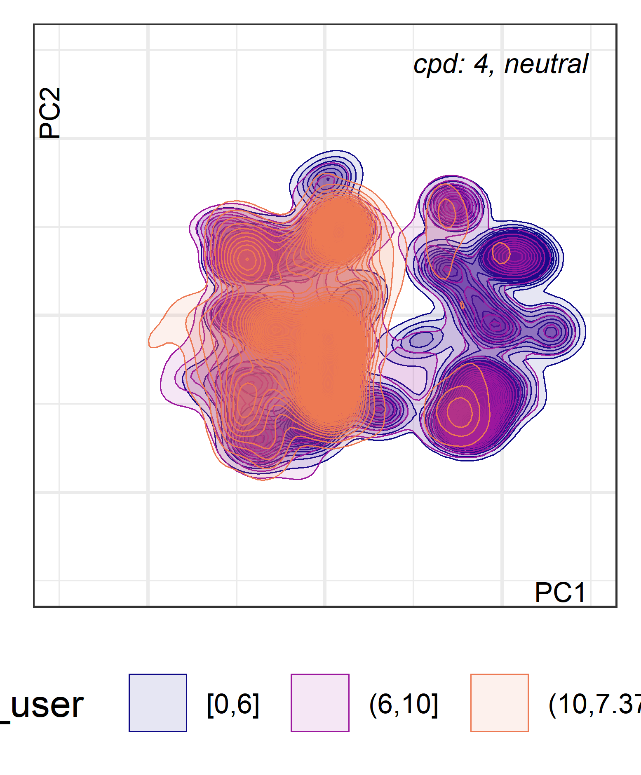
h)
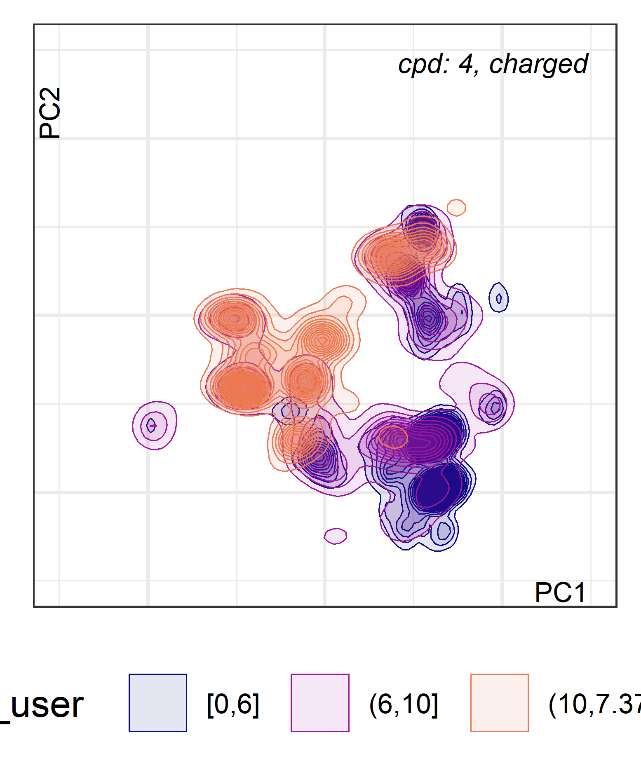


i)
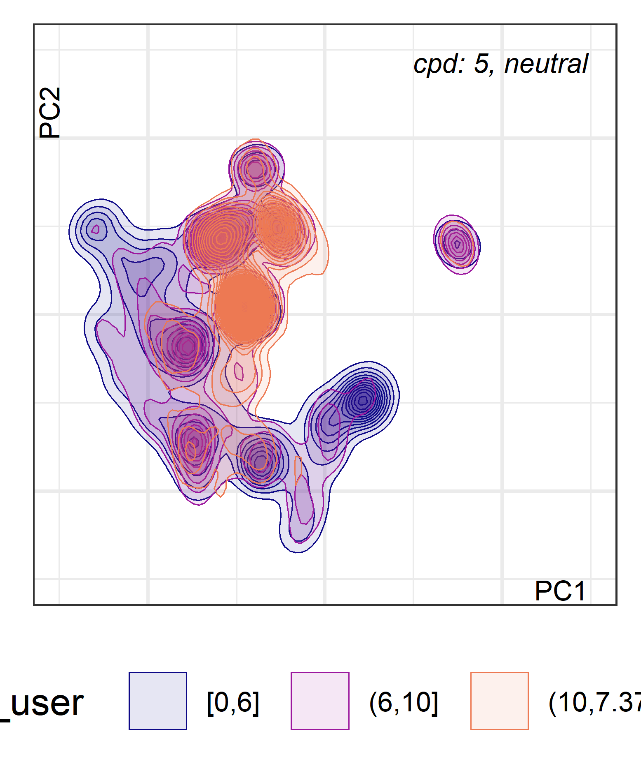
j)
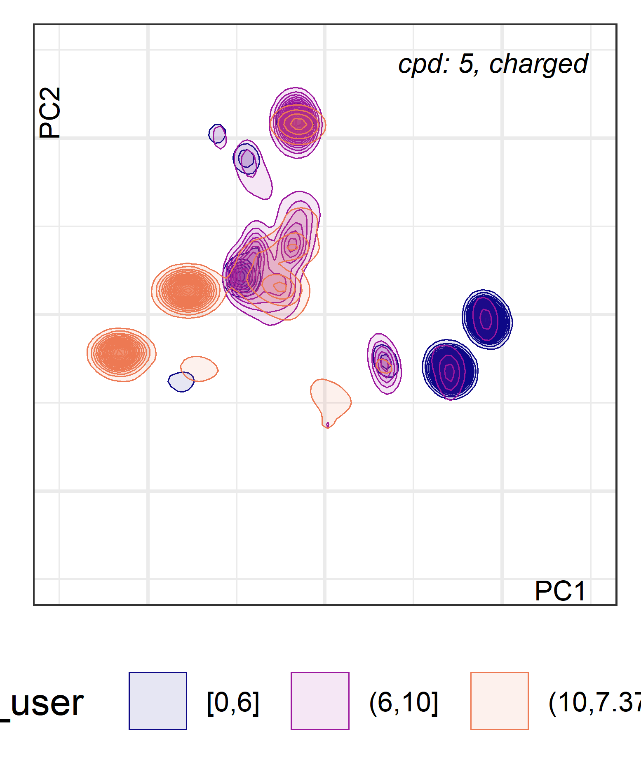


k)
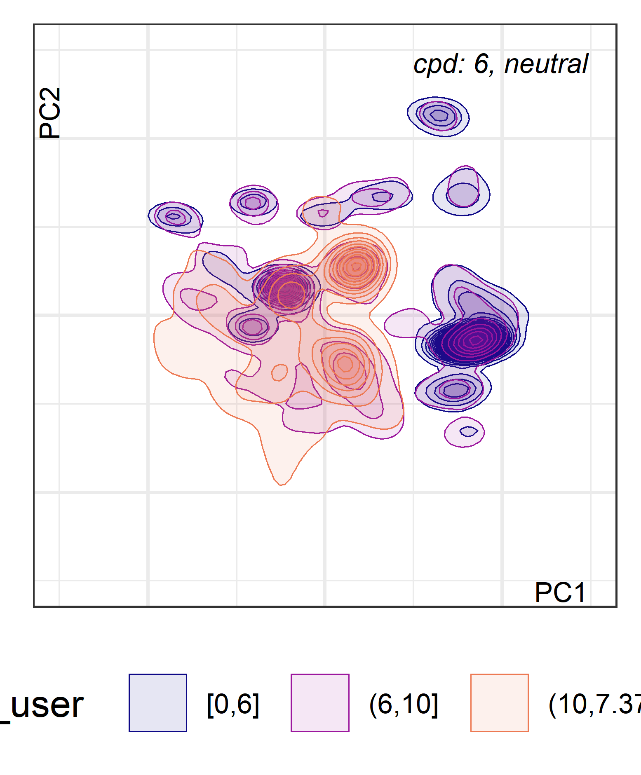
 l)
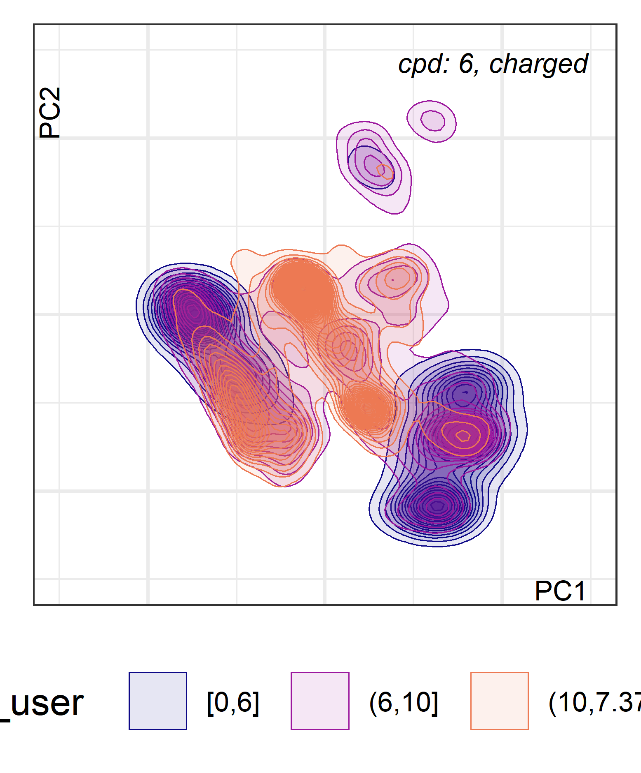


**Figure S3**: Conformer maps for macrocycles **1** to **6** in solvent DMSO**,** color-coded by binned raw conformer relative energies with thresholds of 6 and 10 kcal mol^-1^ (conformers with relative energies higher than 100 kcal mol^-1^ were filtered out). Plots a), c), e), g), i), k) show maps for neutral and plots b), d), h), j), l) for positively charged ligands (ligand **3** is N-methylated, therefore not charged).

a)
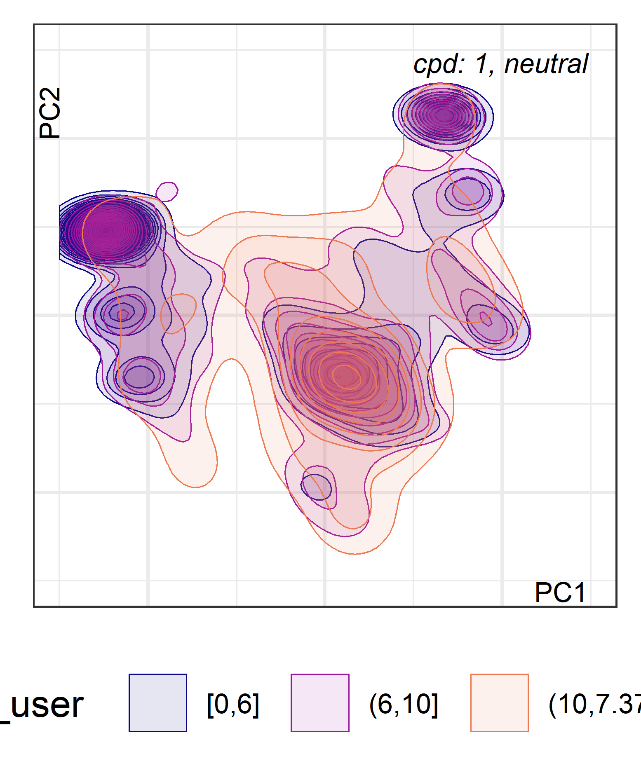
b)
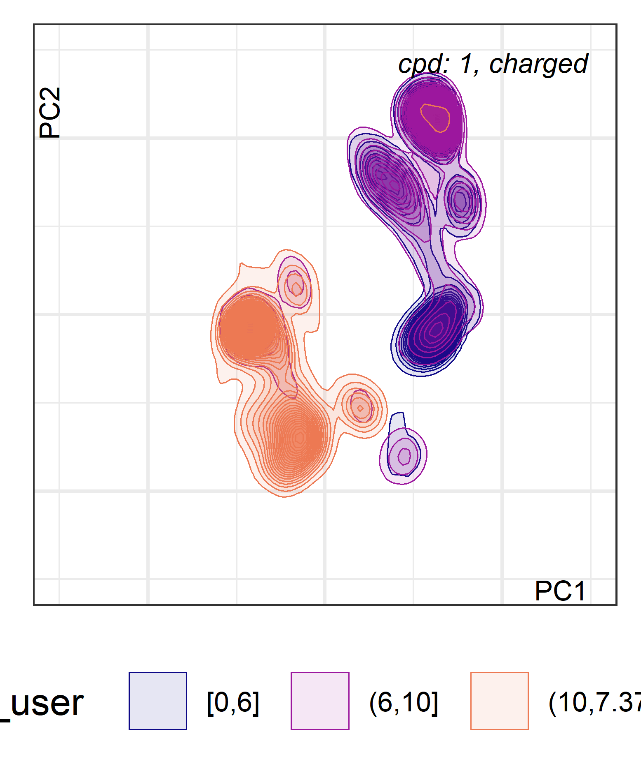


c)
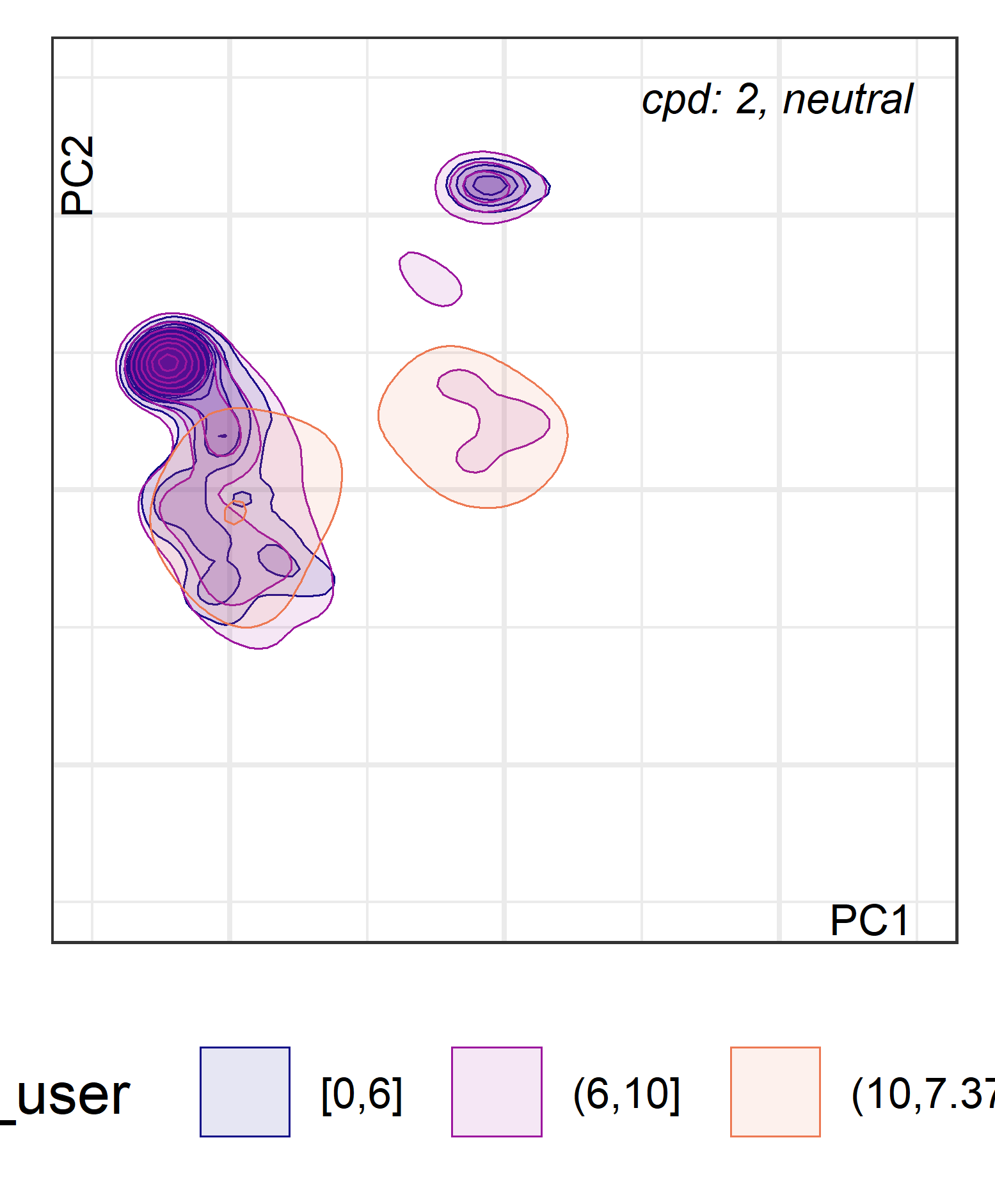
d)
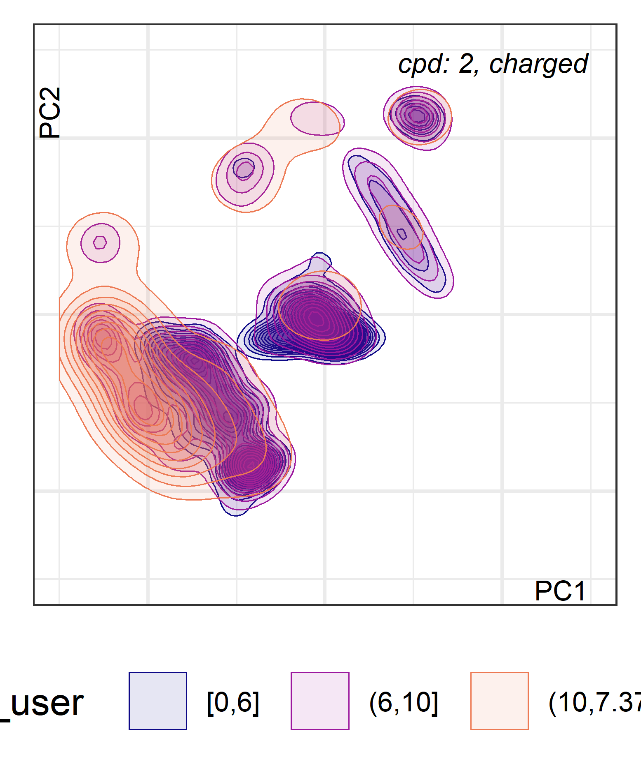


e)
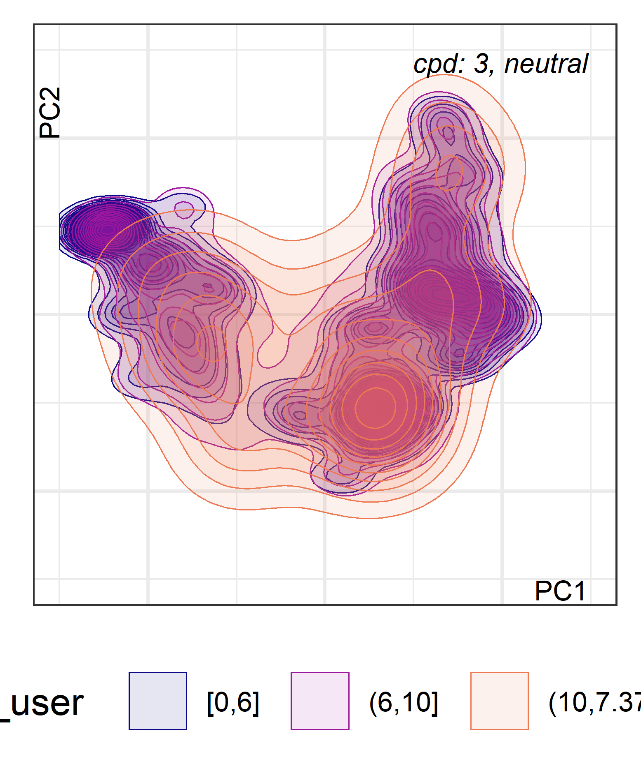
f)
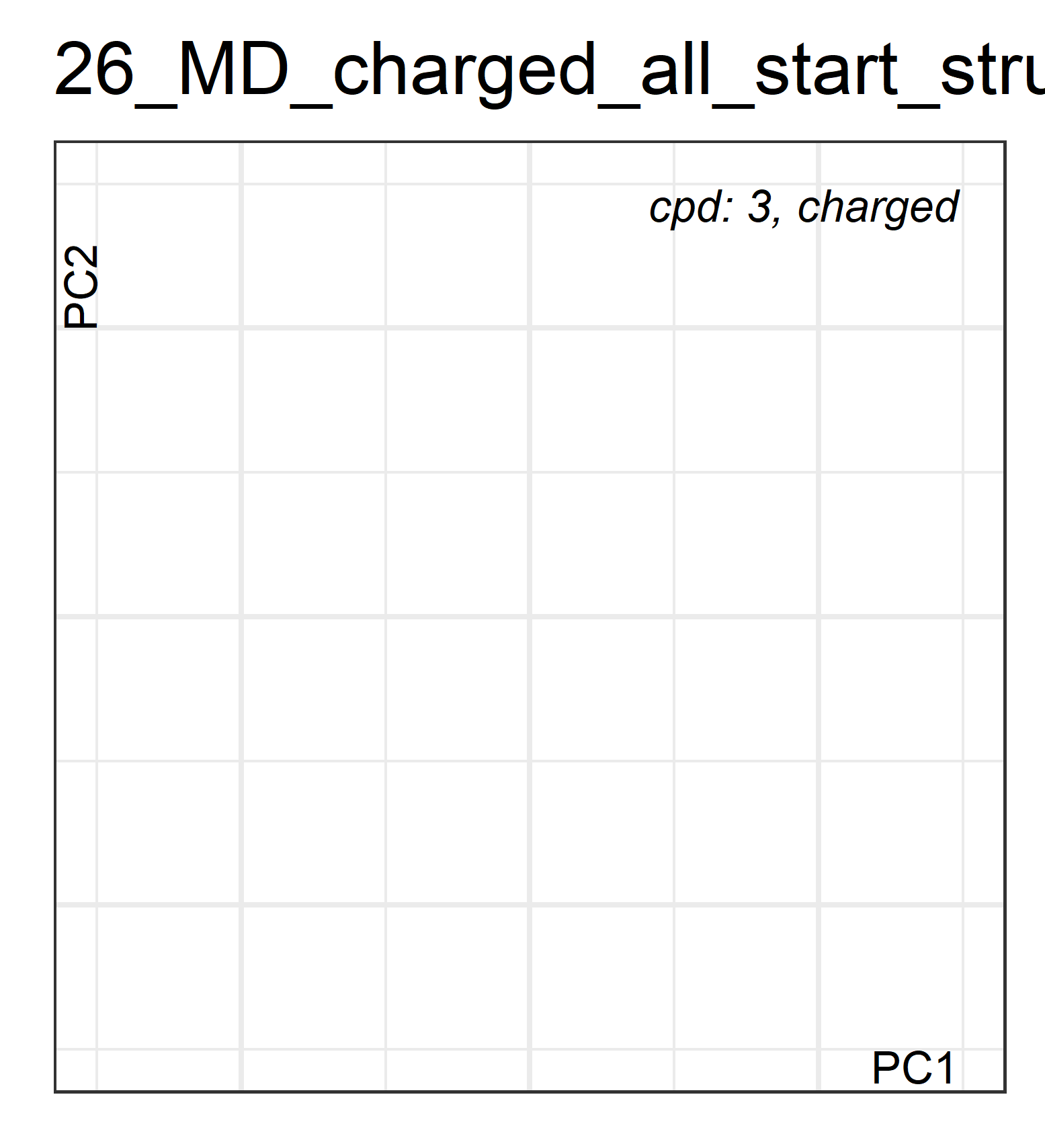


g)
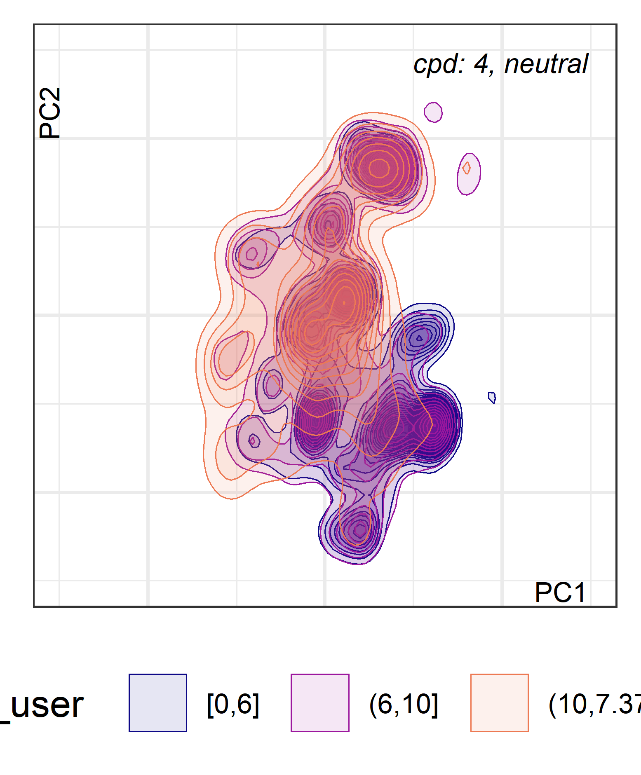
h)
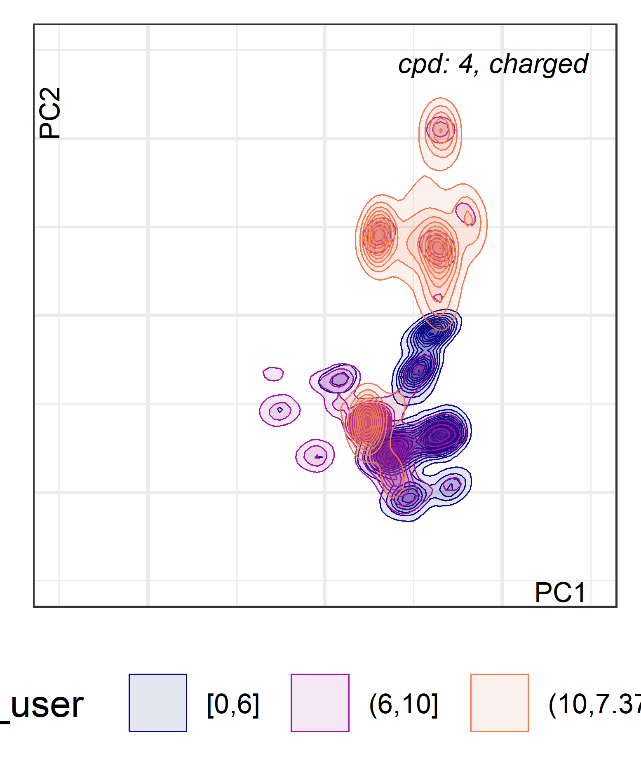


i)
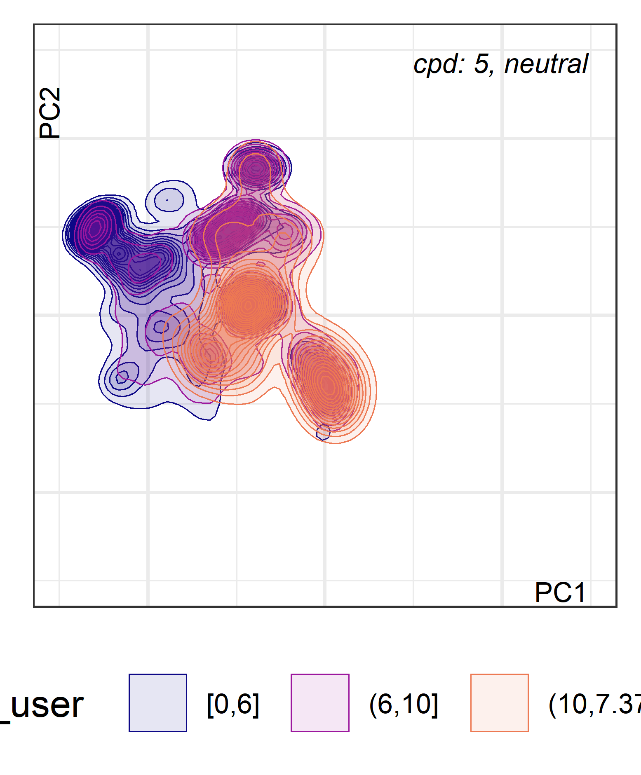
j)
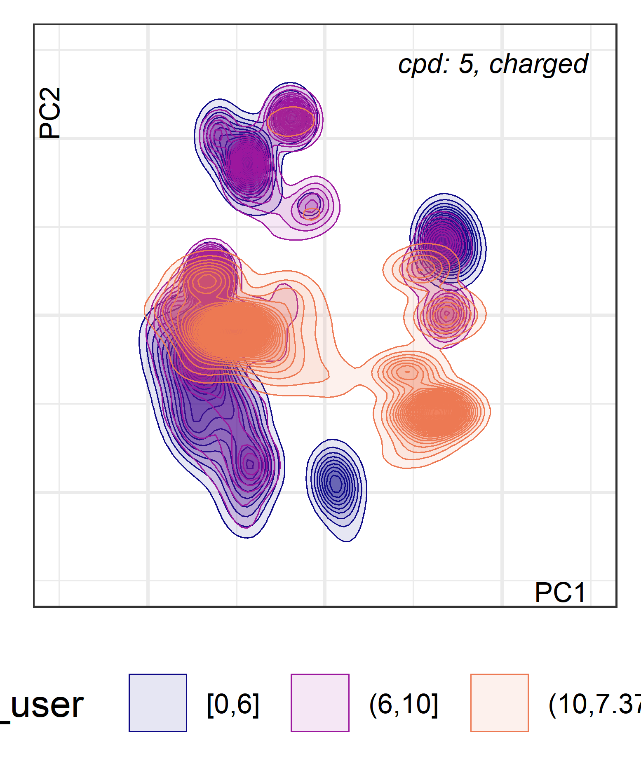


k)
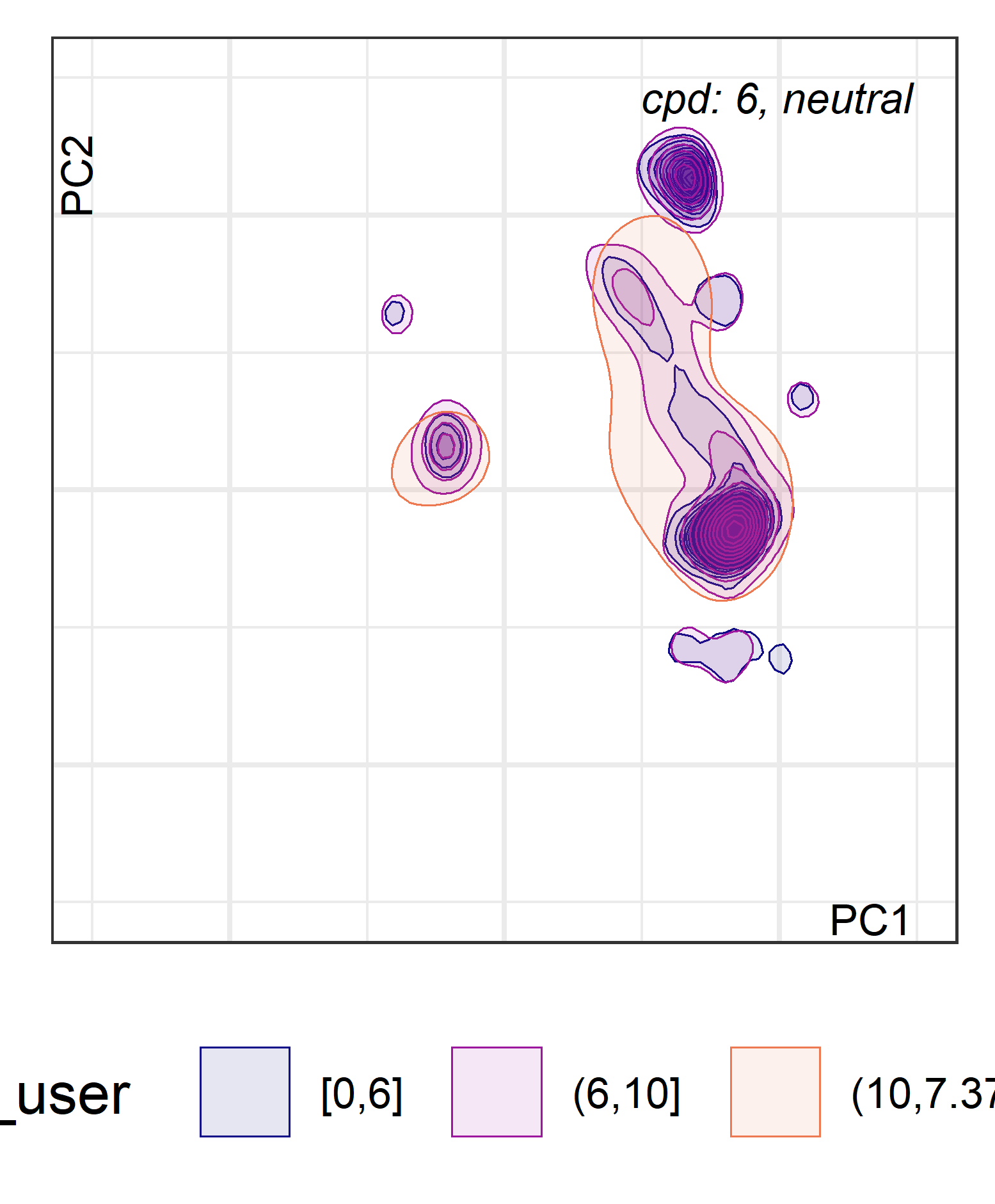
l)
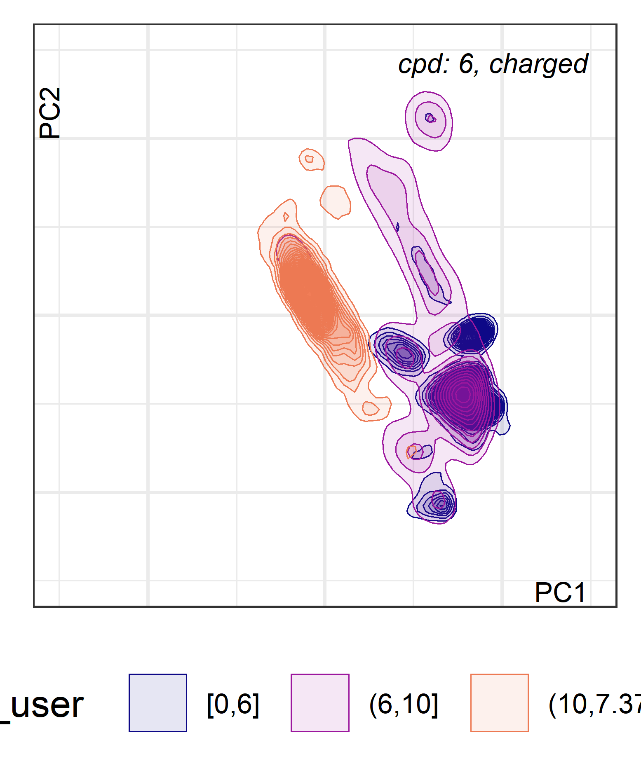


**Figure S4:** Conformer maps for macrocycles **1** to **6** in solvent CHCl_3_**,** color-coded by binned raw conformer relative energies with thresholds of 6 and 10 kcal mol^-1^ (conformers with relative energies higher than 100 kcal mol^-1^ were filtered out). Plots a), c), e), g), i), k) show maps for neutral and plots b), d), h), j), l) for positively charged ligands (ligand **3** is N-methylated, therefore not charged).

a)
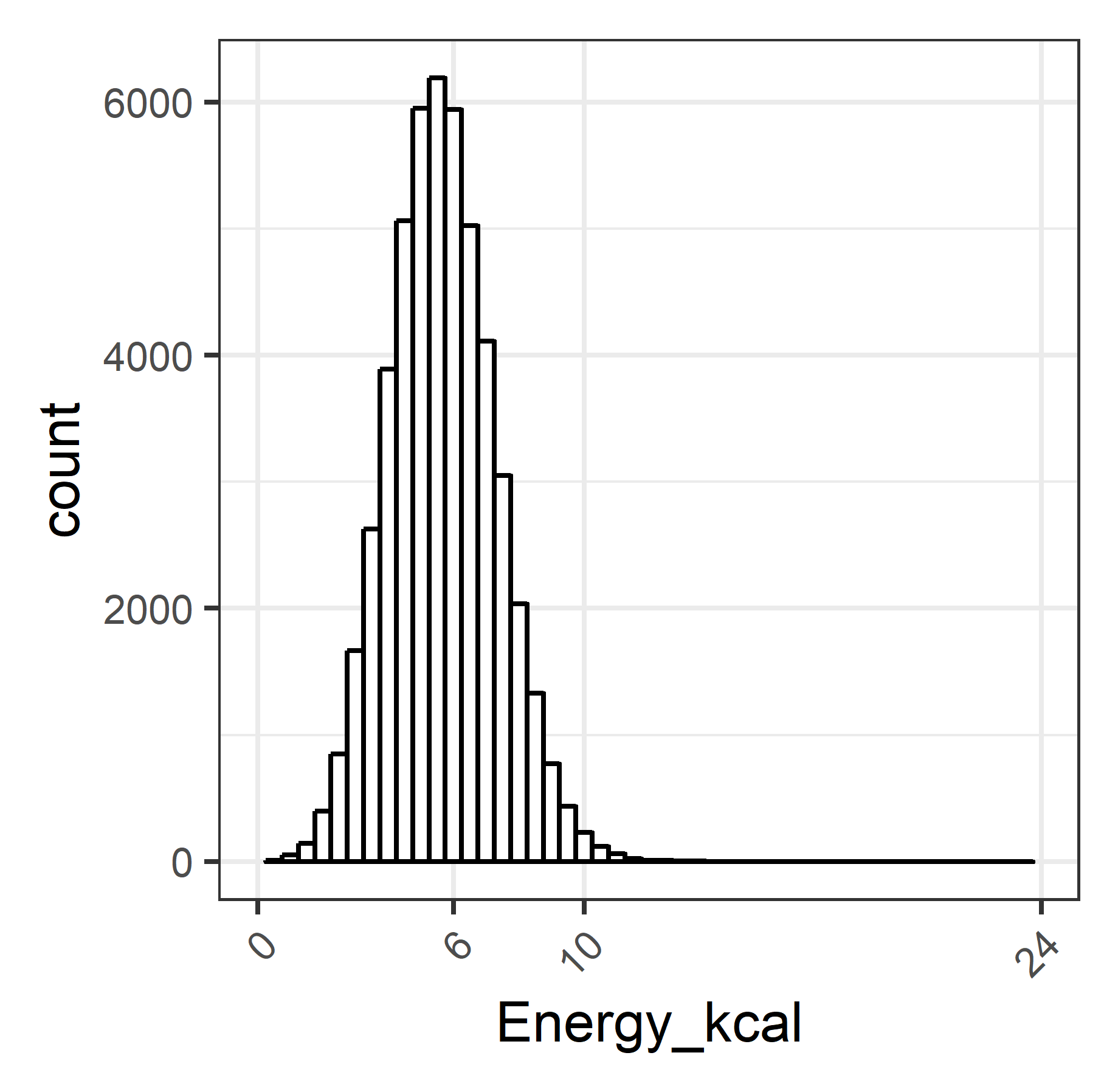
b)
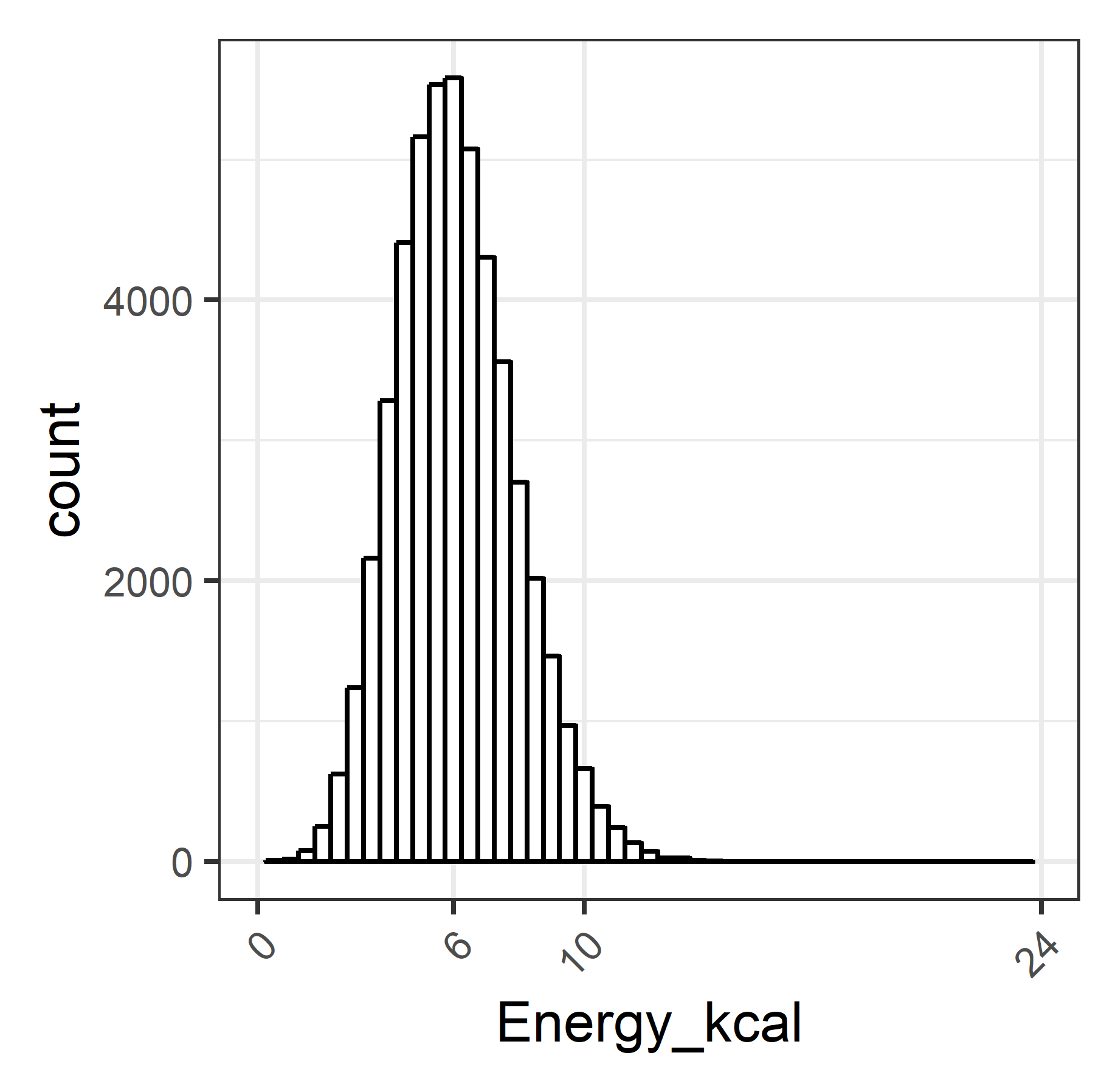


c)
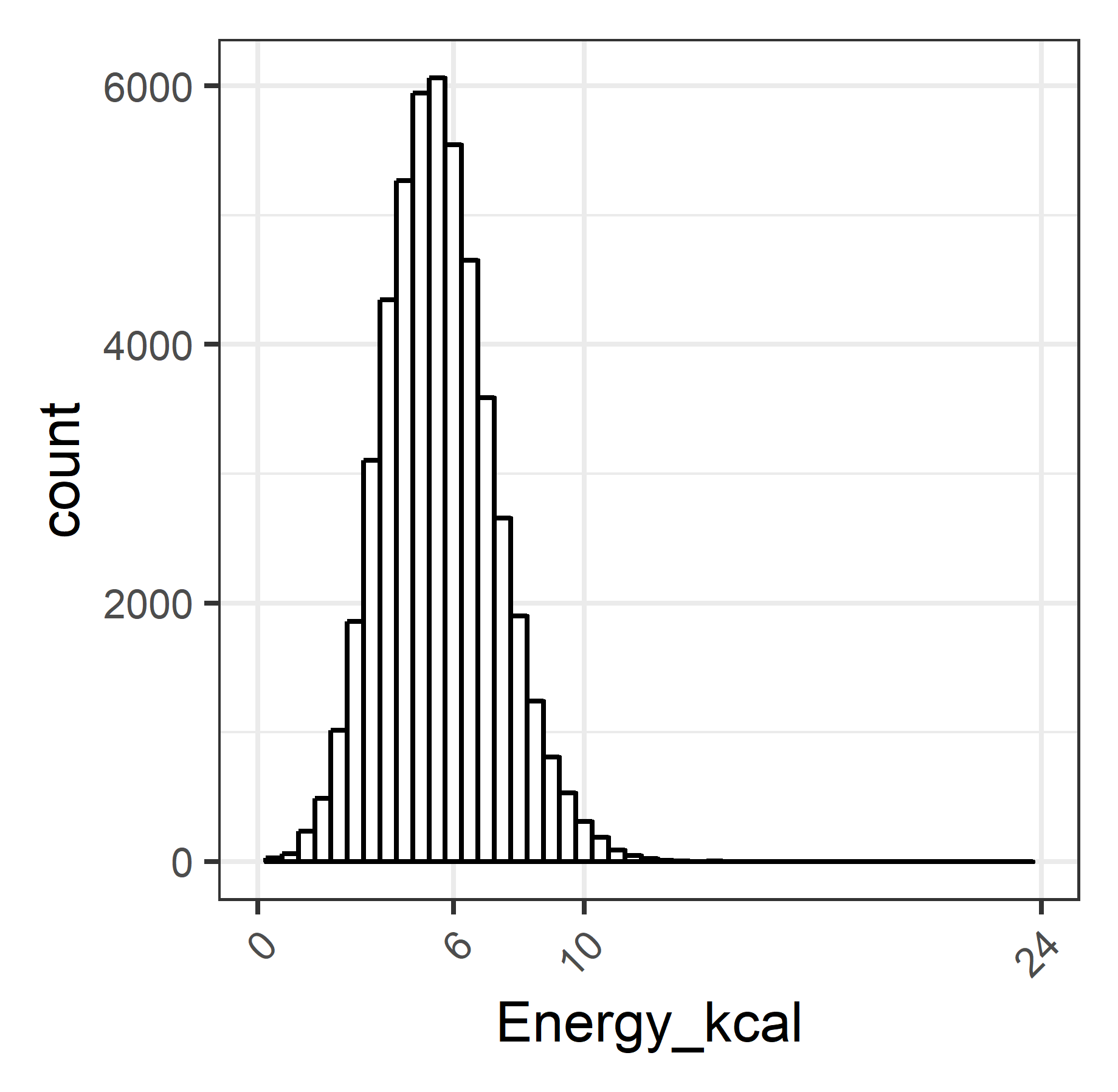
d)
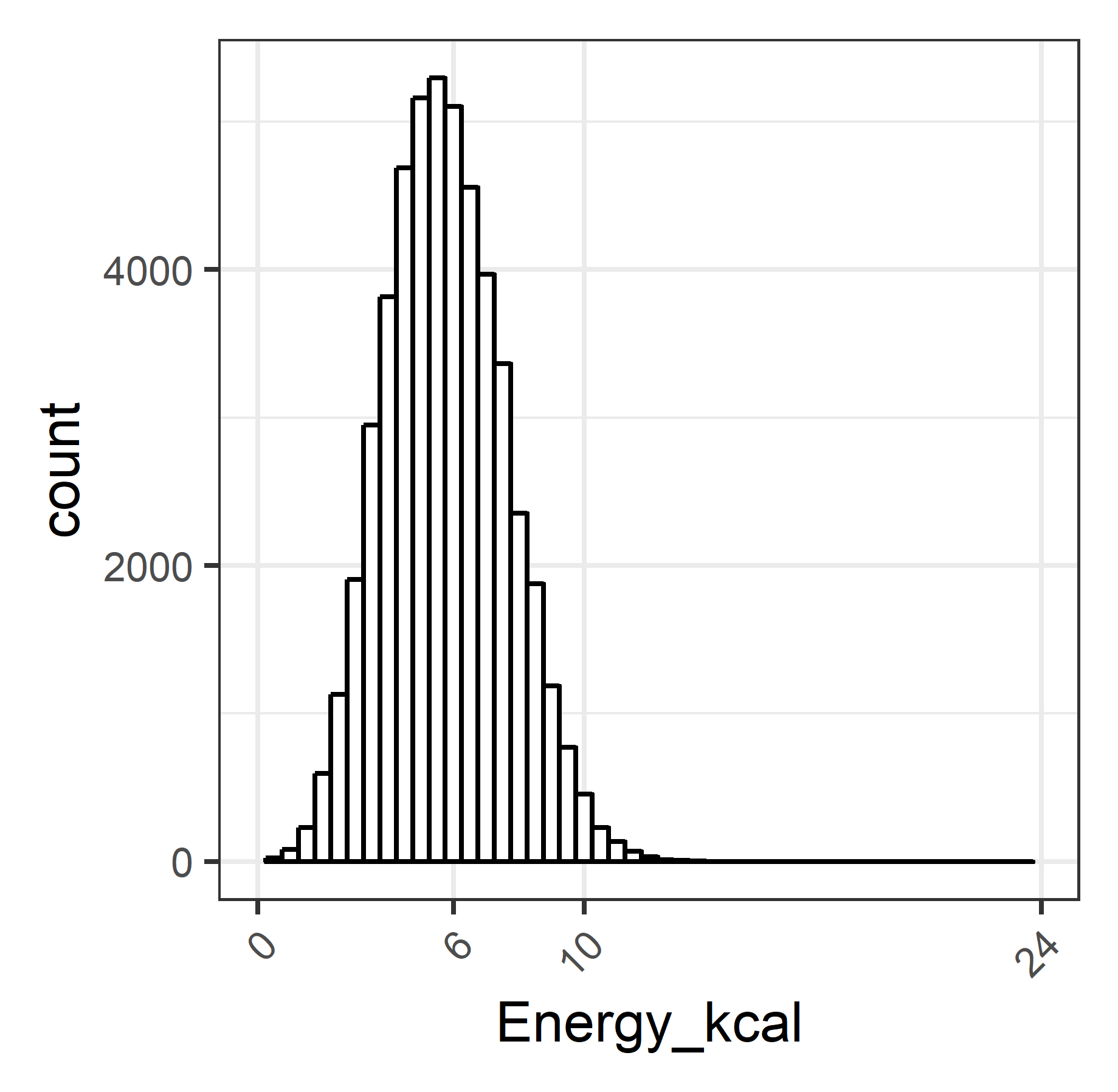


e)
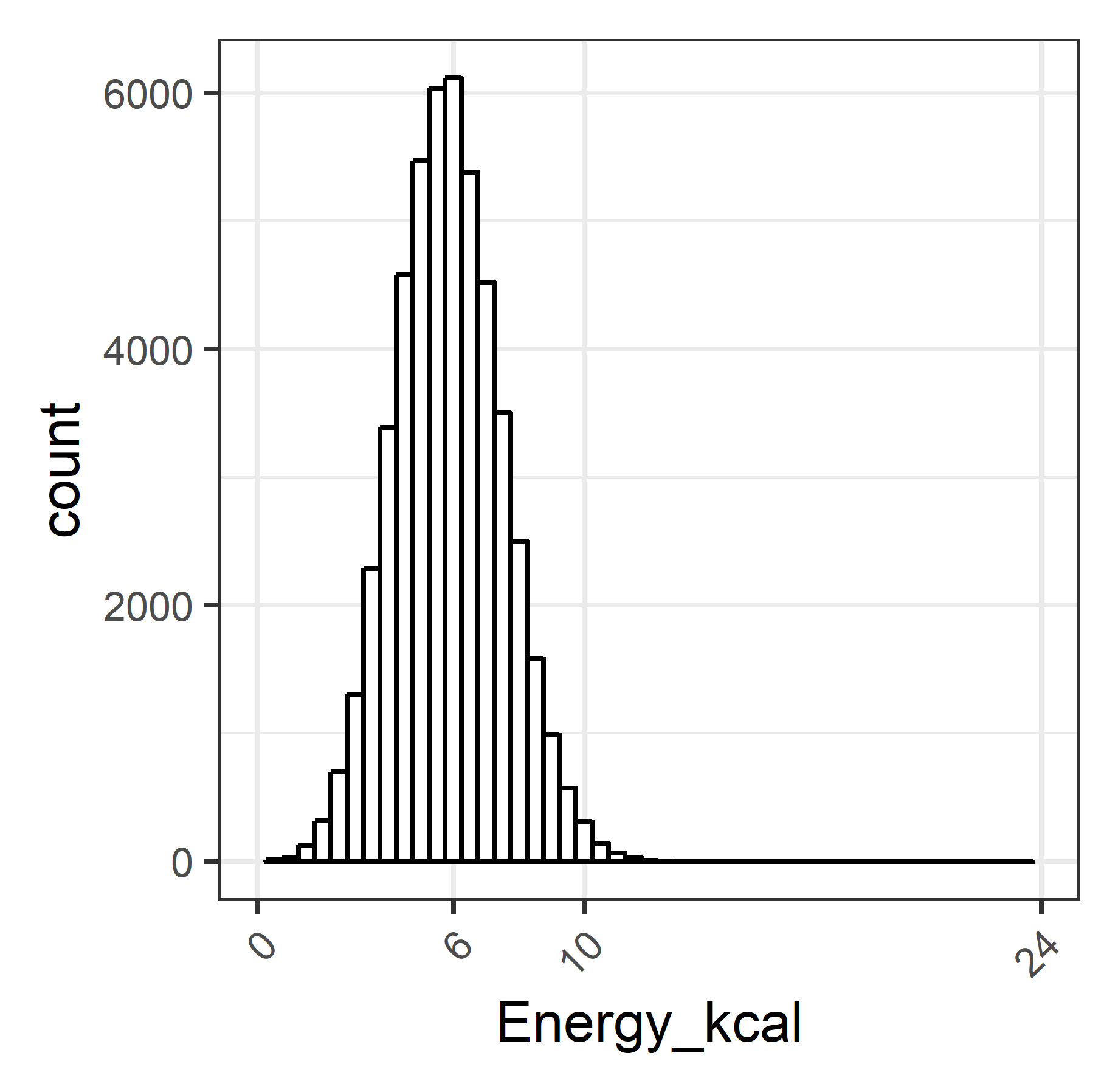
f)

g)
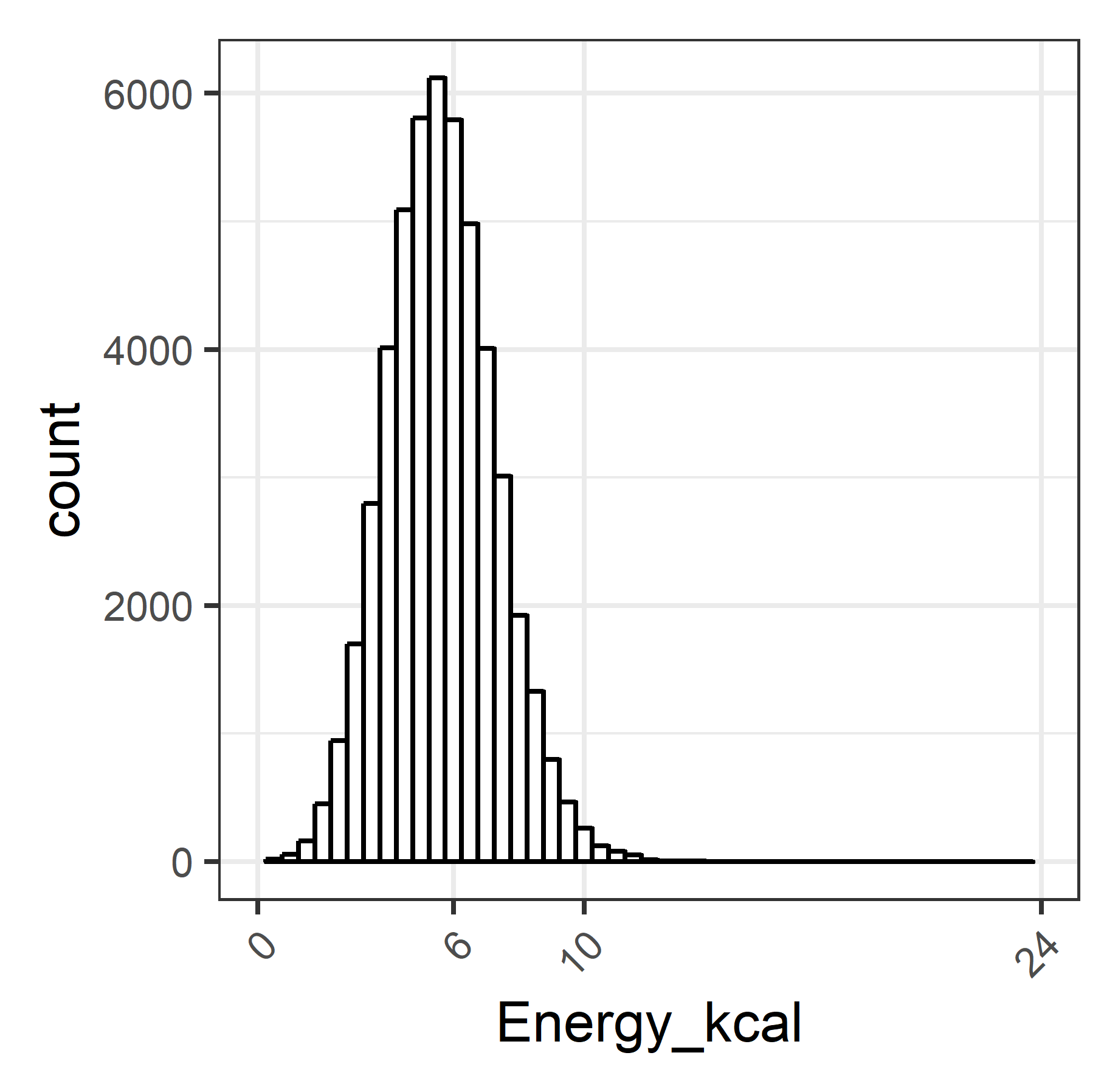
h)
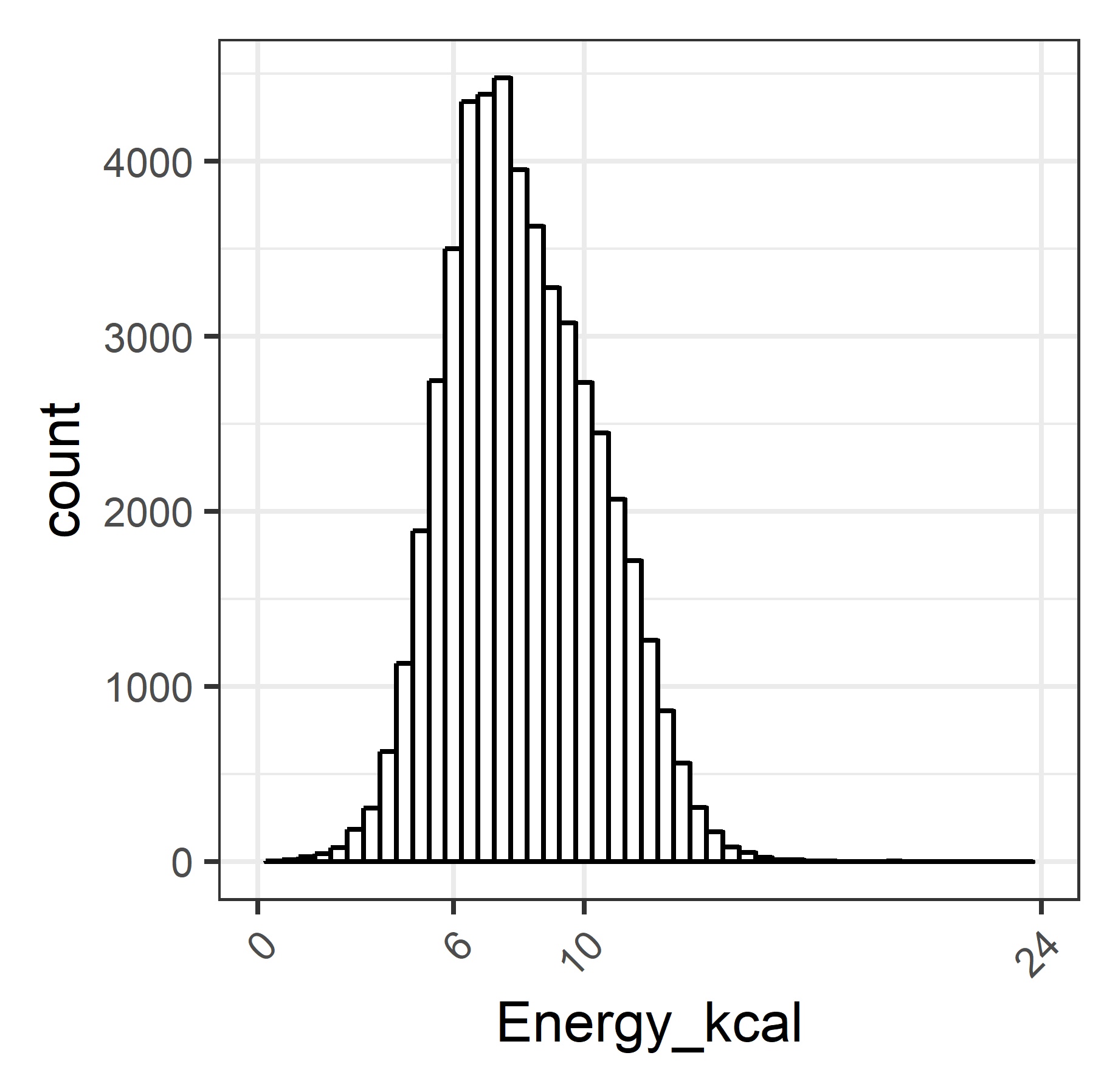


i)
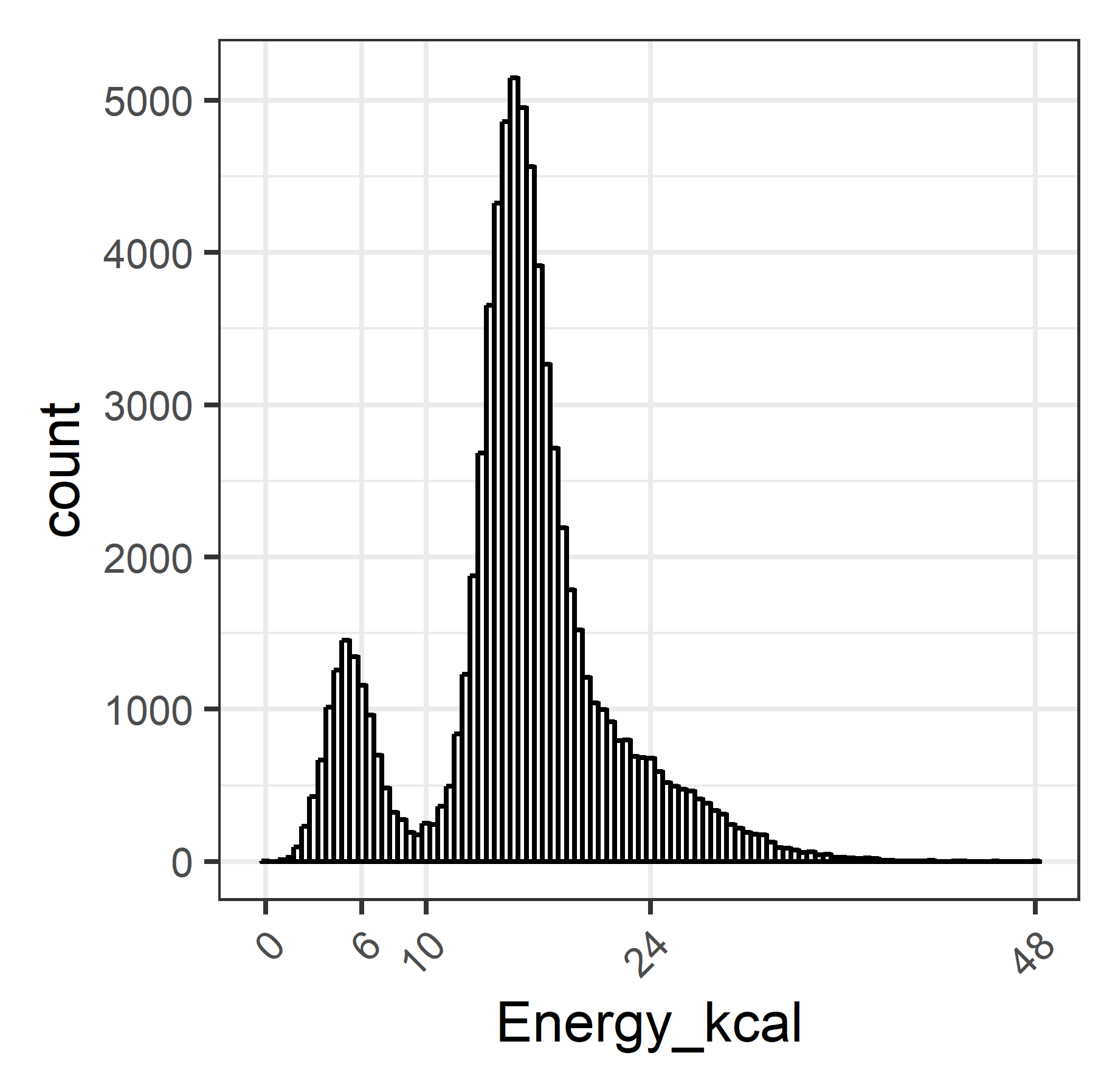
j)
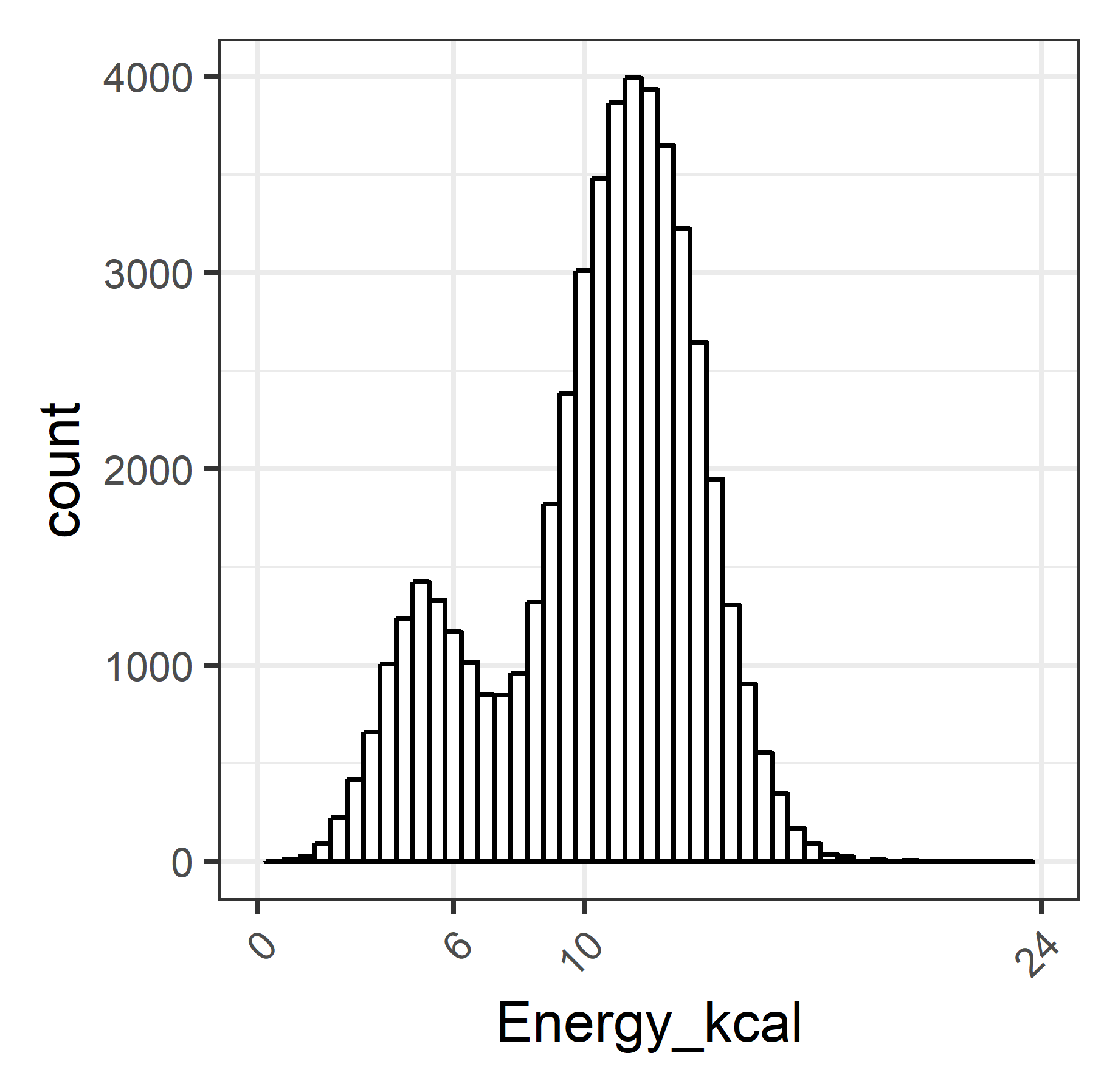


k)
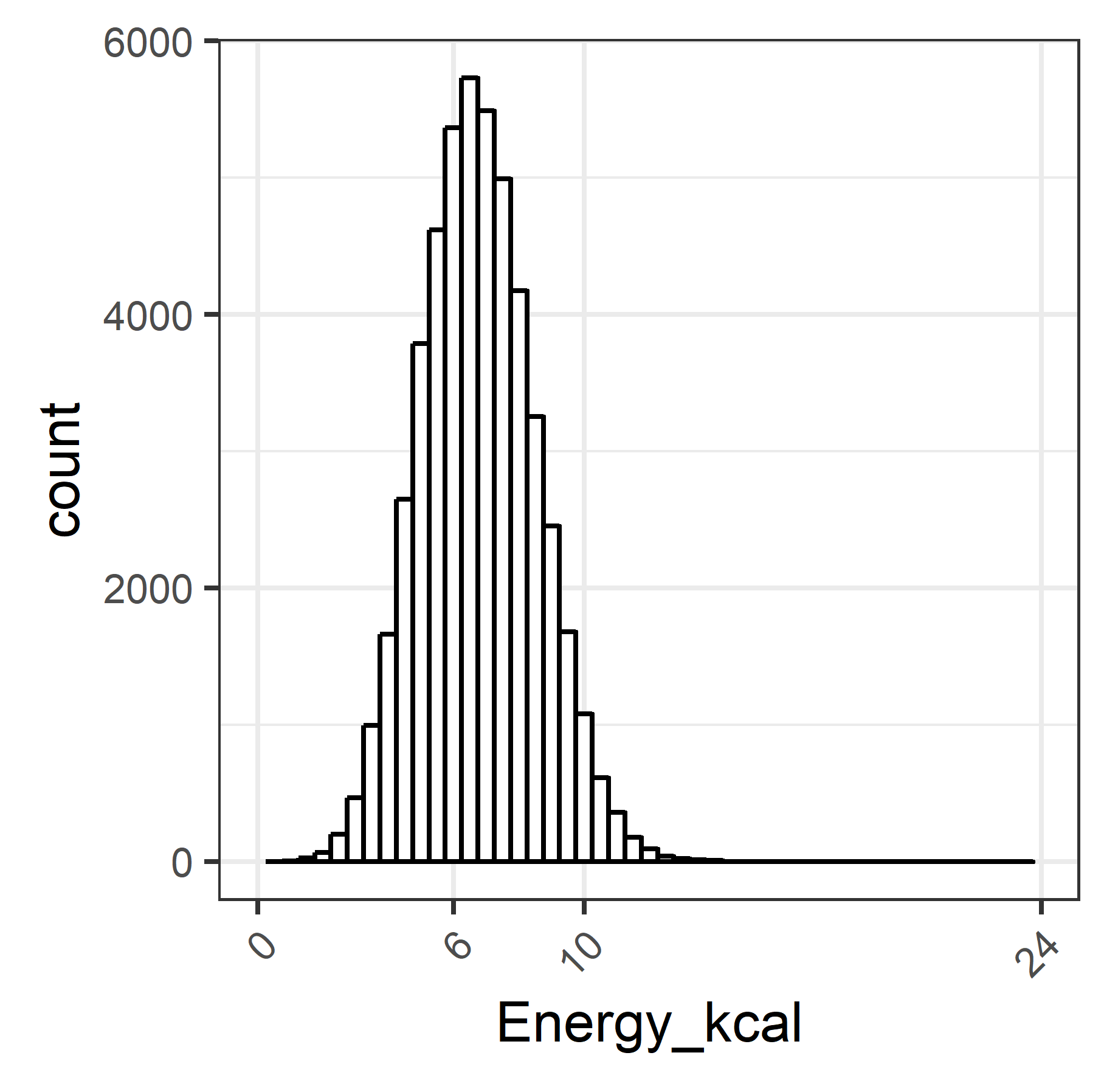
l)
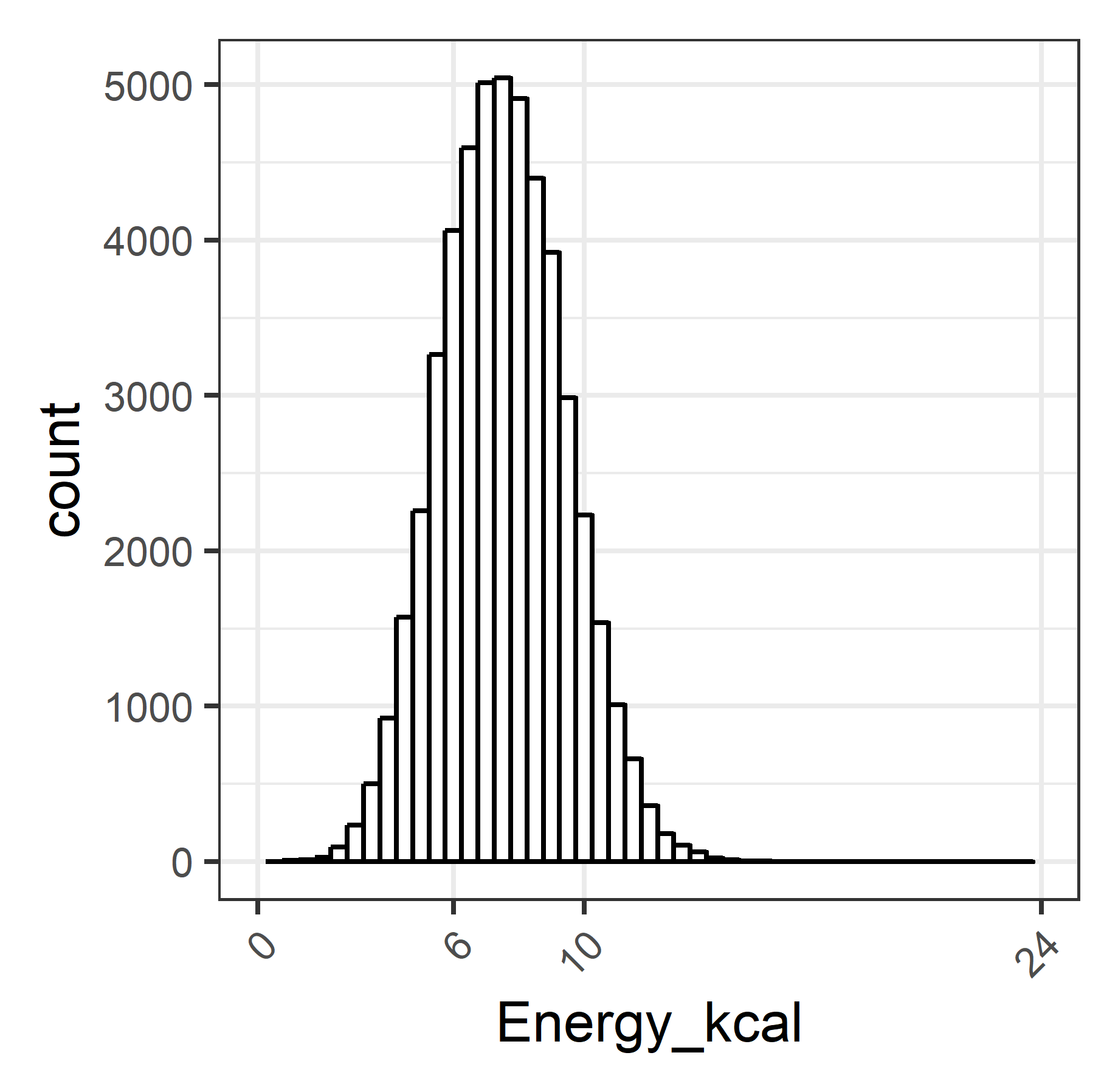


m)
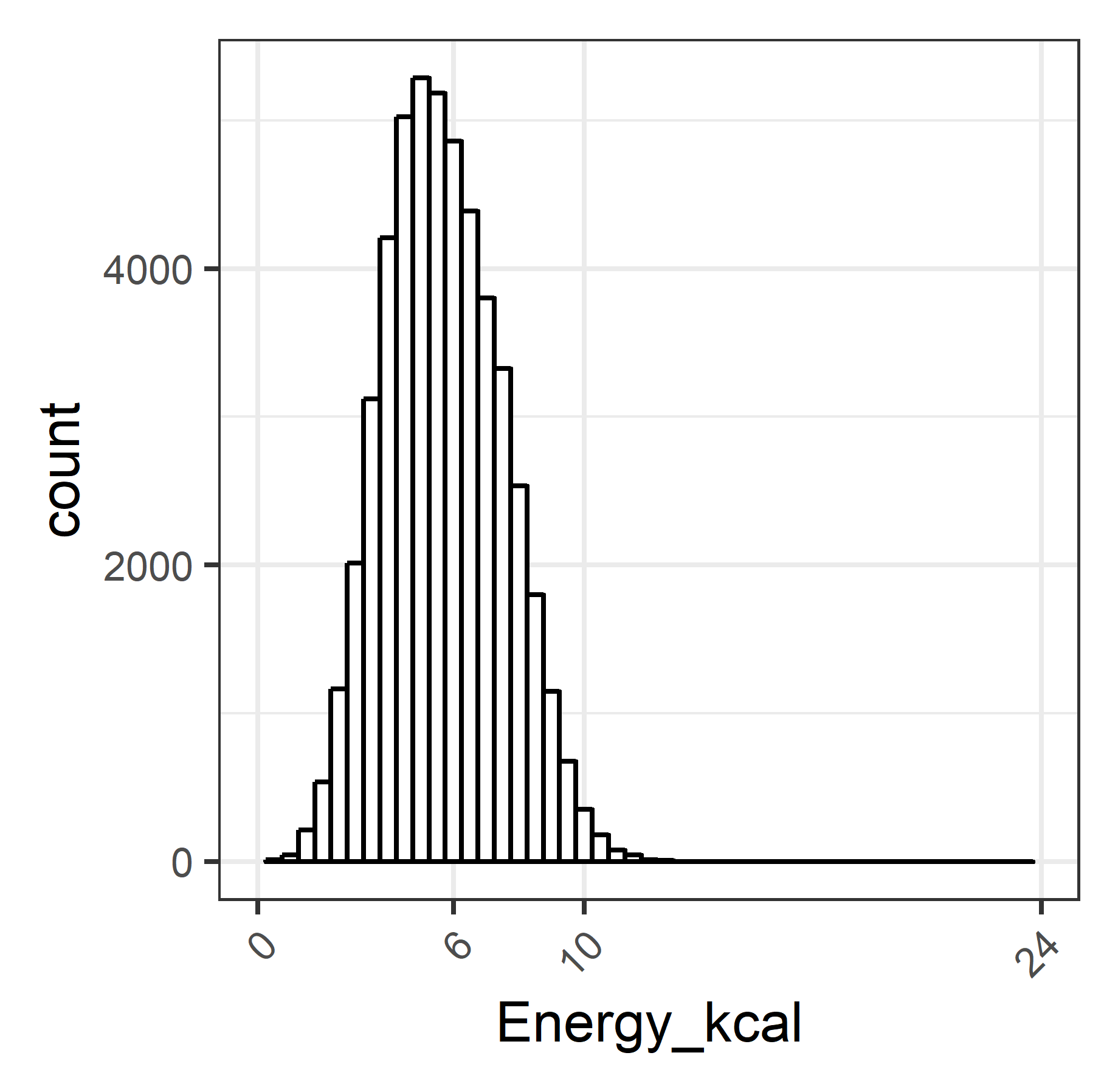

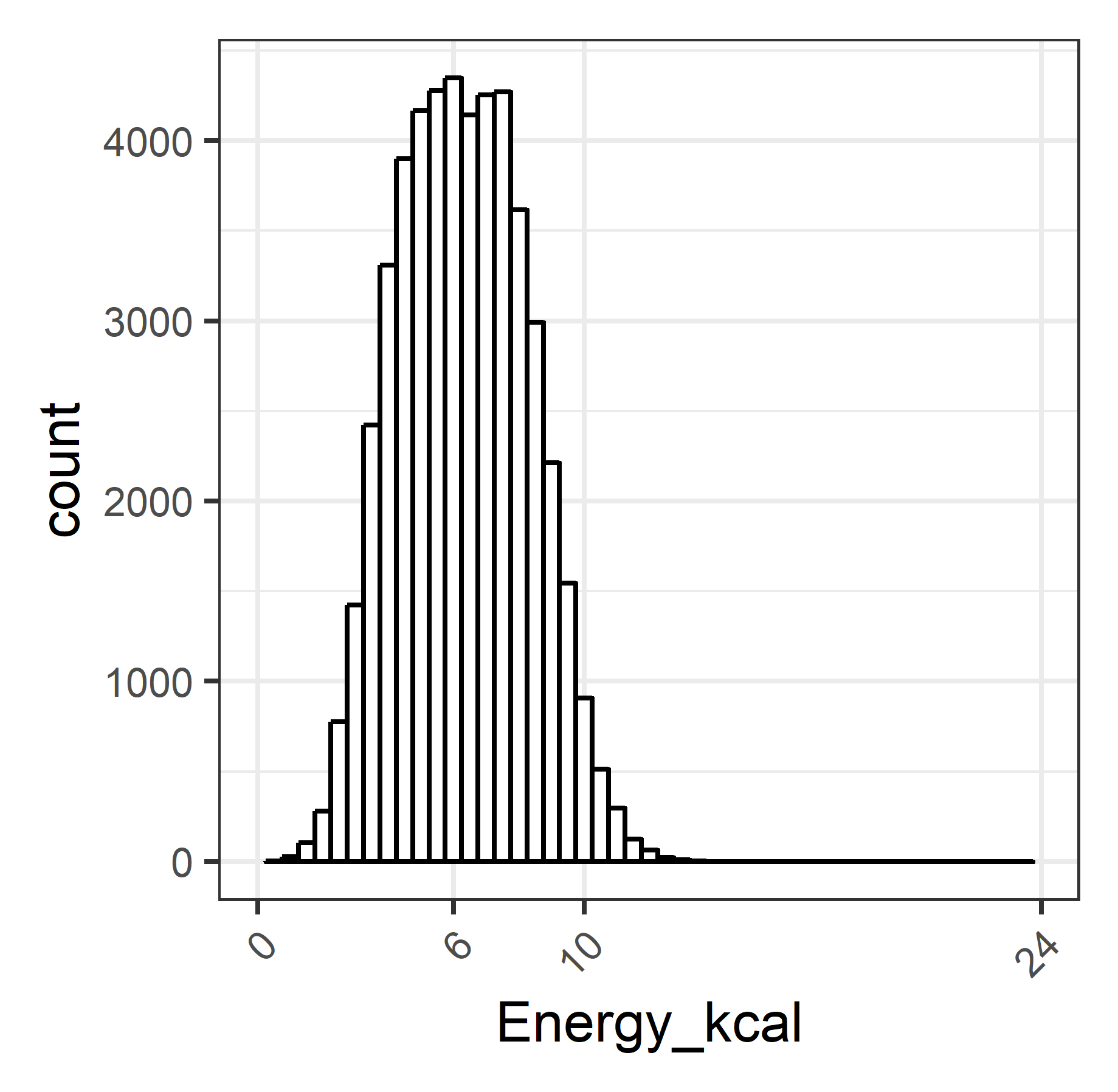
n)

**Figure S5:** Histograms of the energy distributions of the MD snapshots for macrocycles **1** to **7** in solvent water. Plots a), c), e), g), i), k) m) show maps for neutral and plots b), d), h), j), l) for positively charged ligands (ligand **3** is N-methylated, therefore not charged). The histograms for neutral form of 5 were derived from 8 starting conformers.

1.
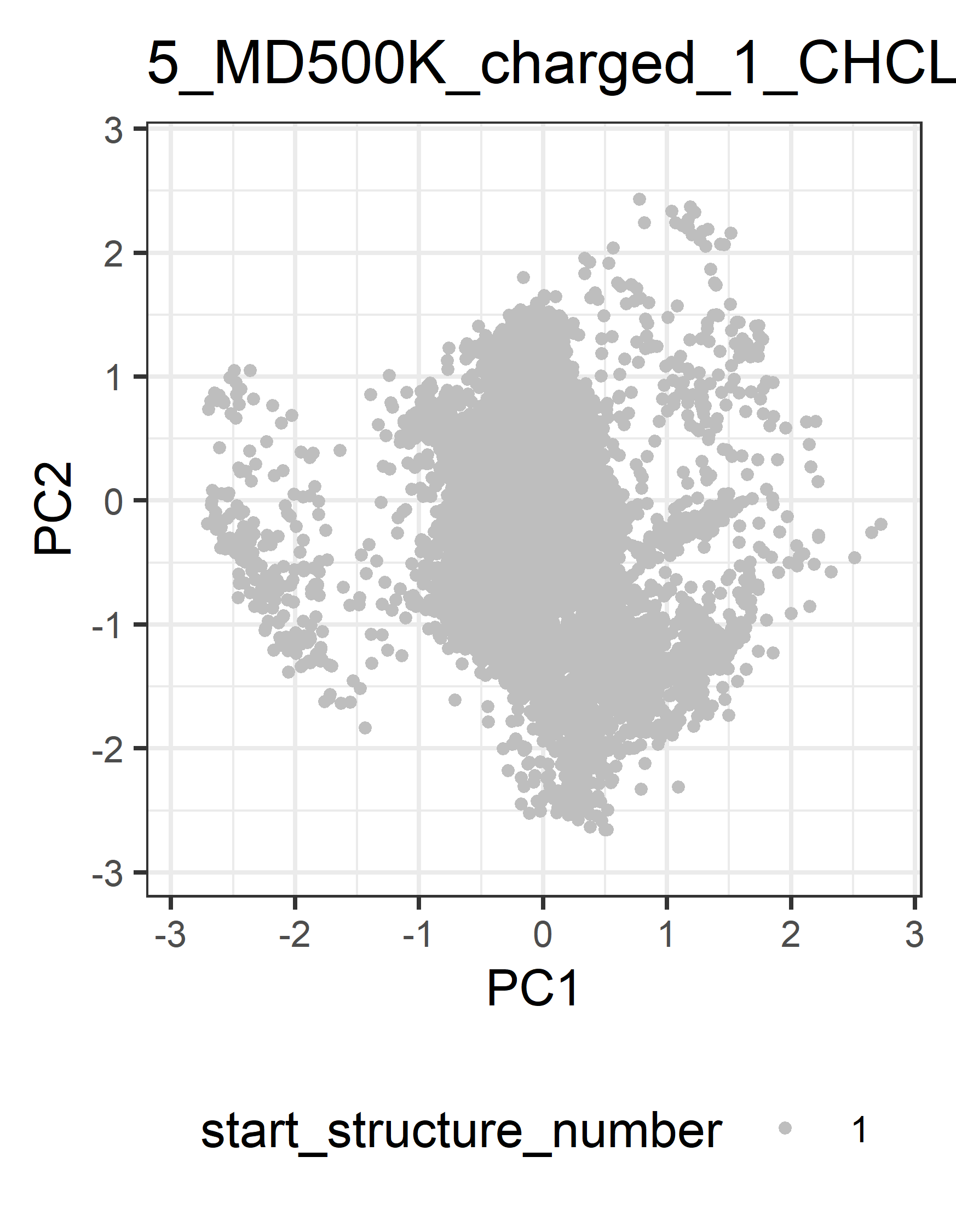

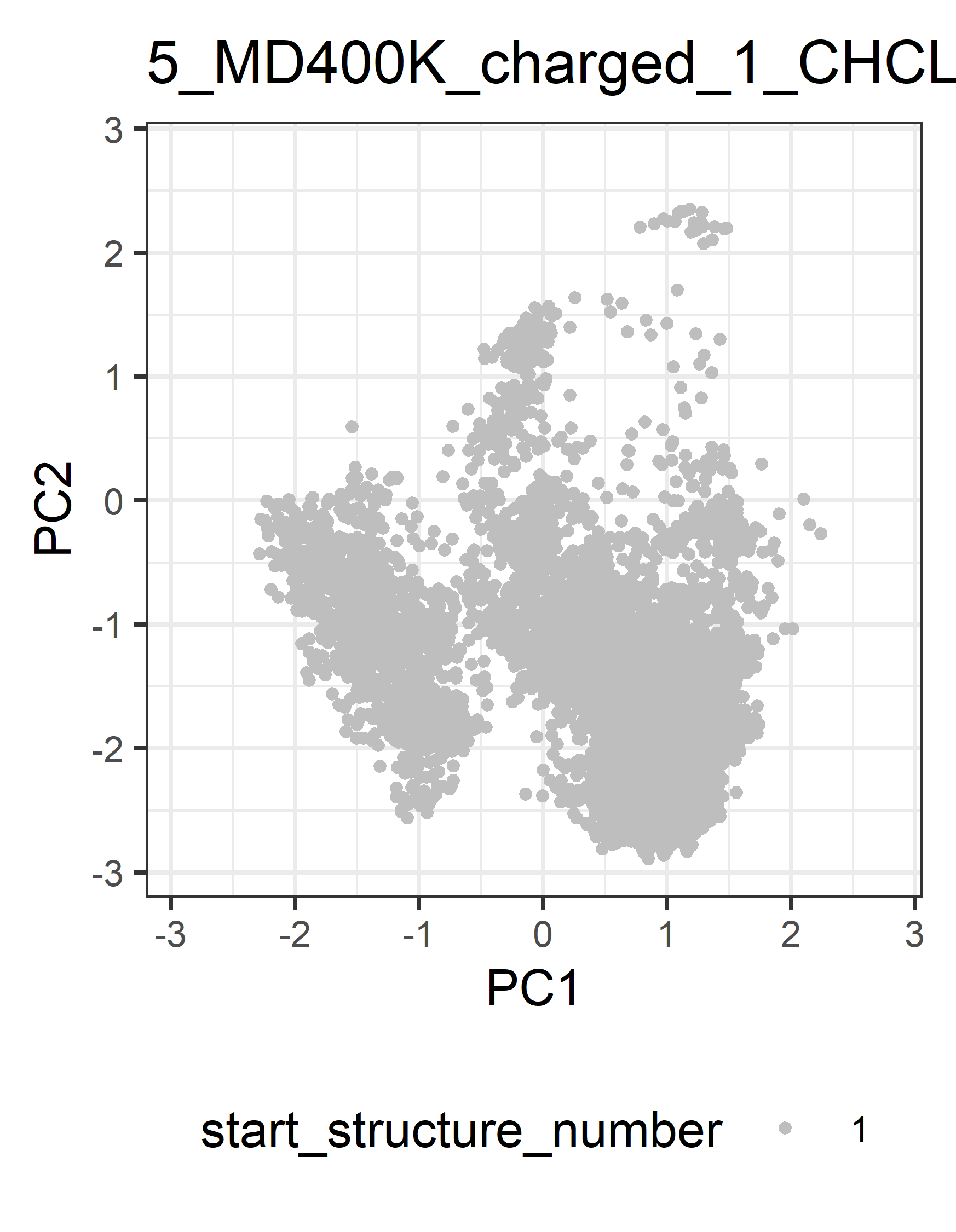

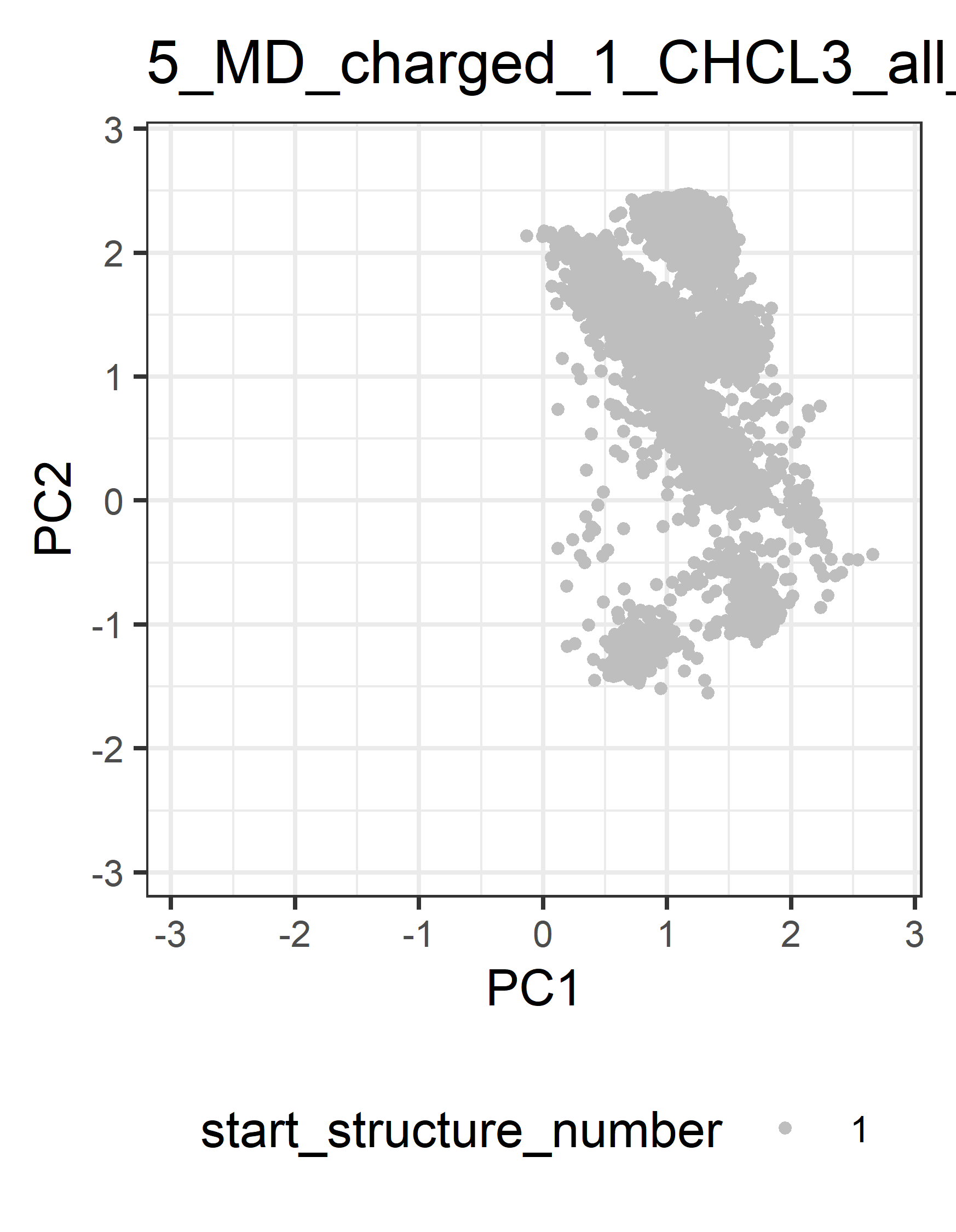
 b) c)

**
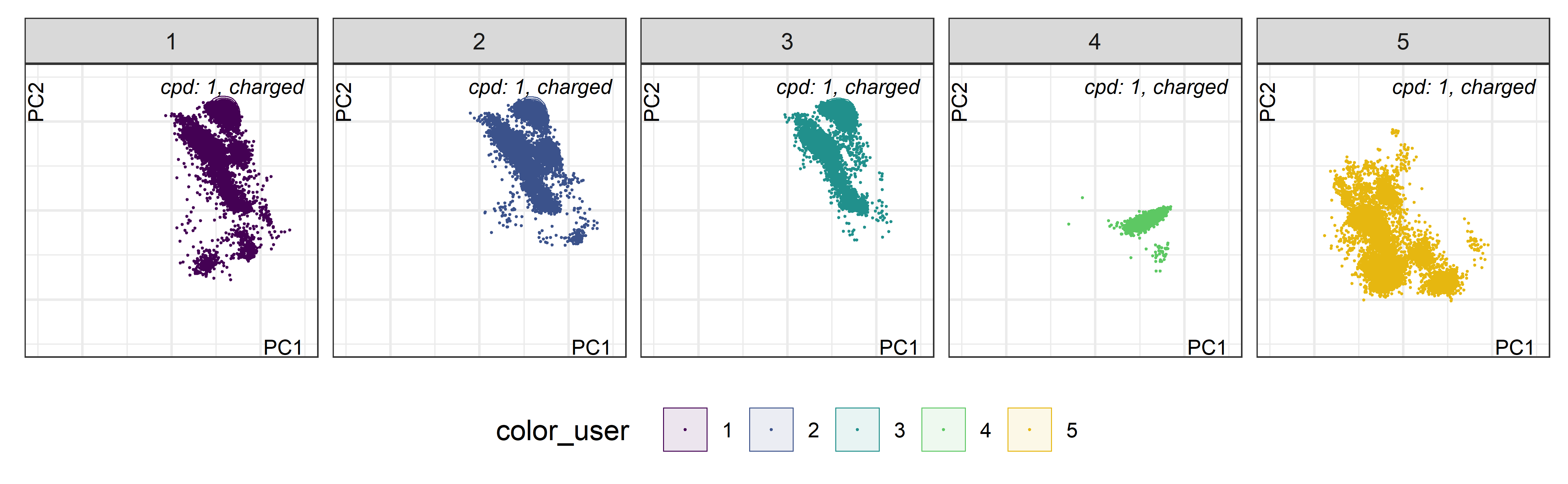
**

d)

**Figure S6:** Conformer ensembles for charged compound **1** in solvent CHCL_3_. a) one starting conformer at T = 300 K; b) one starting conformer at T = 400 K; c) one starting conformer at T = 500 K; d) five different starting conformers at T = 300 K, color-coded by starting conformer.

a)
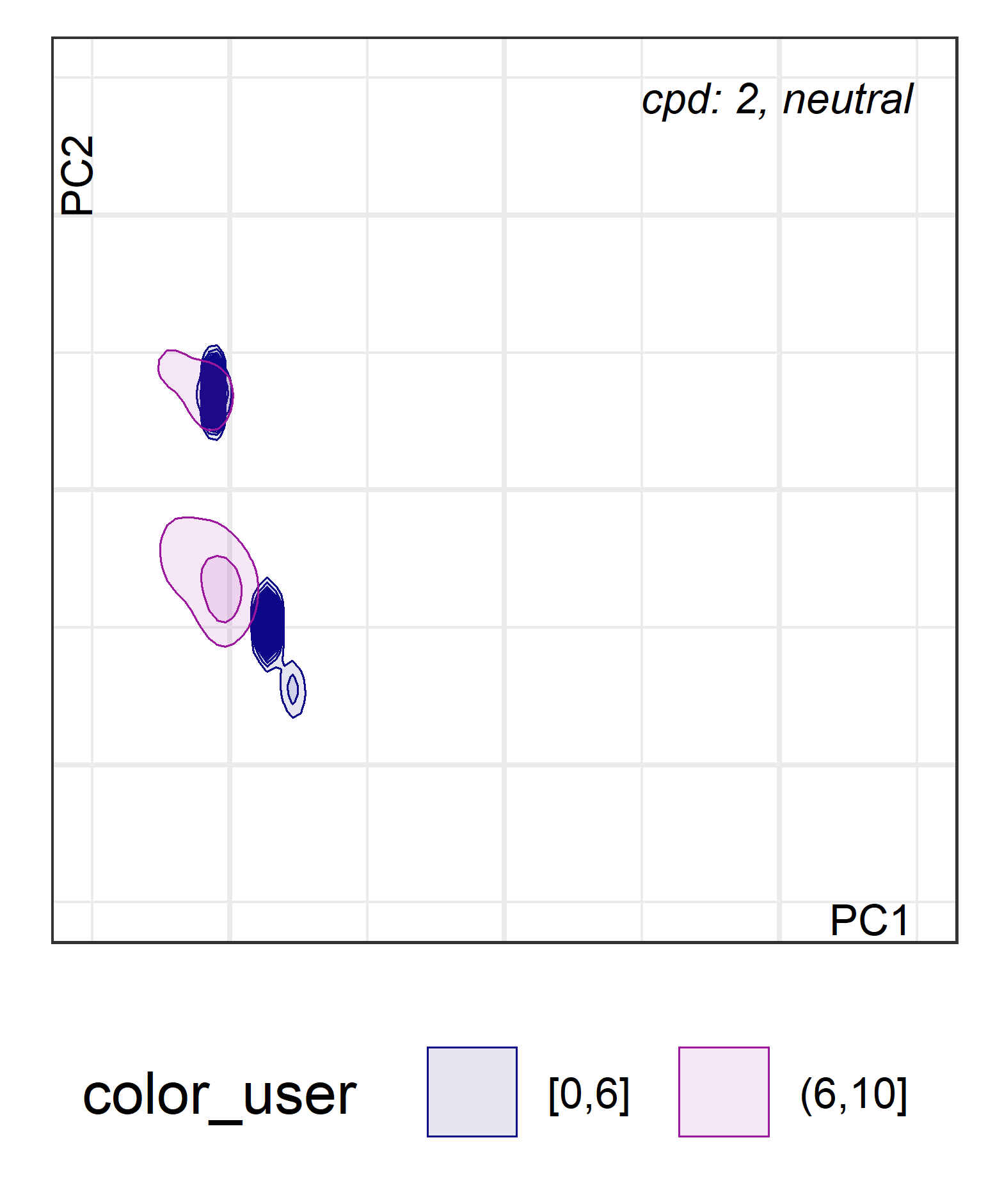
b)
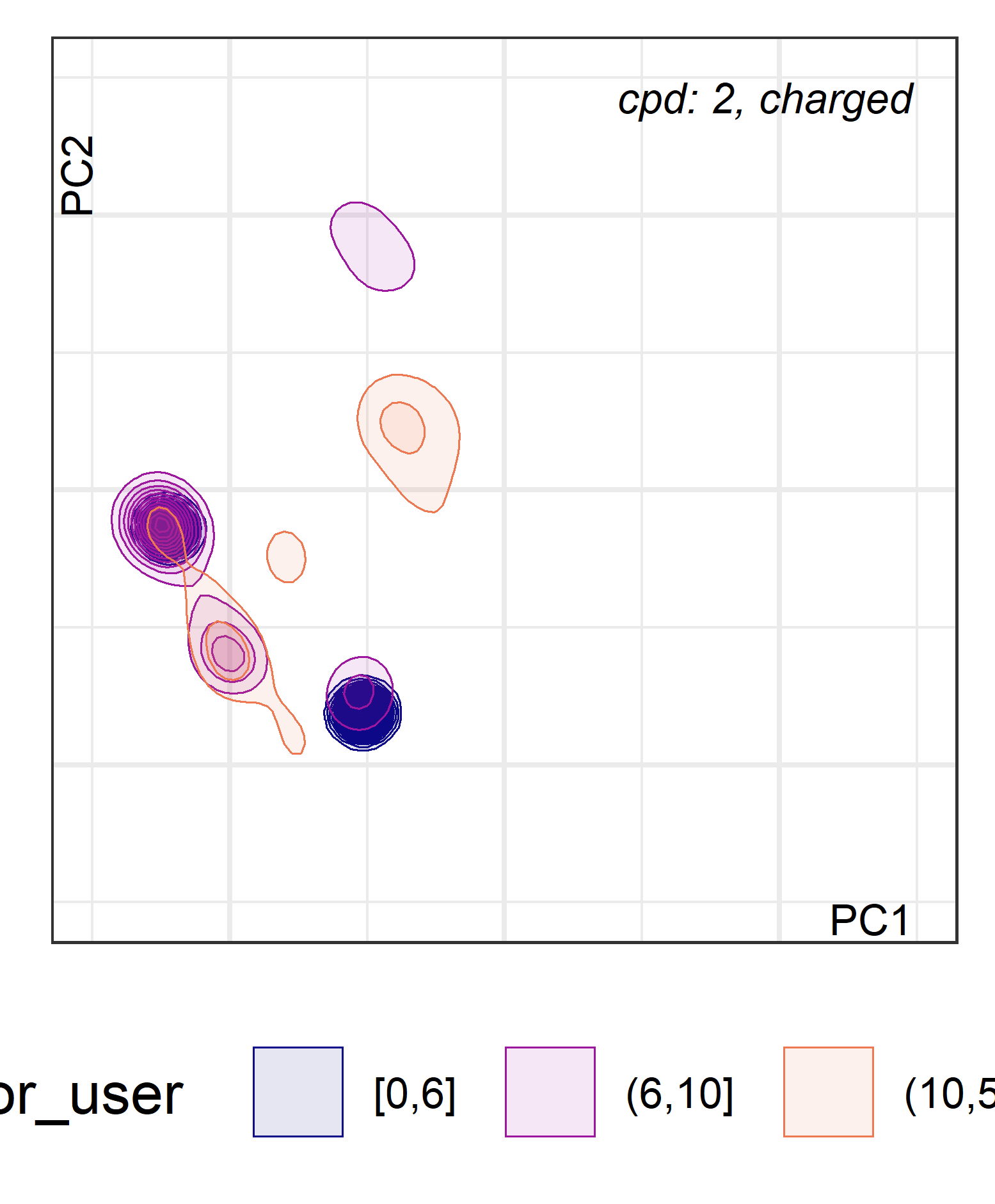


c)
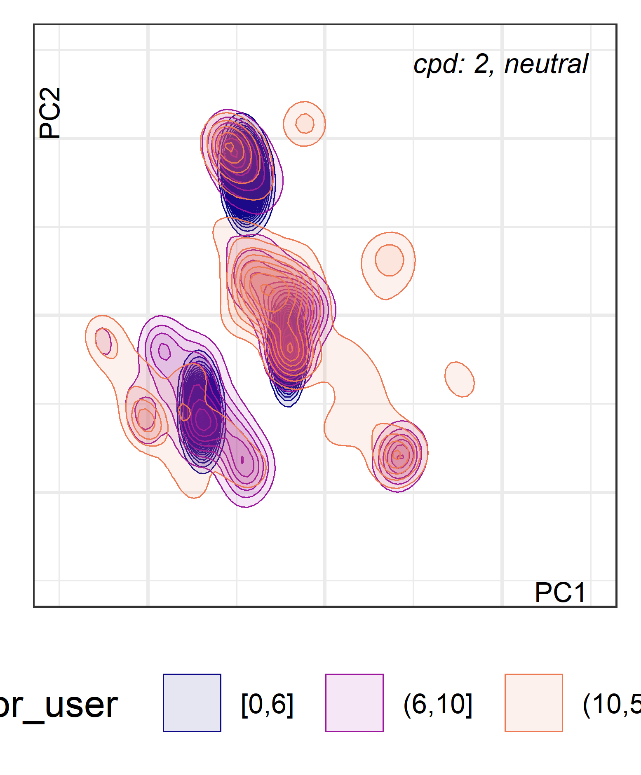
d)
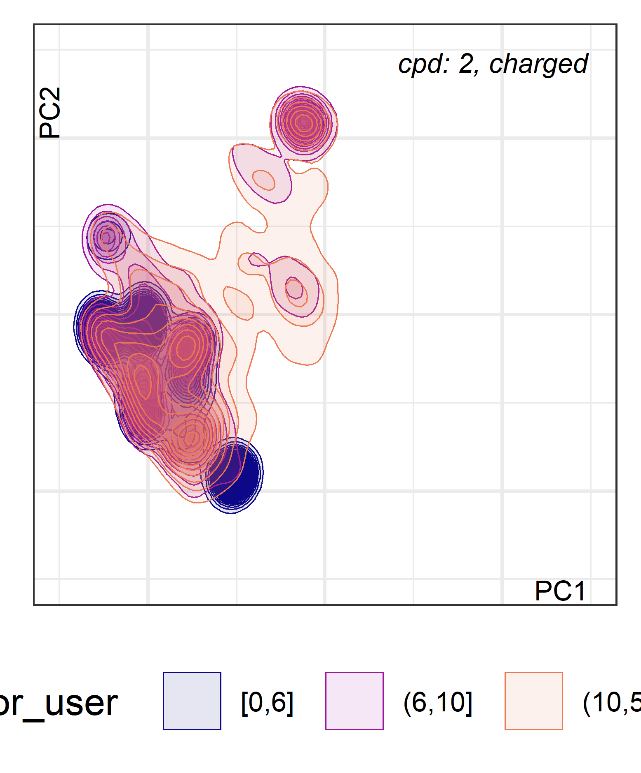


**Figure S7:** Ensembles for compound **2** in water generated by a simulated annealing protocol; a) and c) show the neutral, b) and d) the charged state. Plots a) and b) indicate that simulated annealing if performed with the lowest energy conformer from the MD run is not able to map the full ensemble space, whereas SA with 5 diverse starting conformers yields analog maps as MD with 5 diverse starting conformers. Color-coding is by binned raw conformer relative energies with thresholds of 6 and 10 kcal mol^-1^ (conformers with relative energies higher than 100 kcal mol^-1^ were filtered out). Blue is for low, pink for medium and orange for high-energy conformations.

**
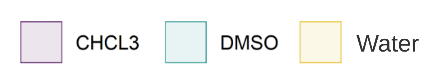
**

**1**
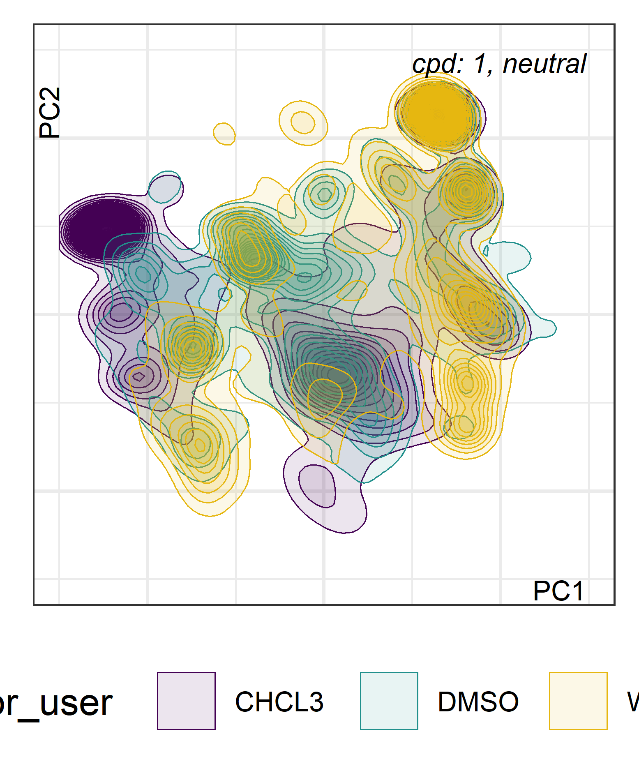
 **2**
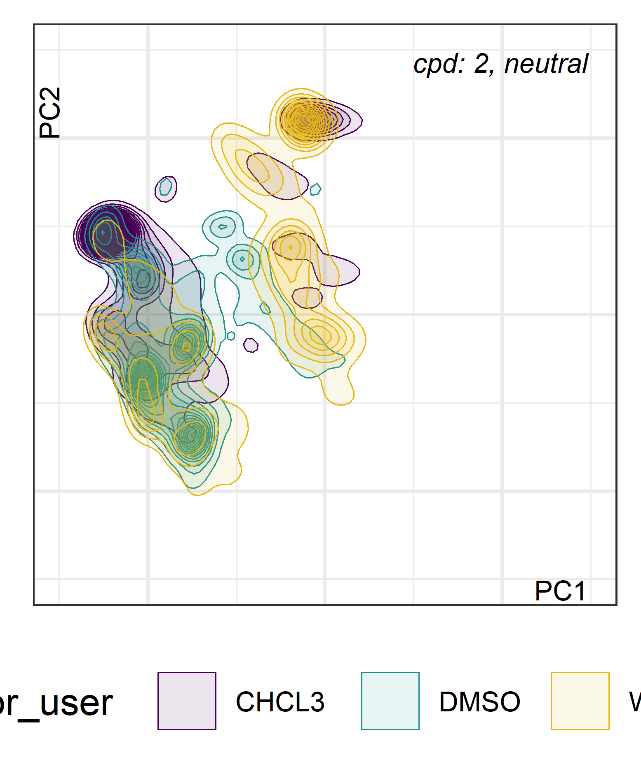


**3**
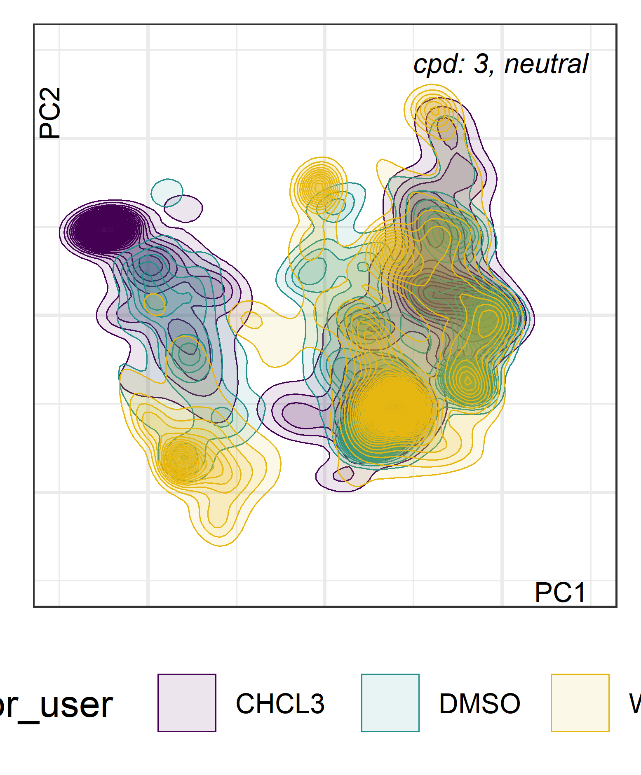
 **4**
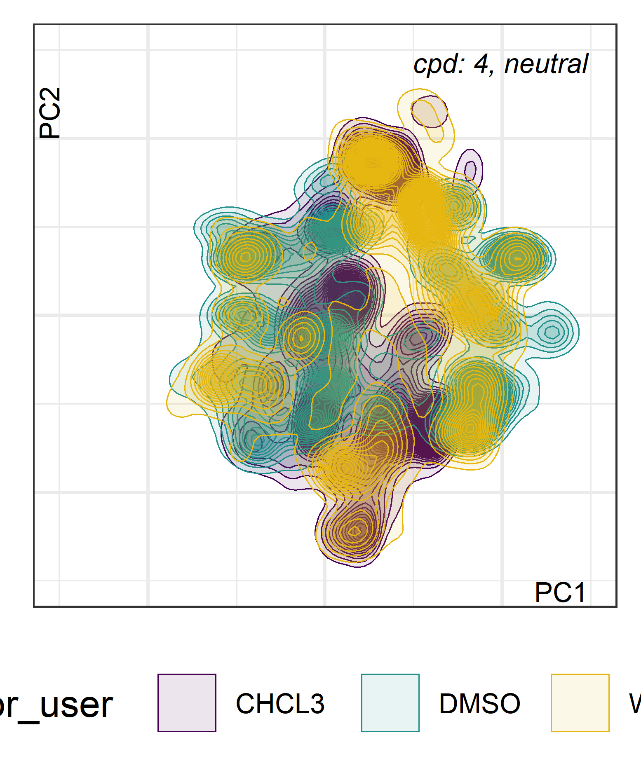


**5**
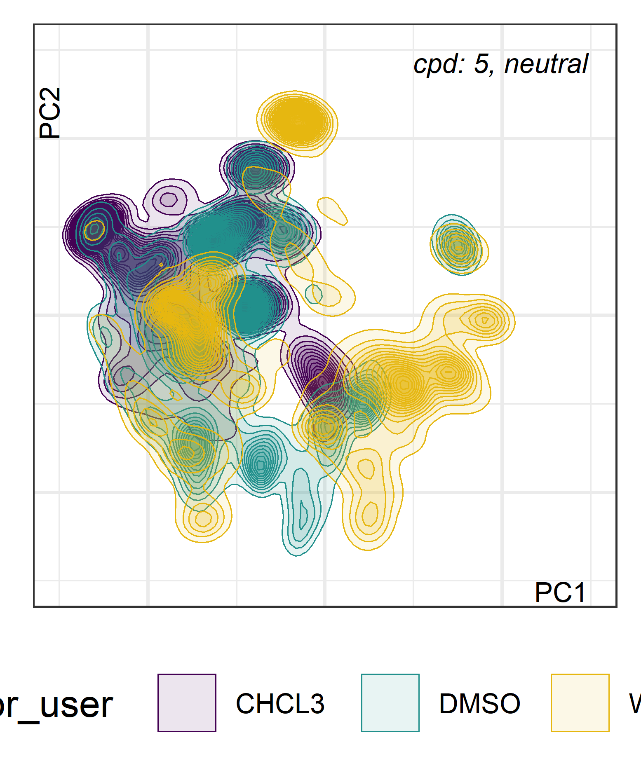
 **6**
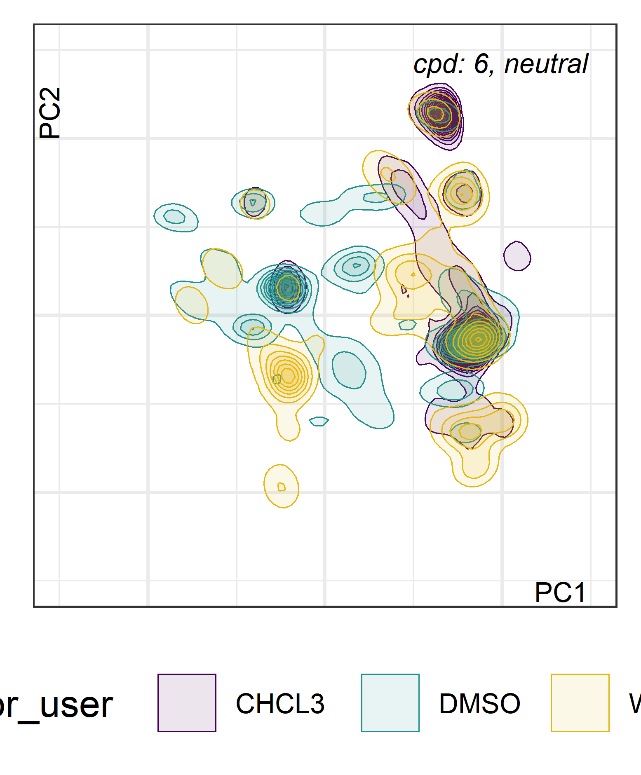


**Figure S8:** Density maps for distributions of accumulated conformers derived from five MD simulations at 300 K with five different starting coordinates in solvent water (orange), DMSO (blue), and CHCl_3_ (pink), for neutral compounds **1** to **6**.

**1:**
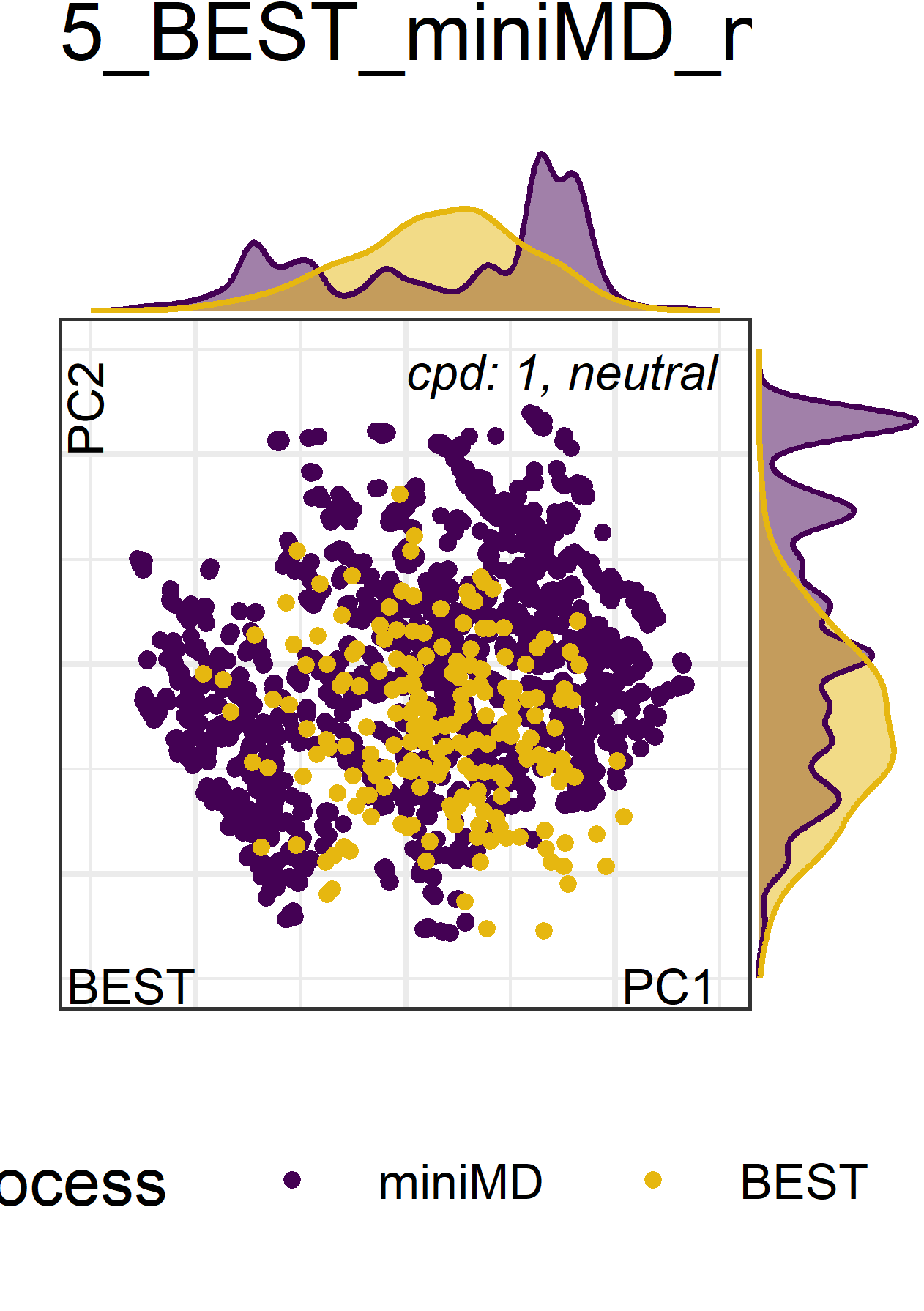

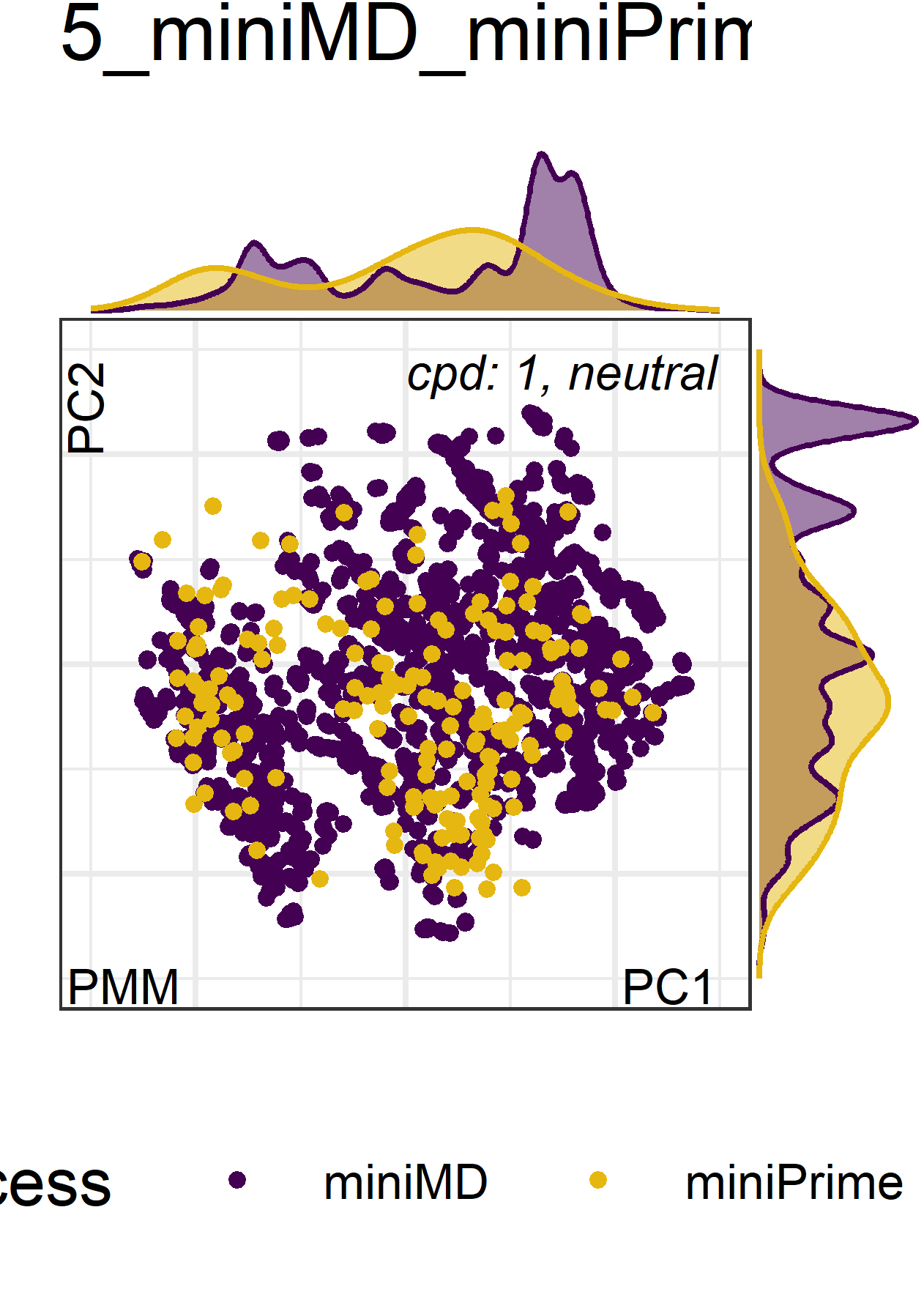

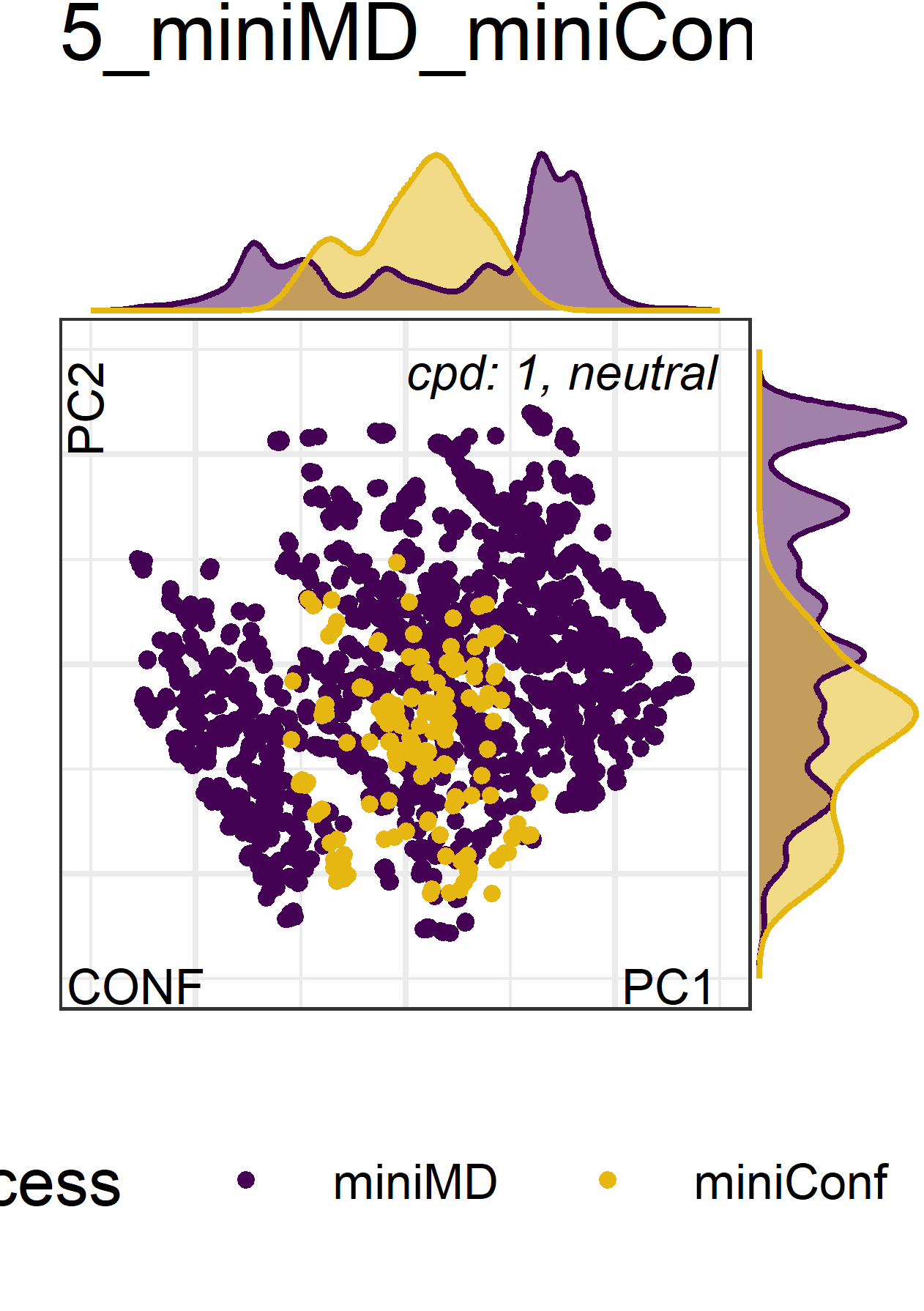
**2:**
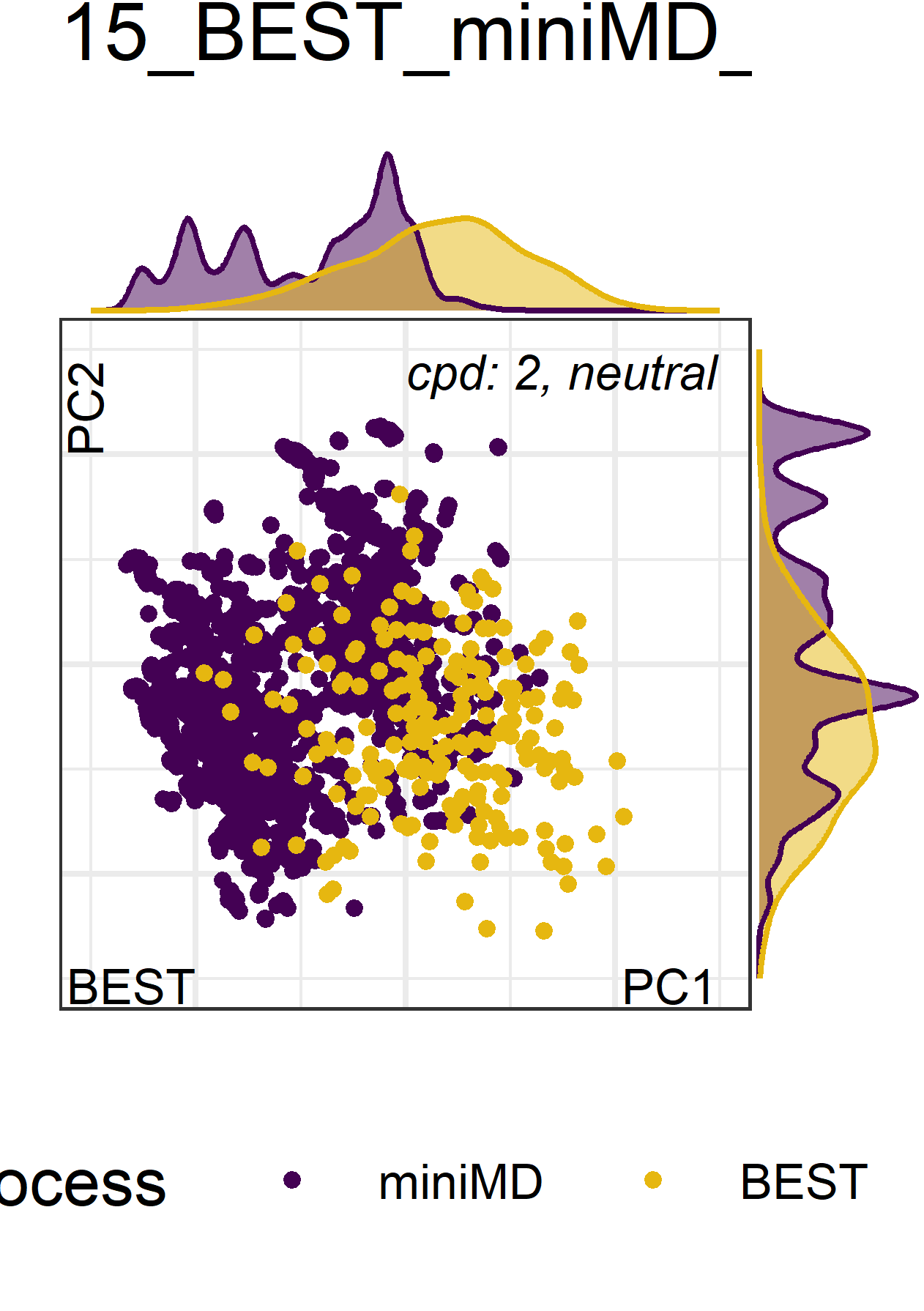

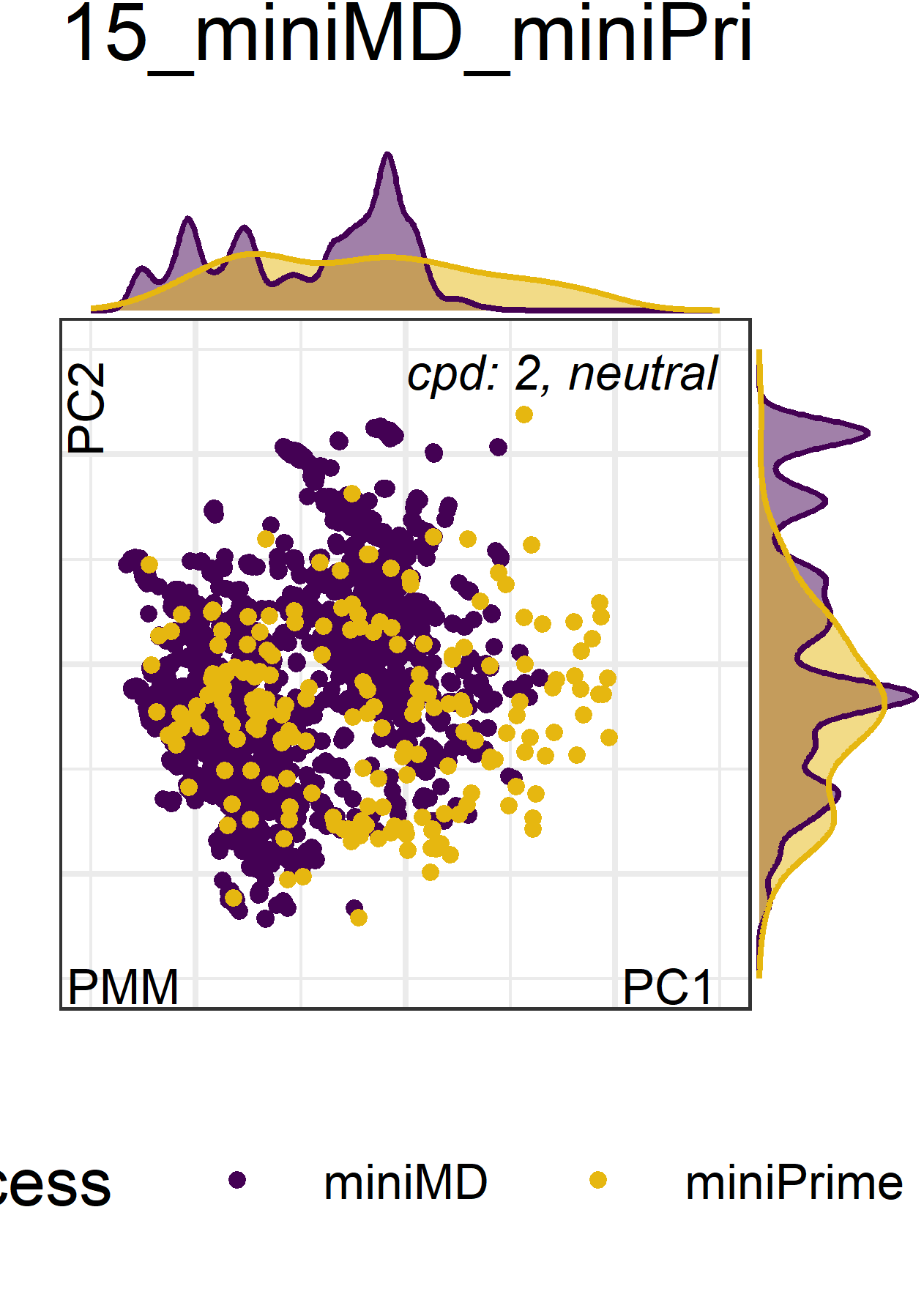

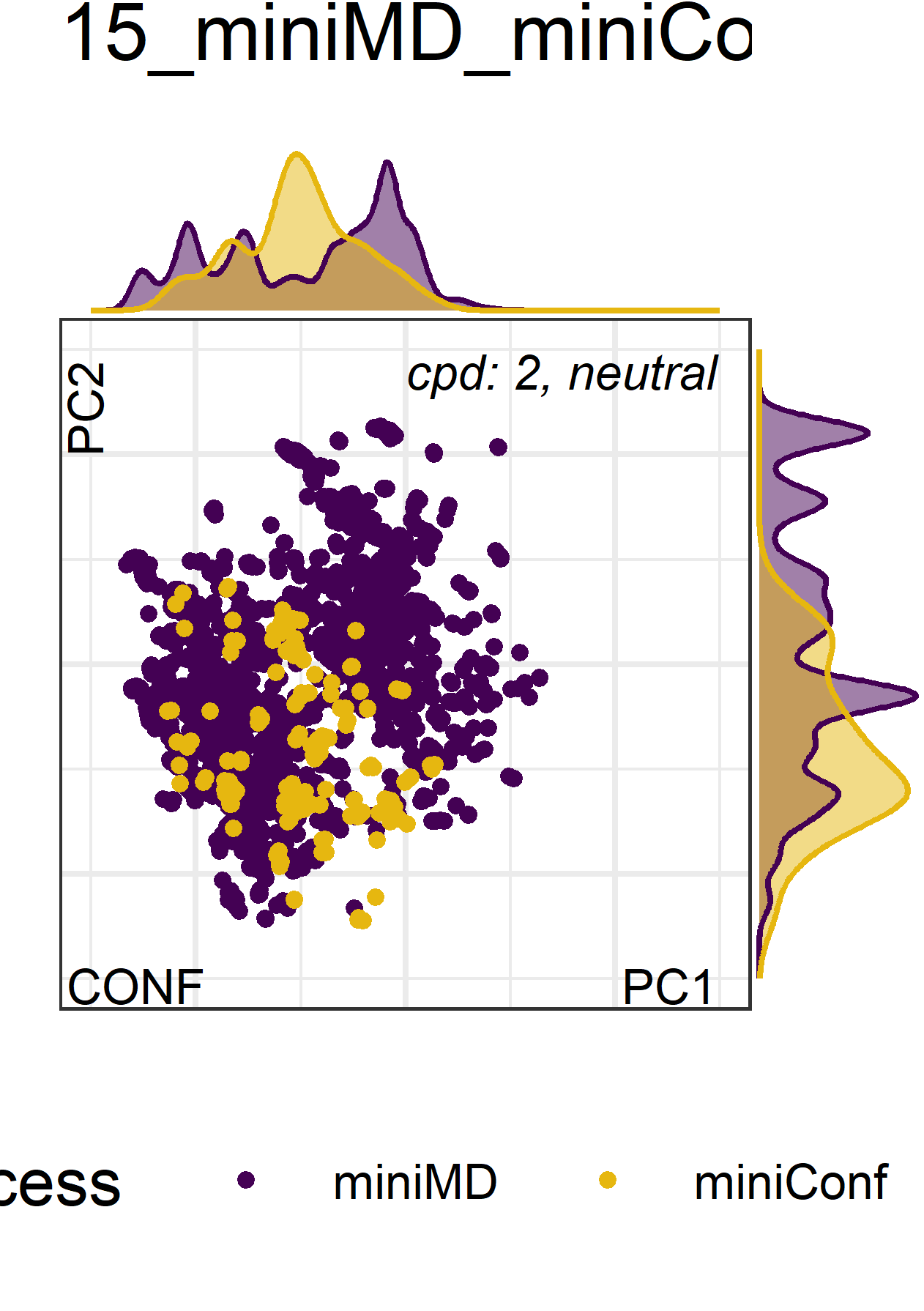
**3:**
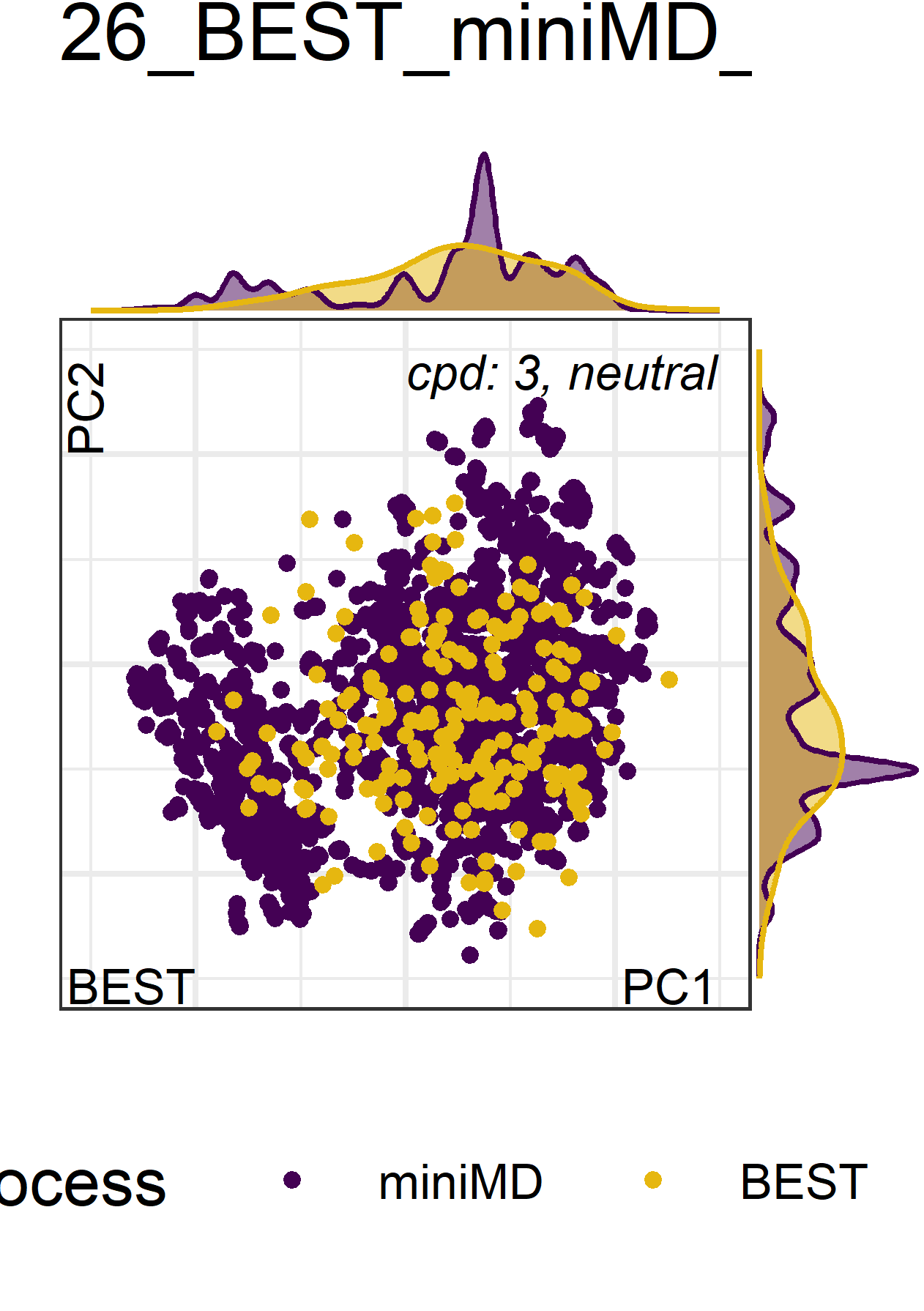

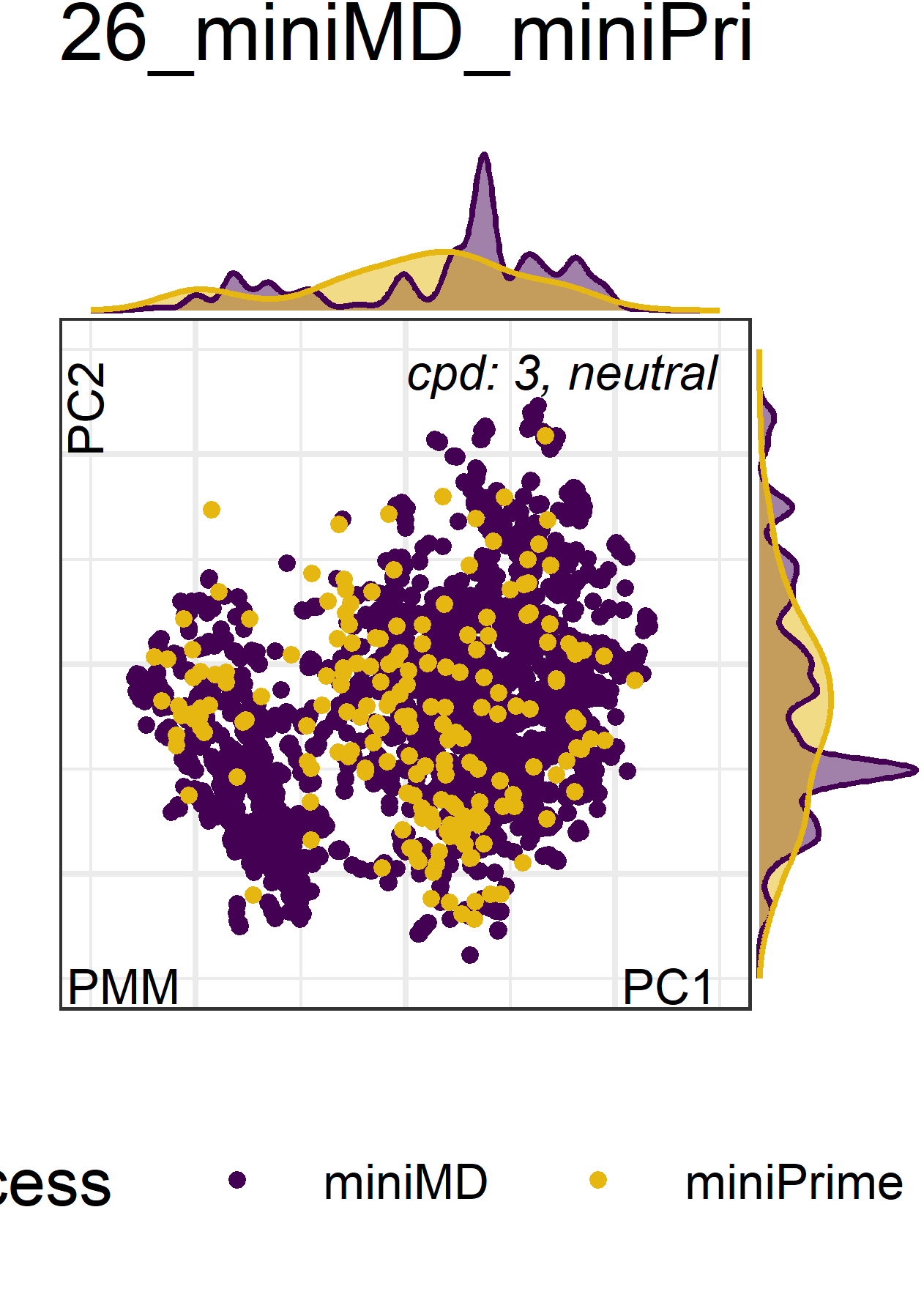

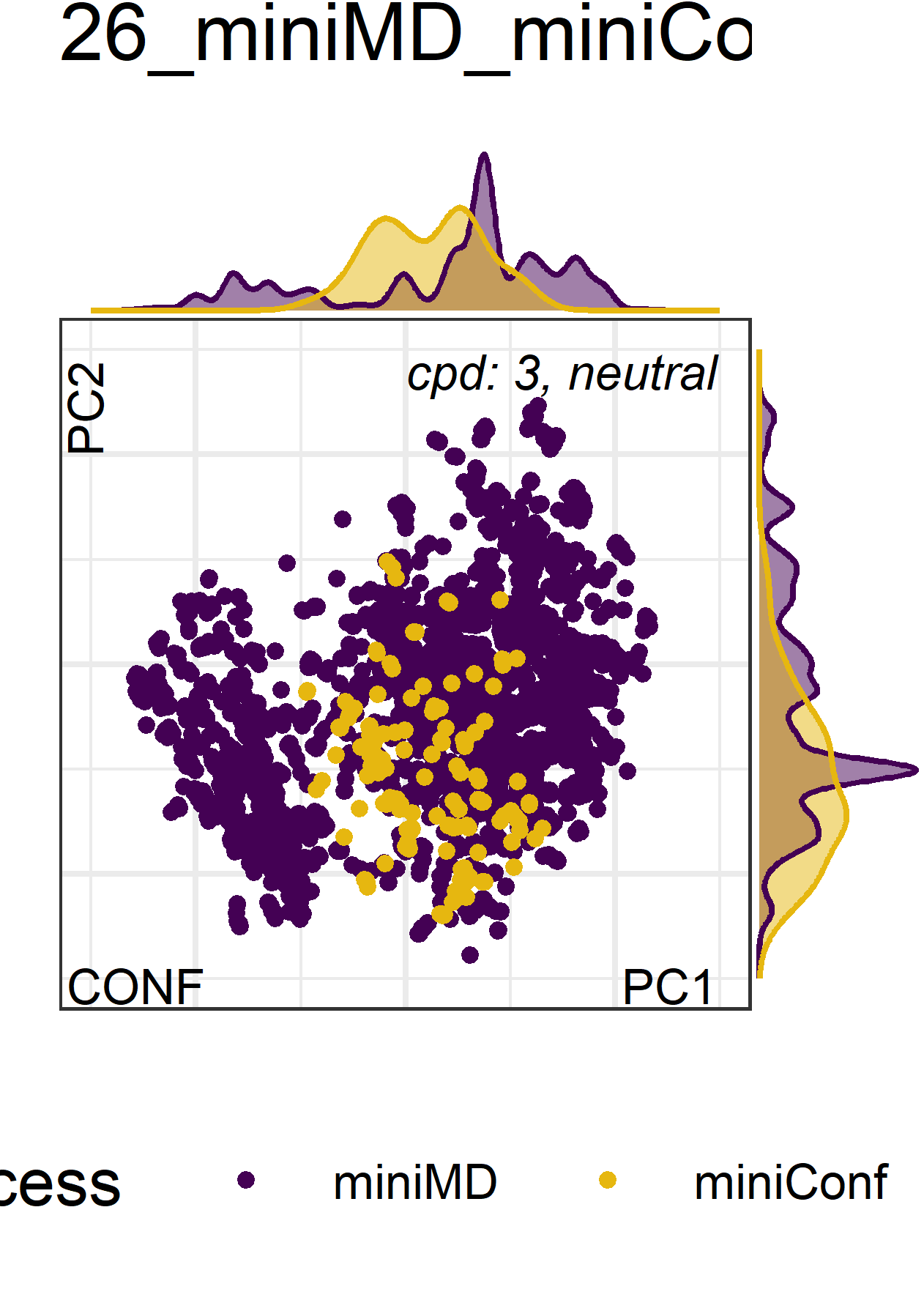
**4:**
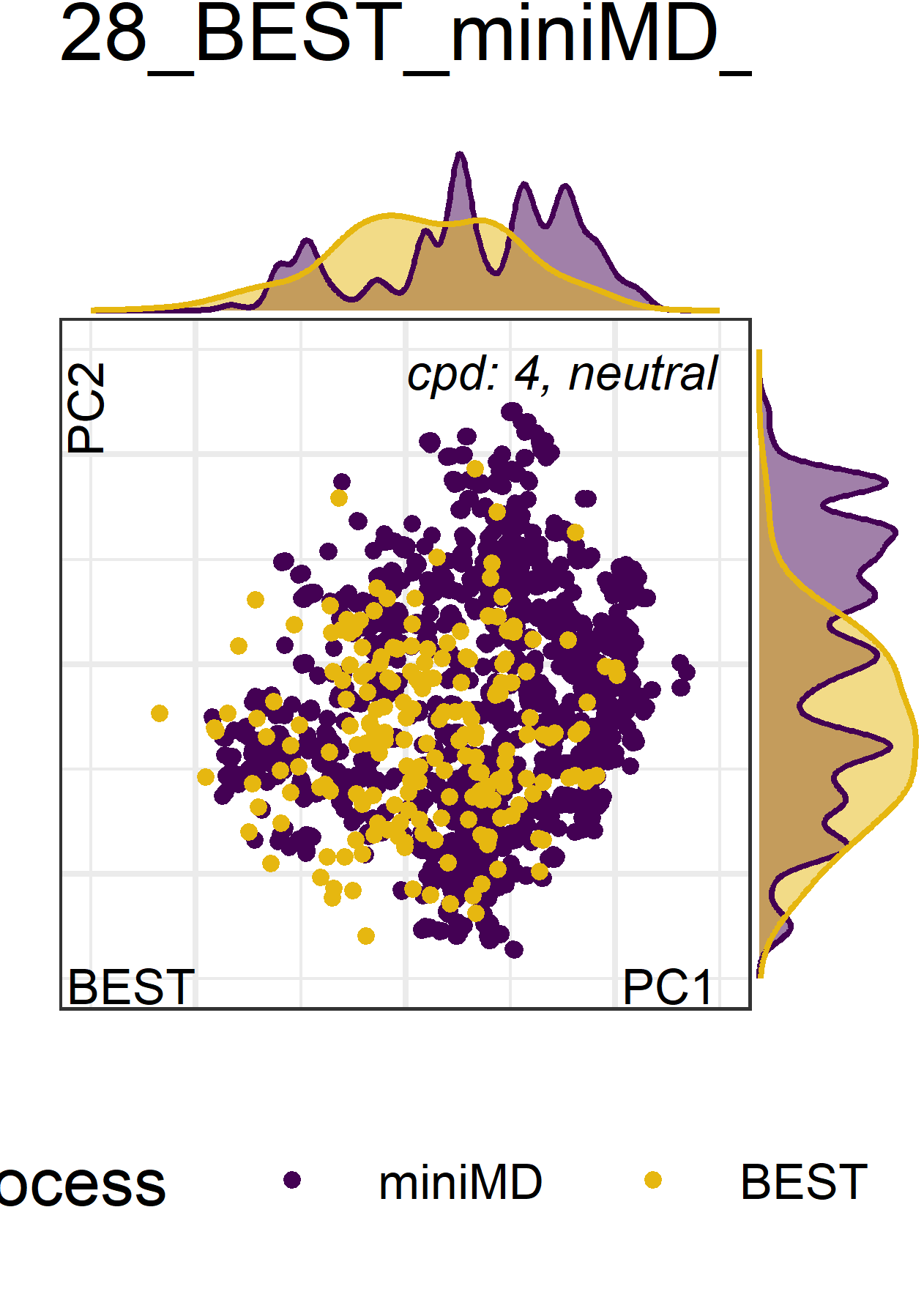

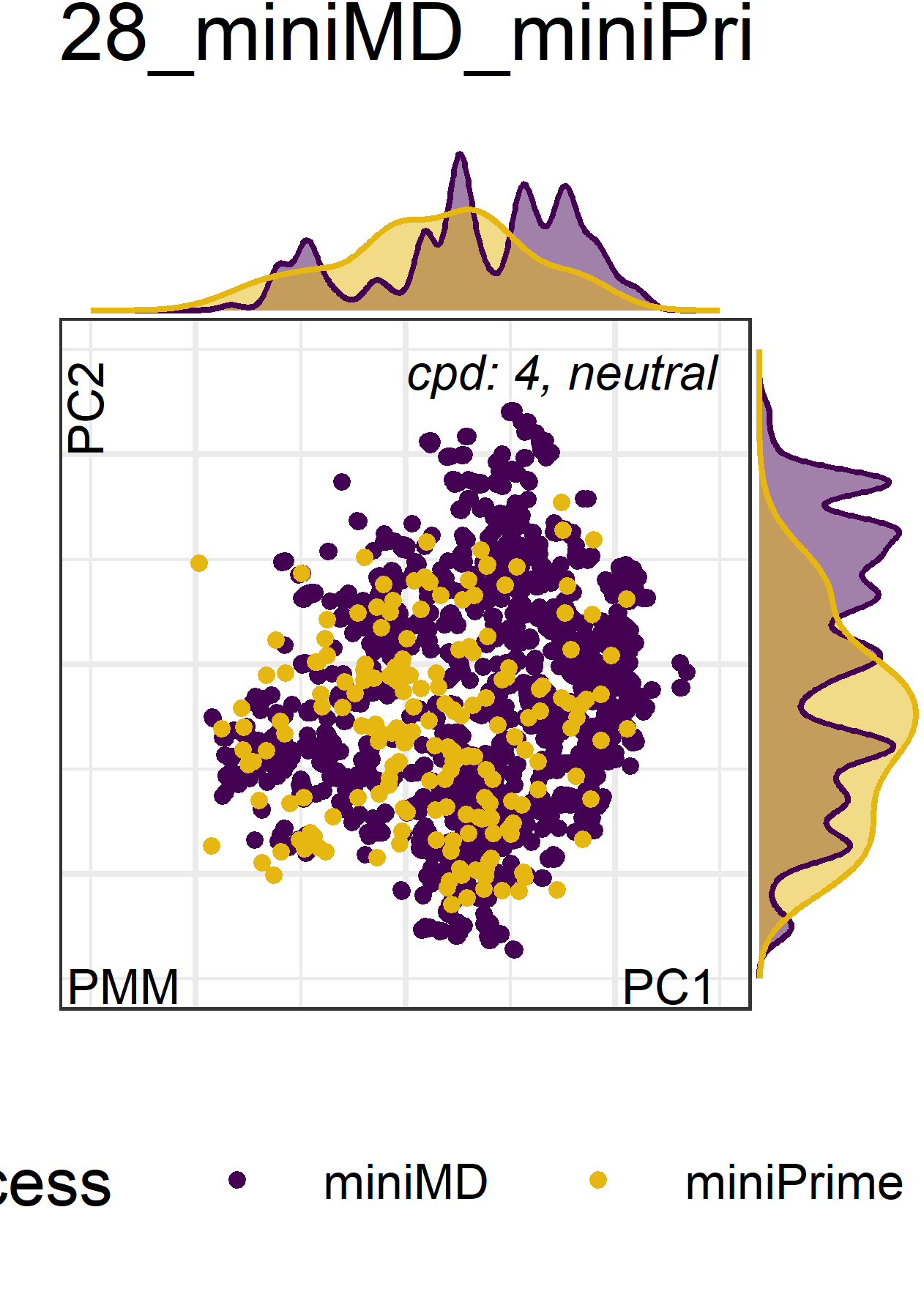

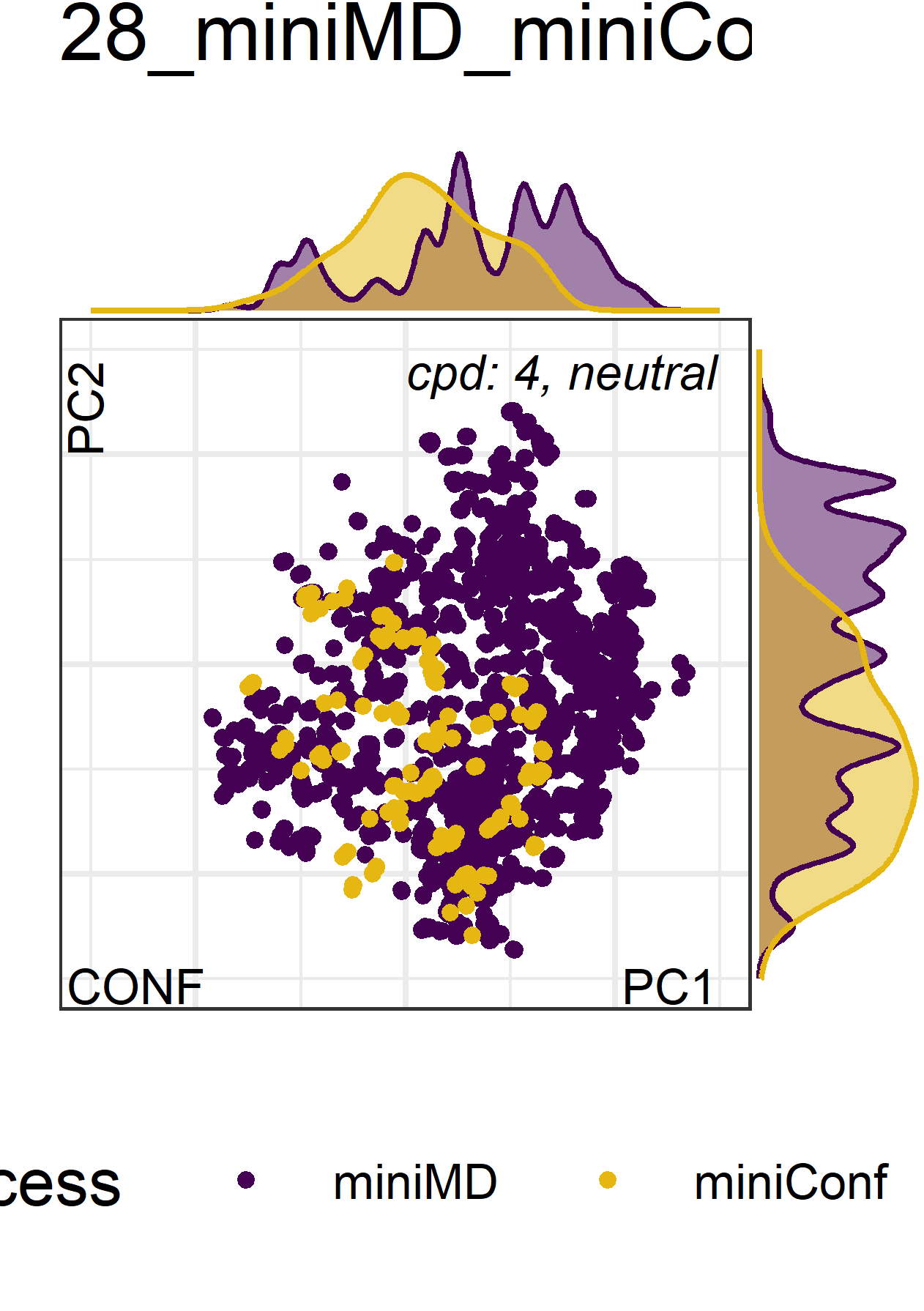
**5:**
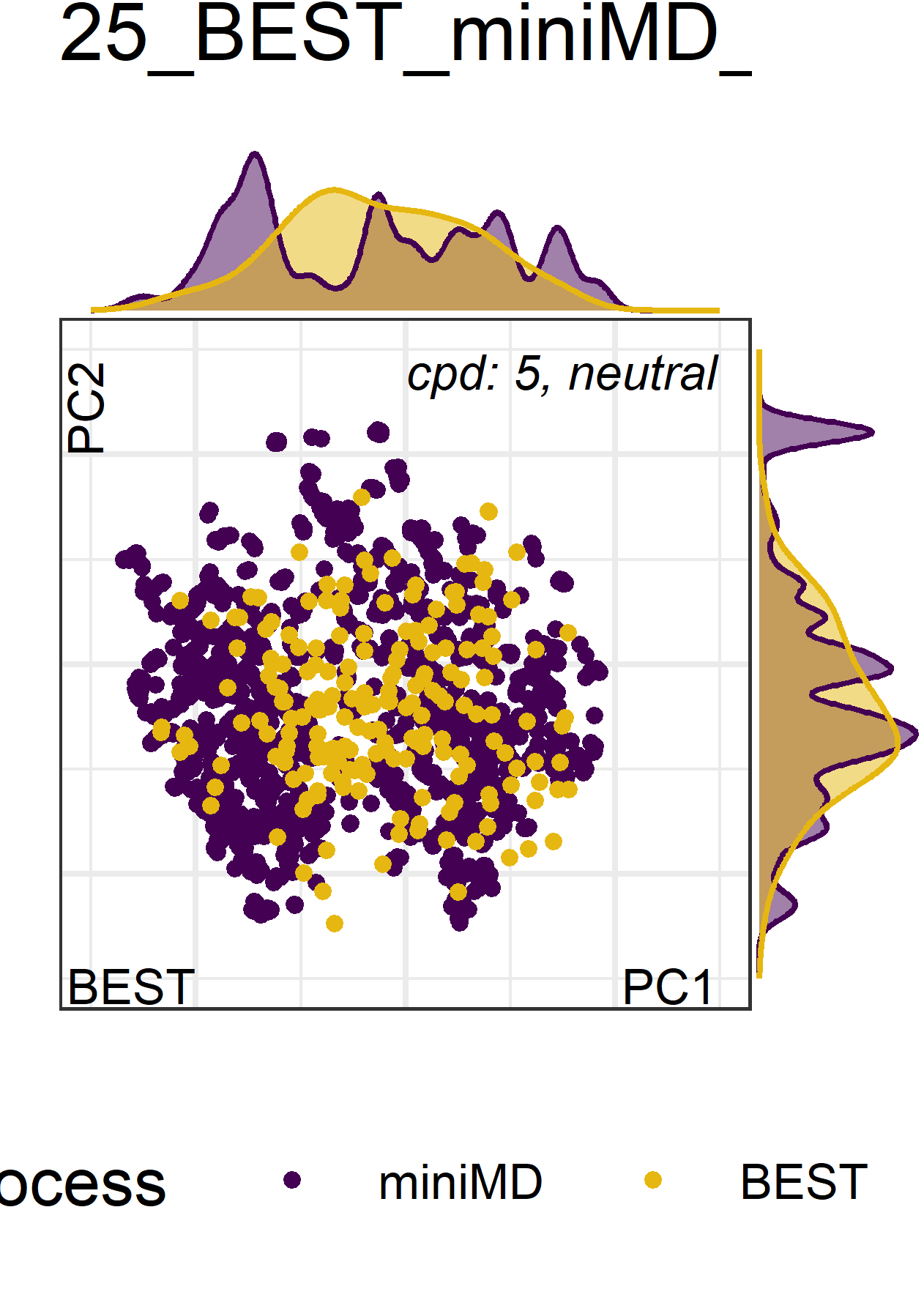

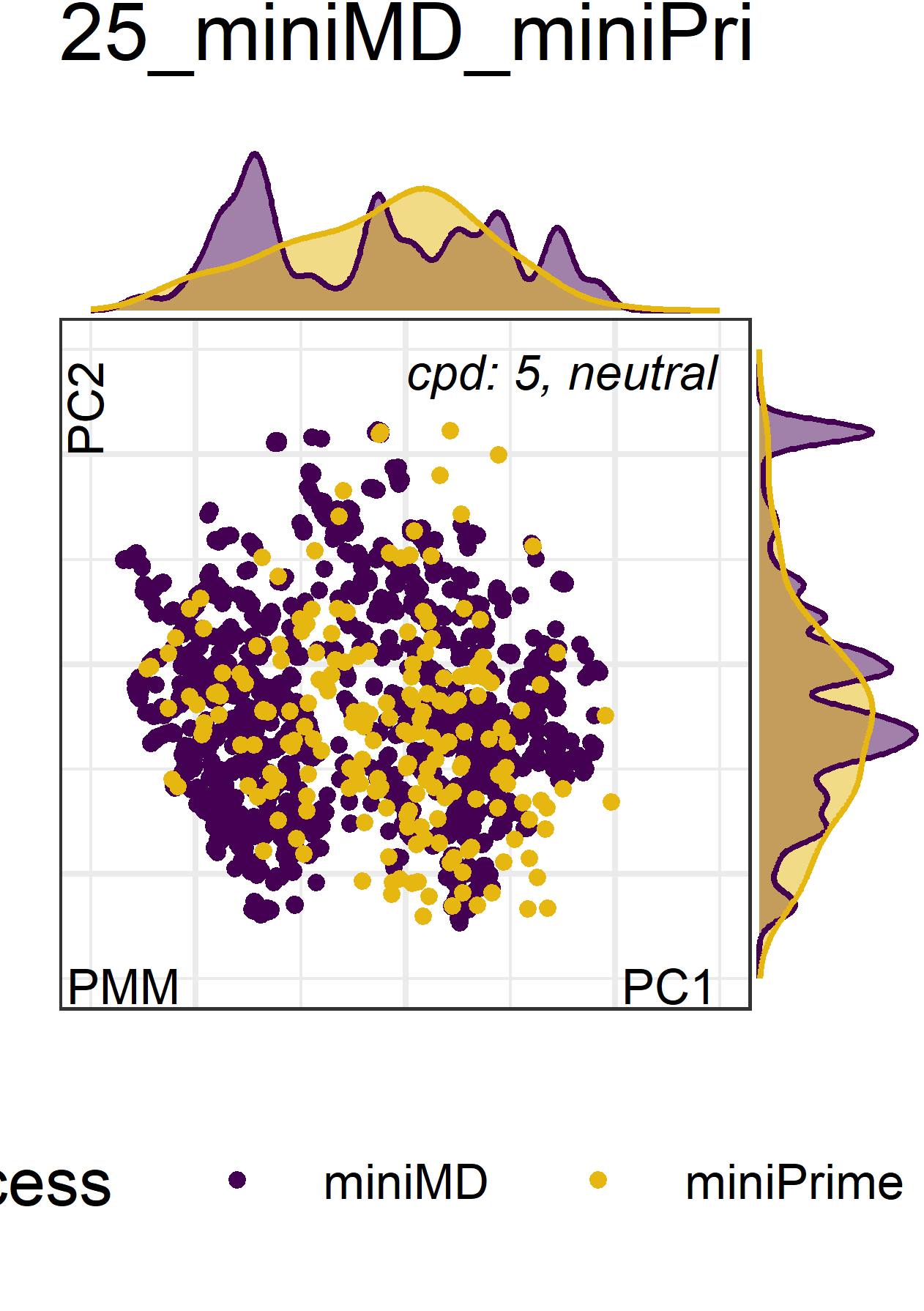

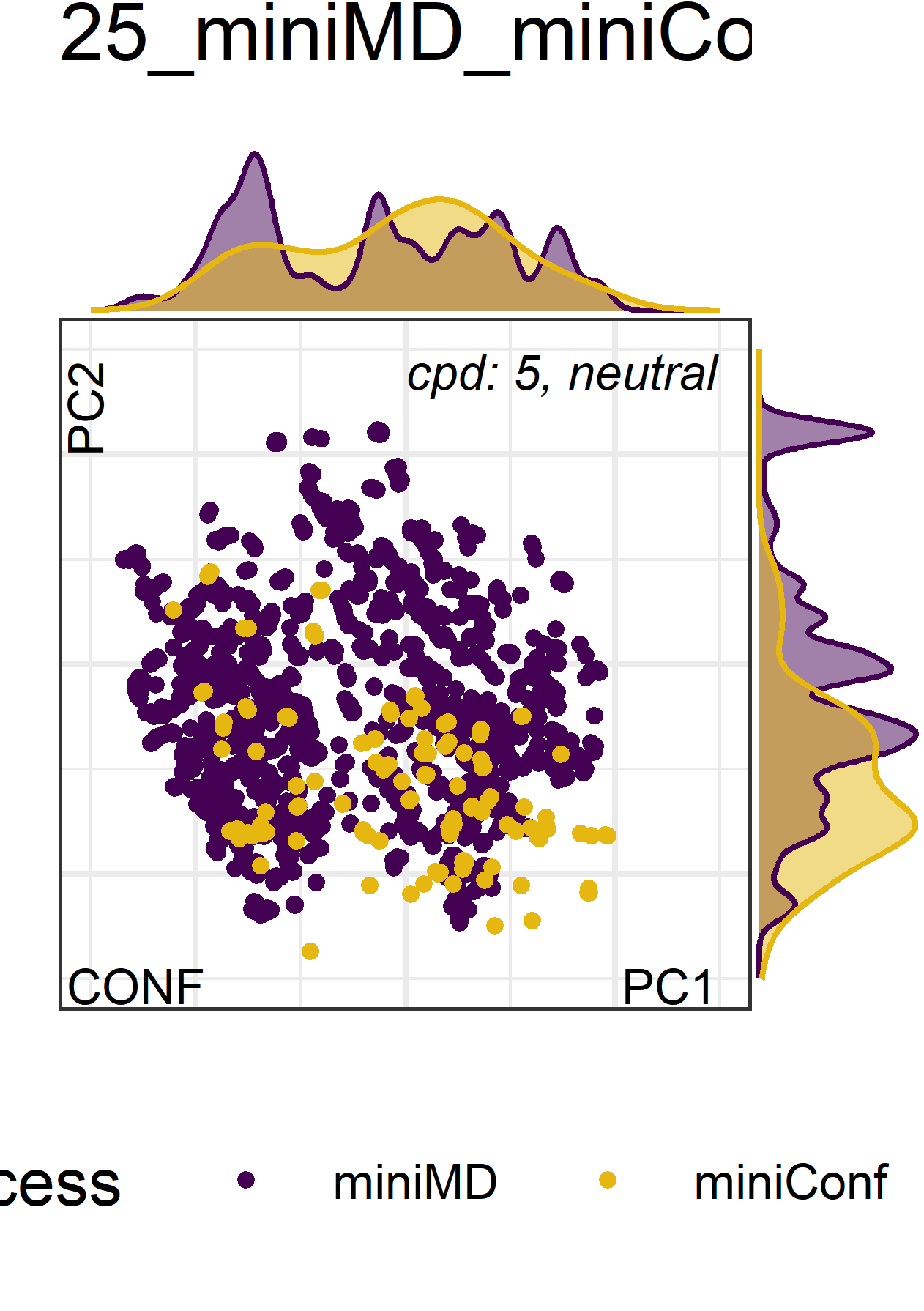
**6:**
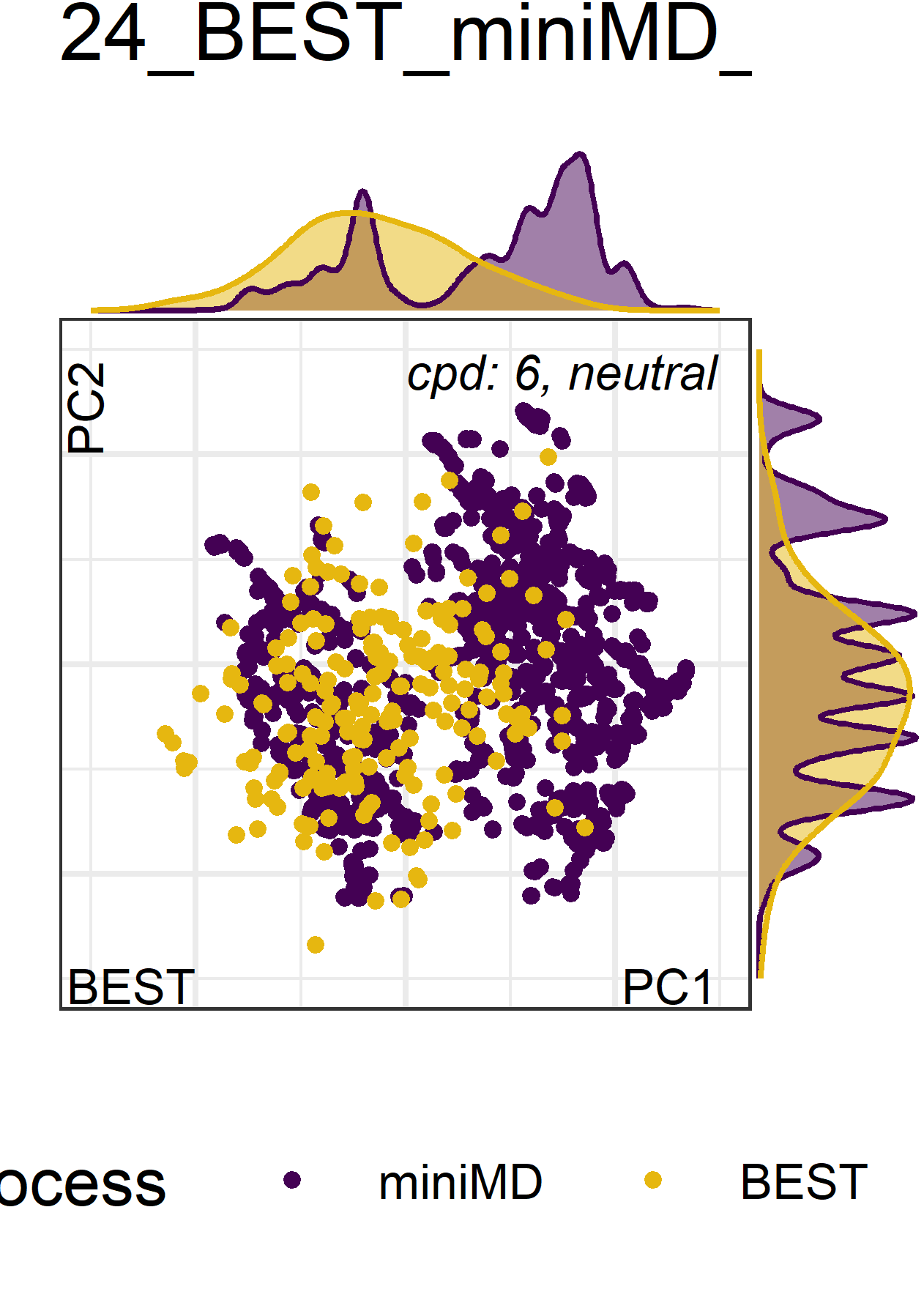

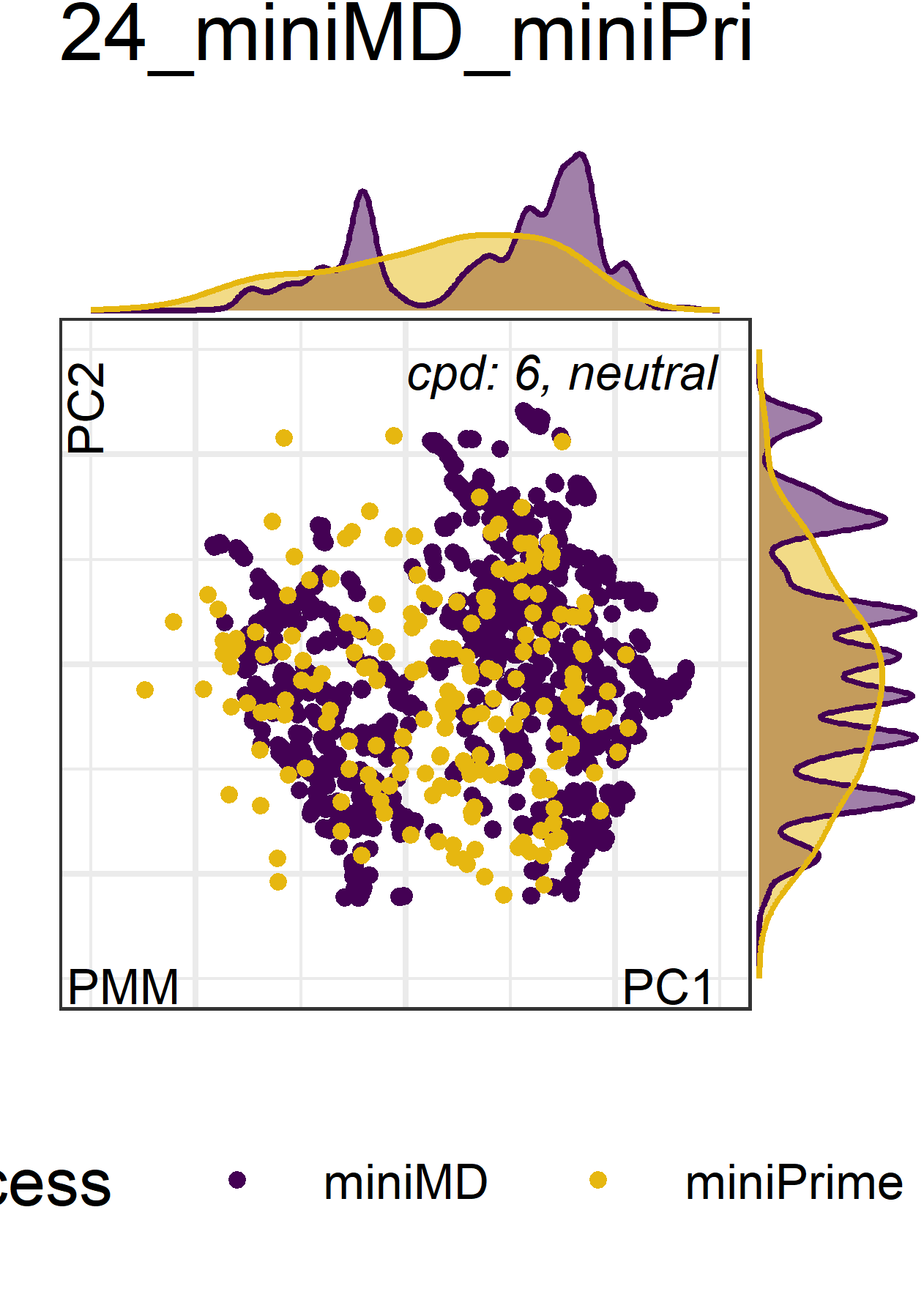

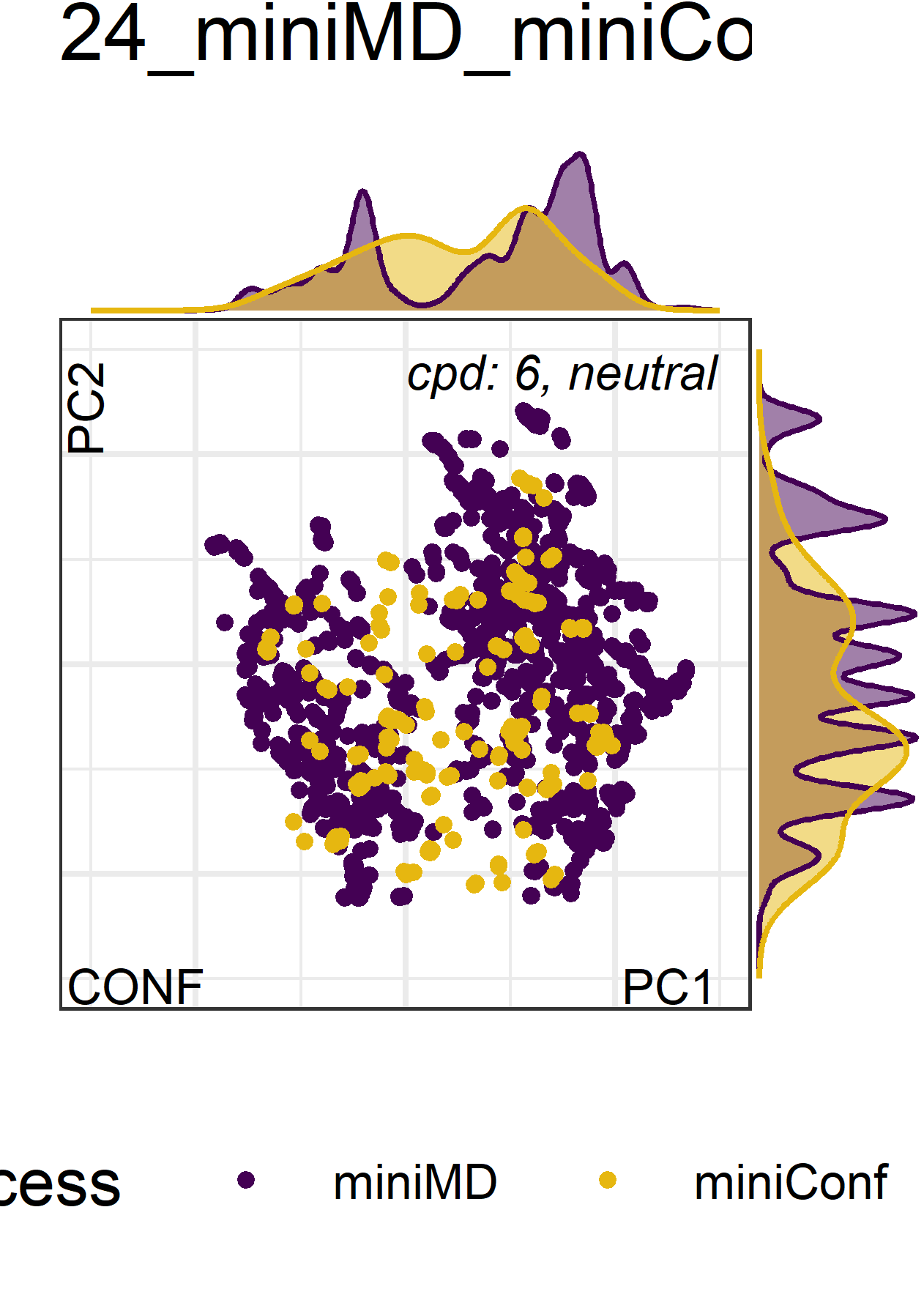
**7:**
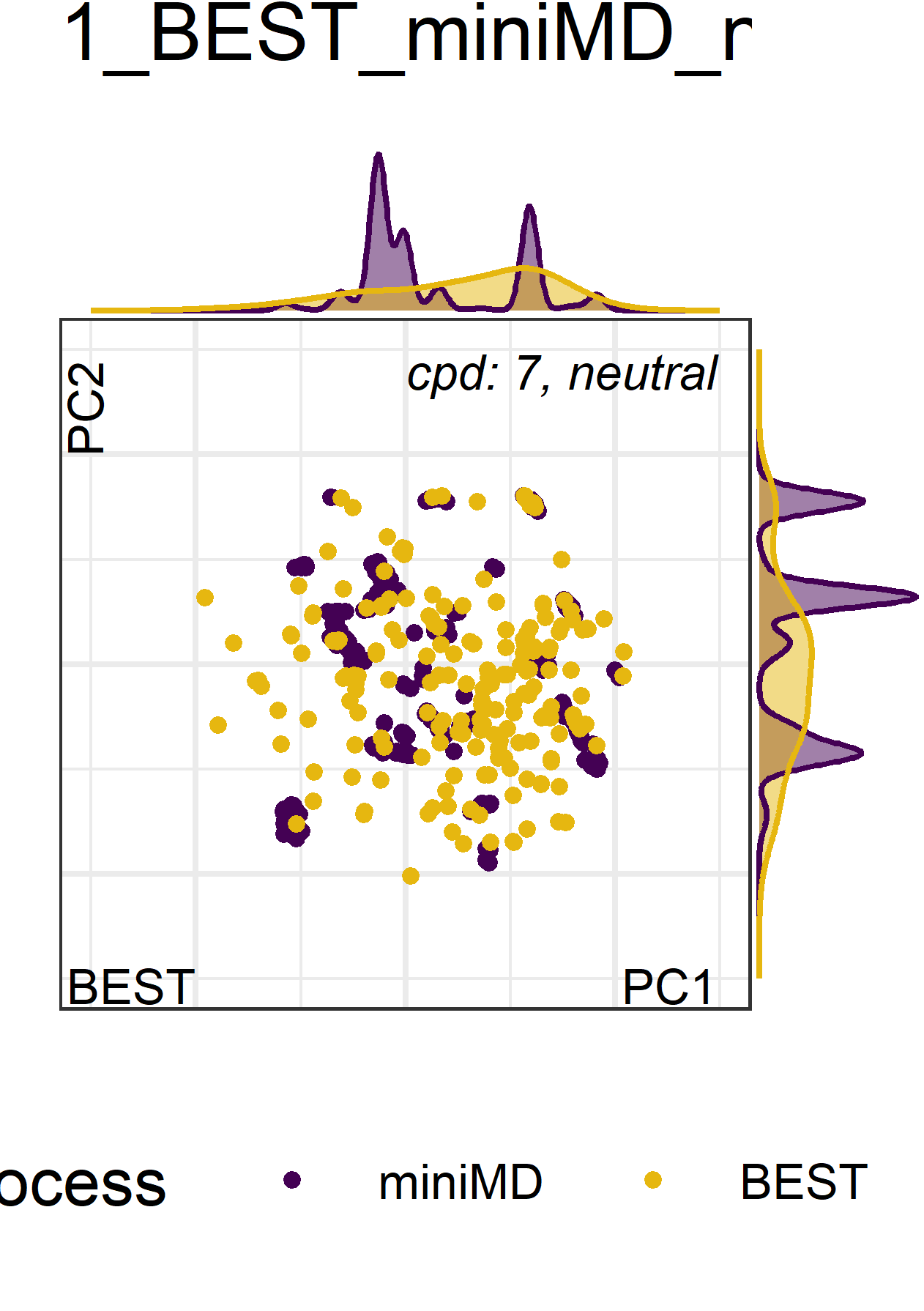

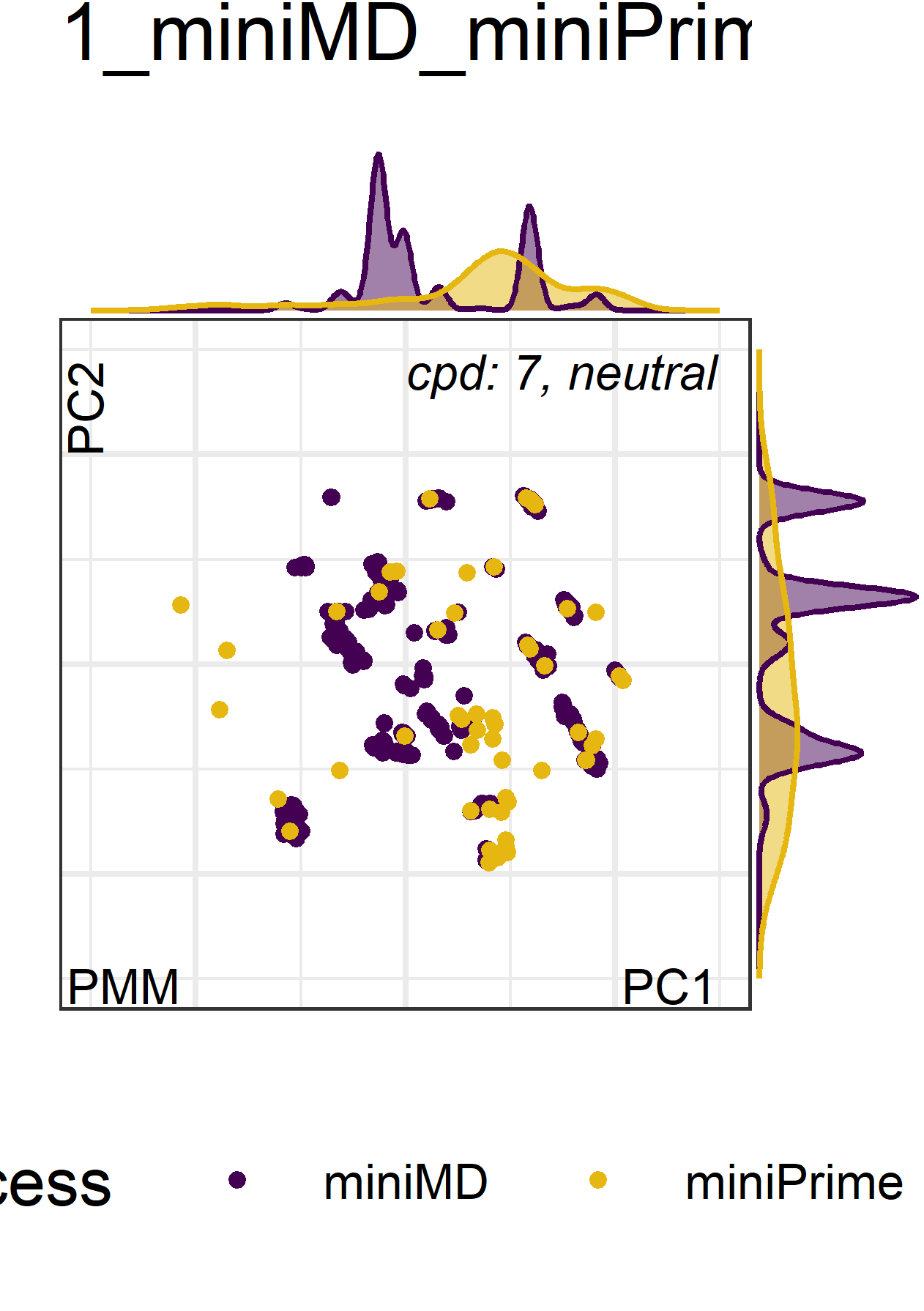

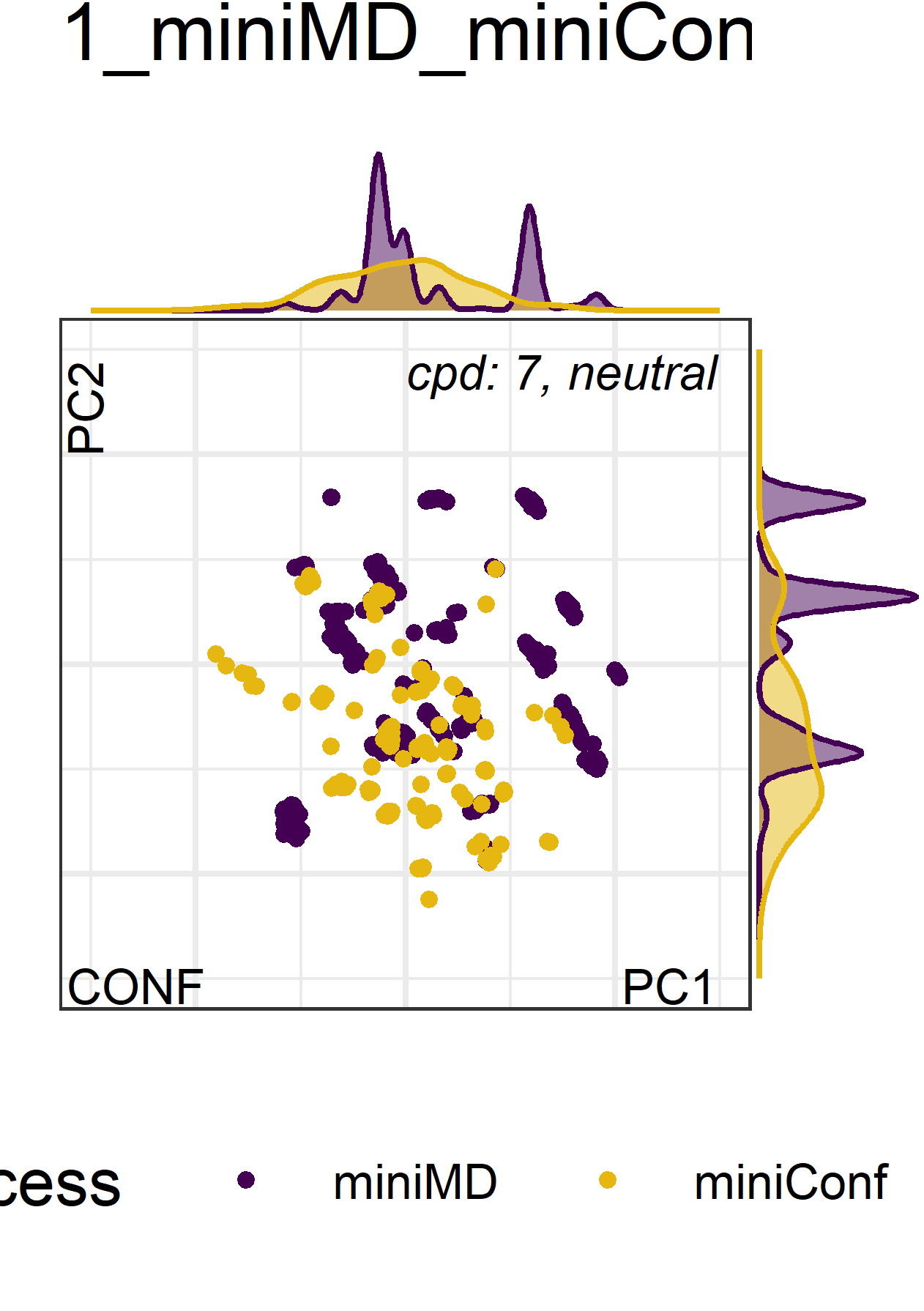


**Figure S9:** Maps of latent torsional space accessed by post-minimized MD snapshots (5 starting conformers, 300 K 100 ns each) for neutral compounds in SPC water in purple, overlaid with conformers created by BEST (left column), PMM (middle) and CONF (right) in orange for macrocycles **1** to **7** (see row labels).

**1:**
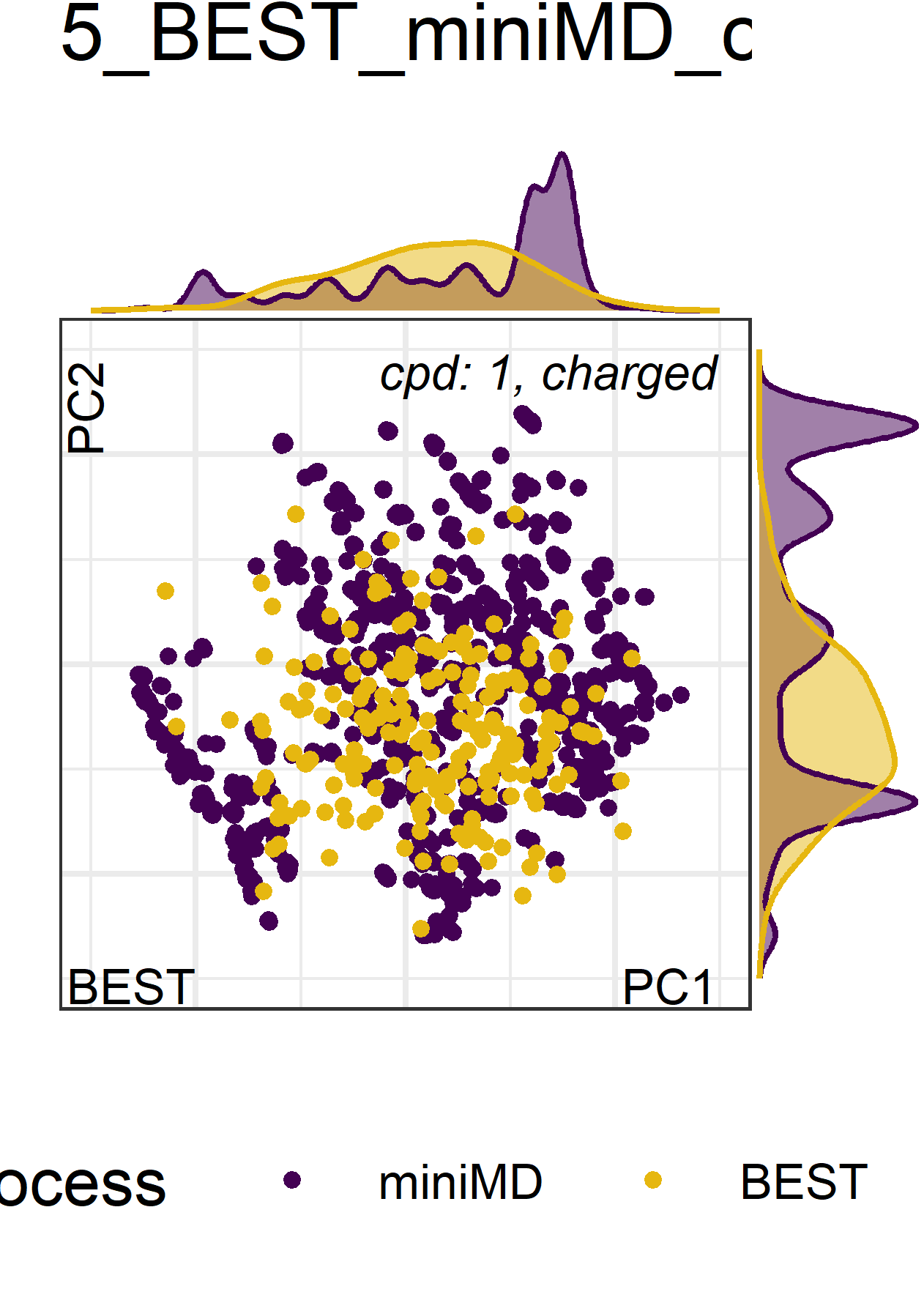

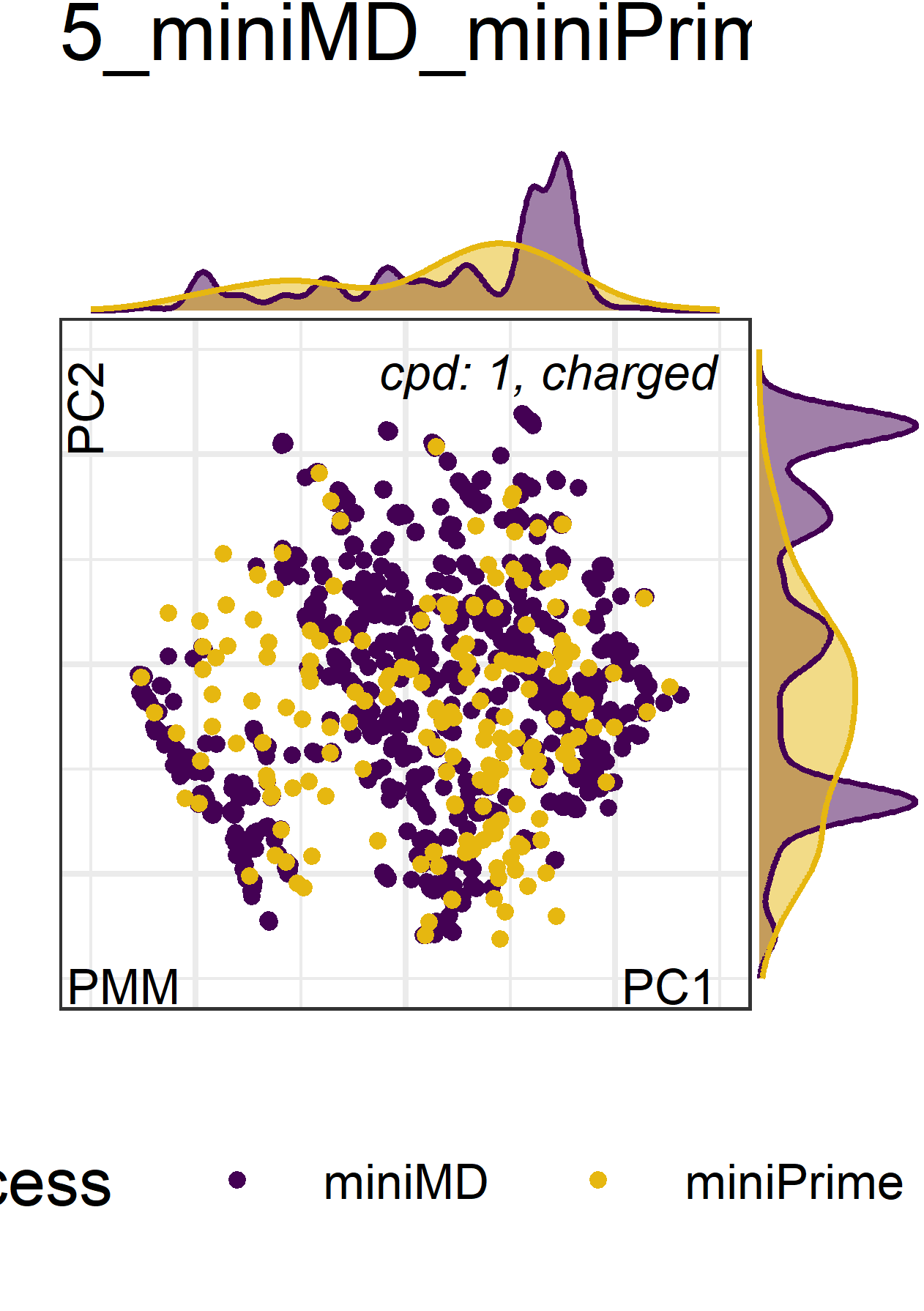

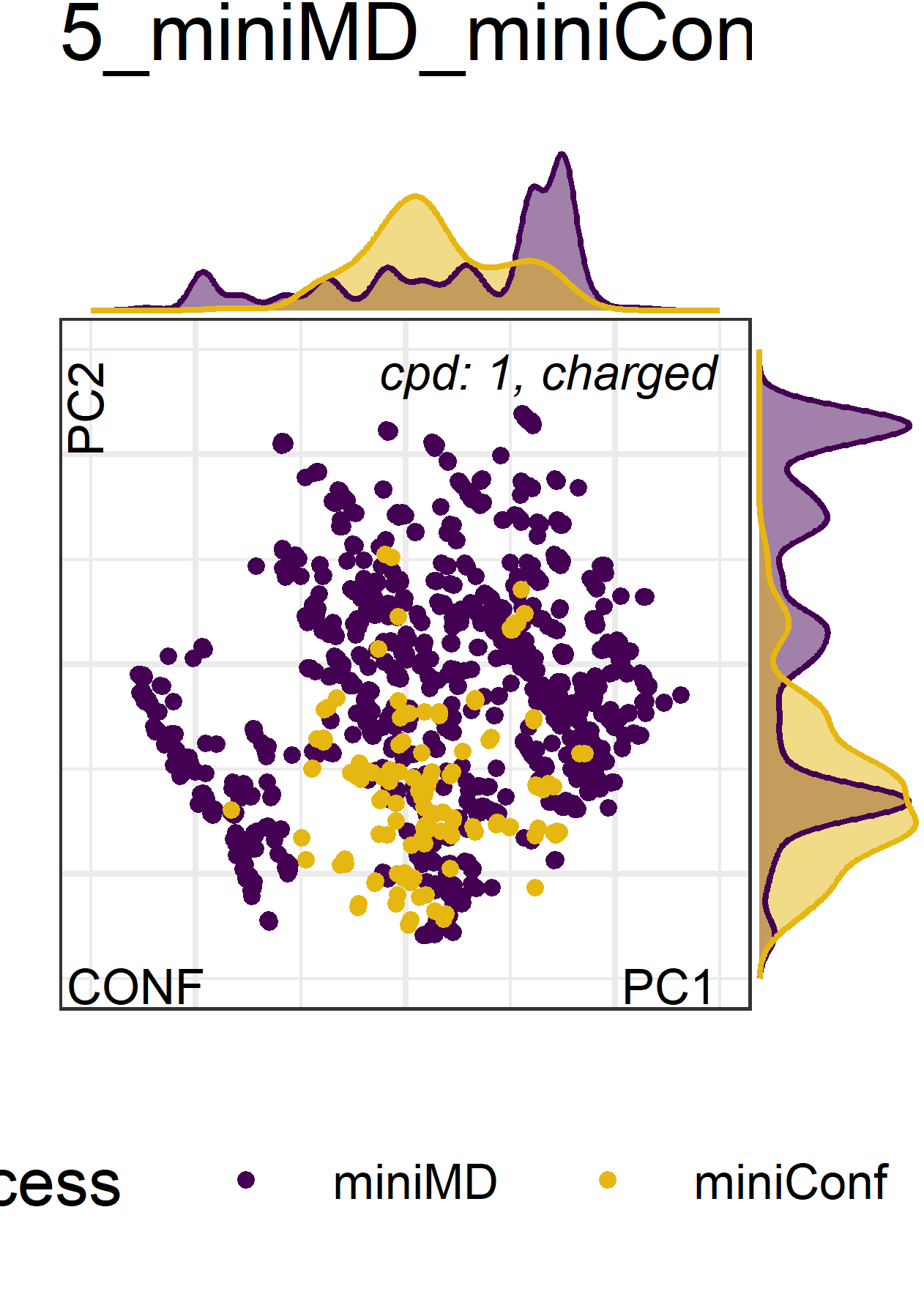
**2:**
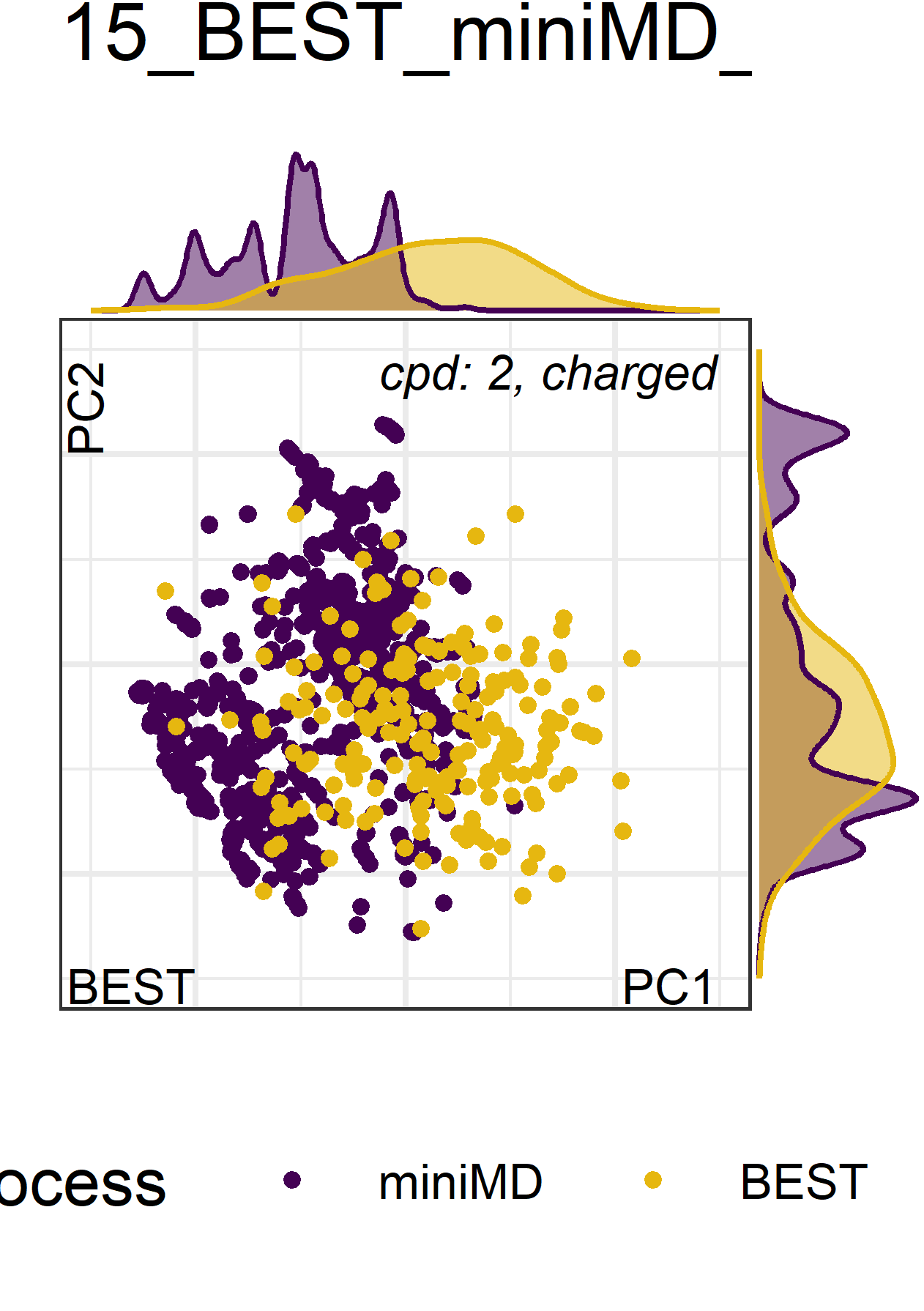

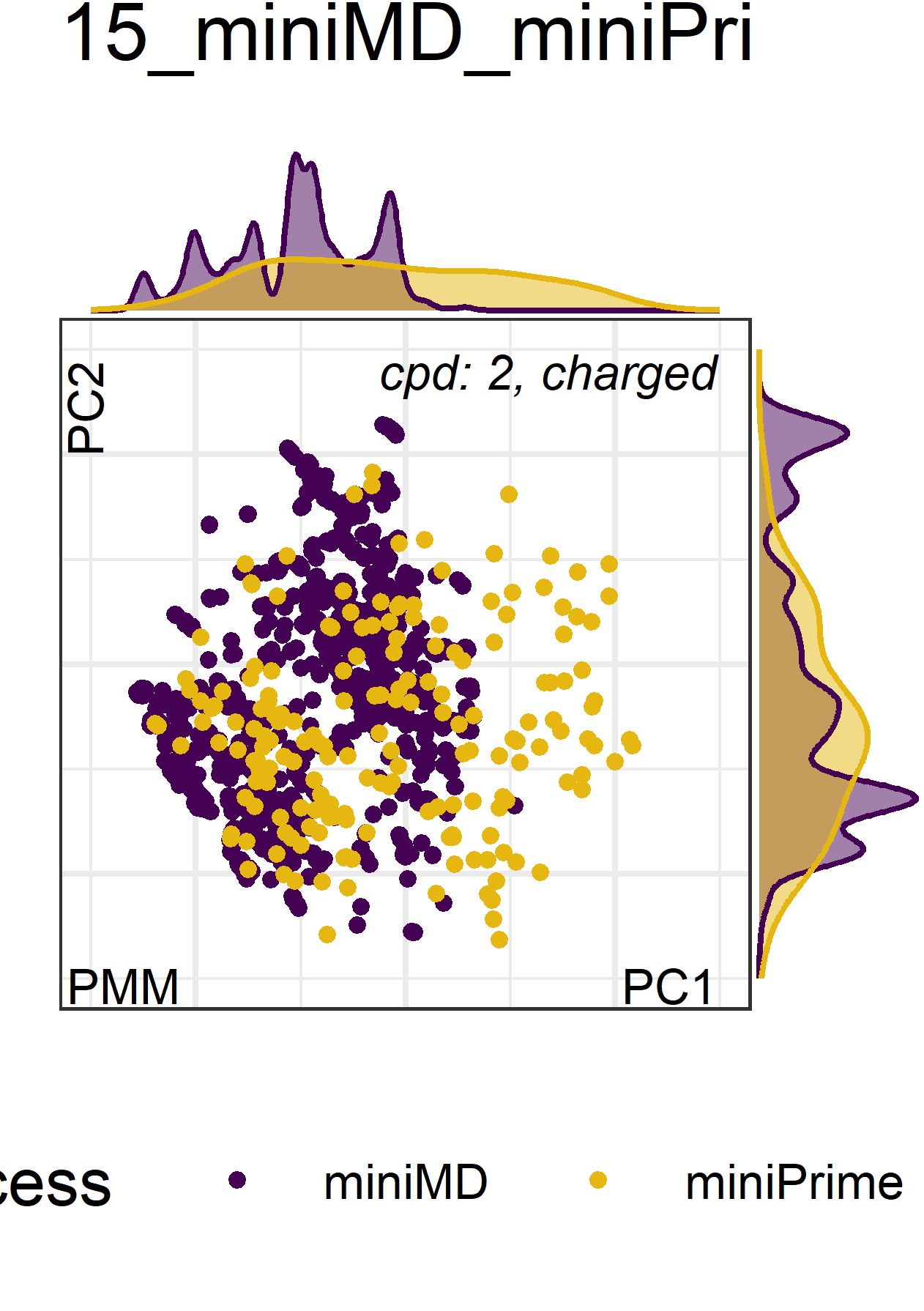

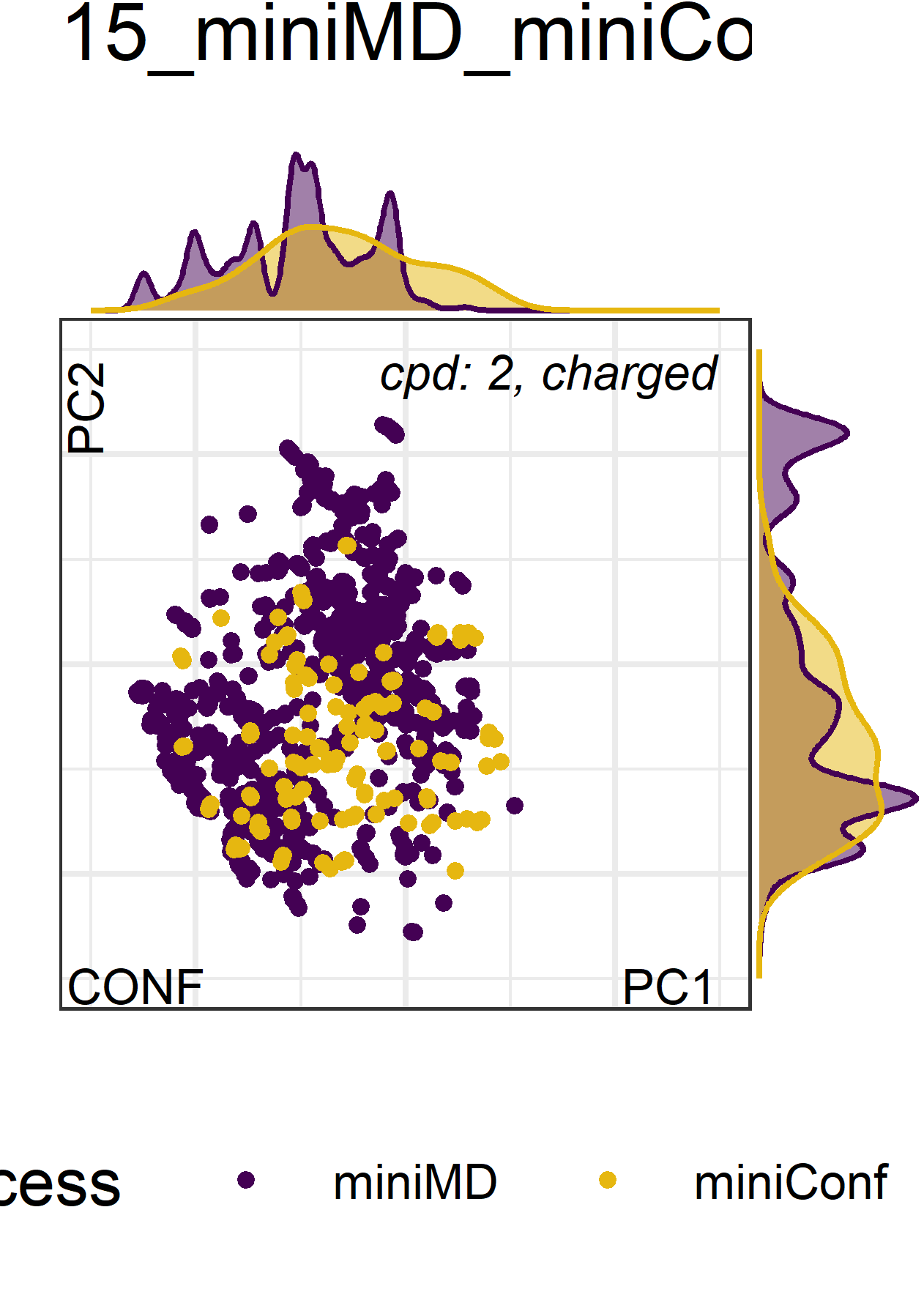
**4:**
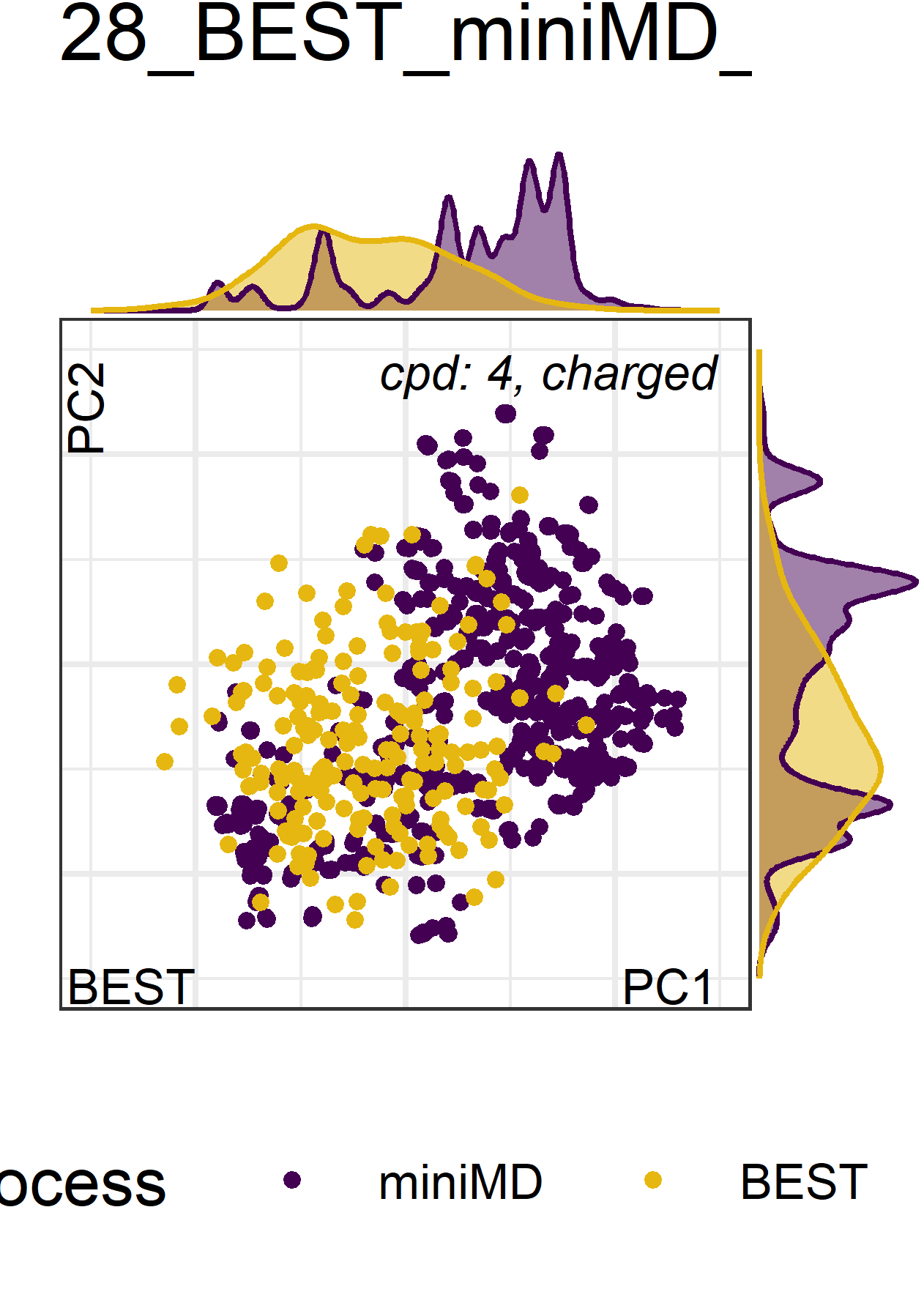

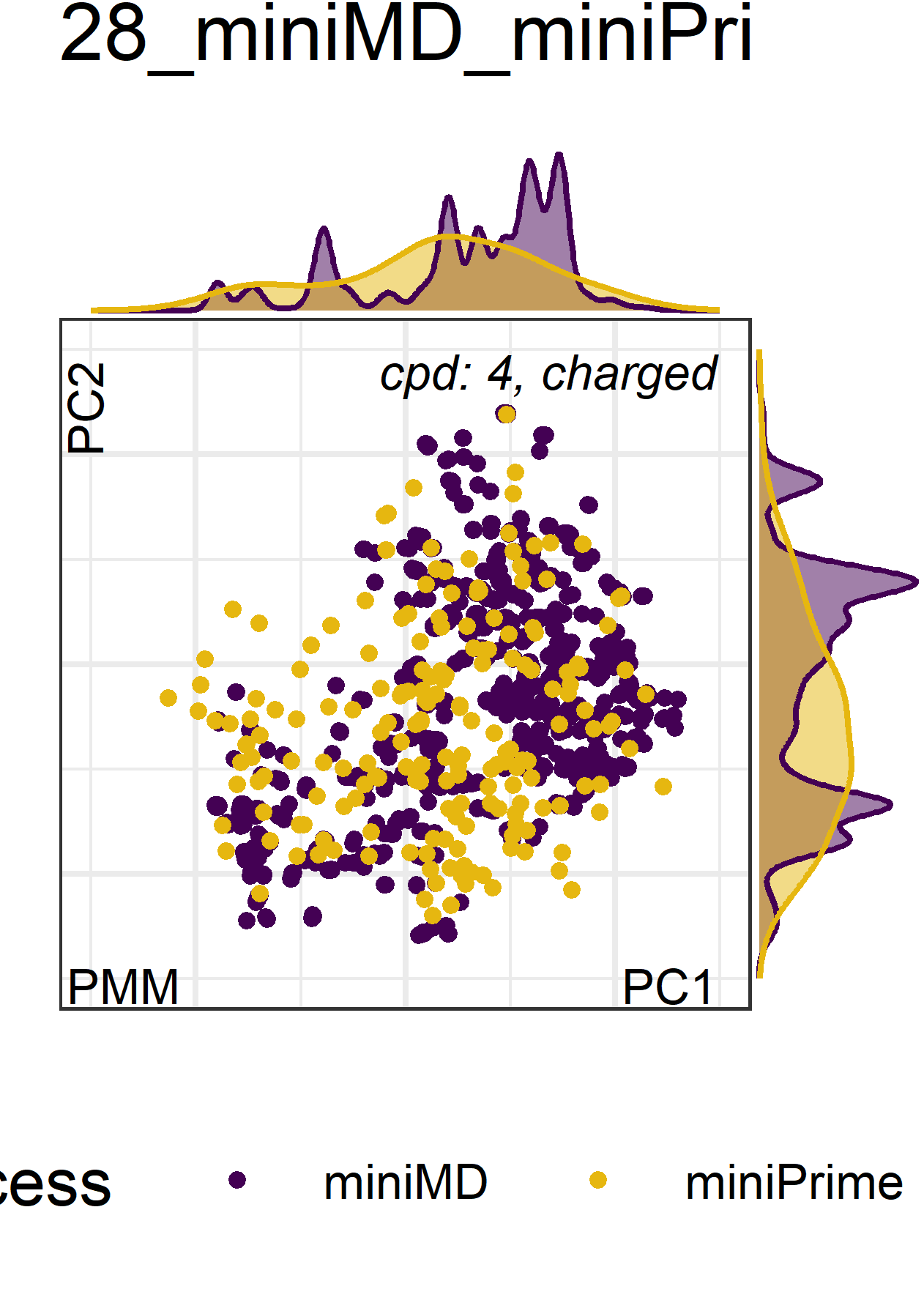

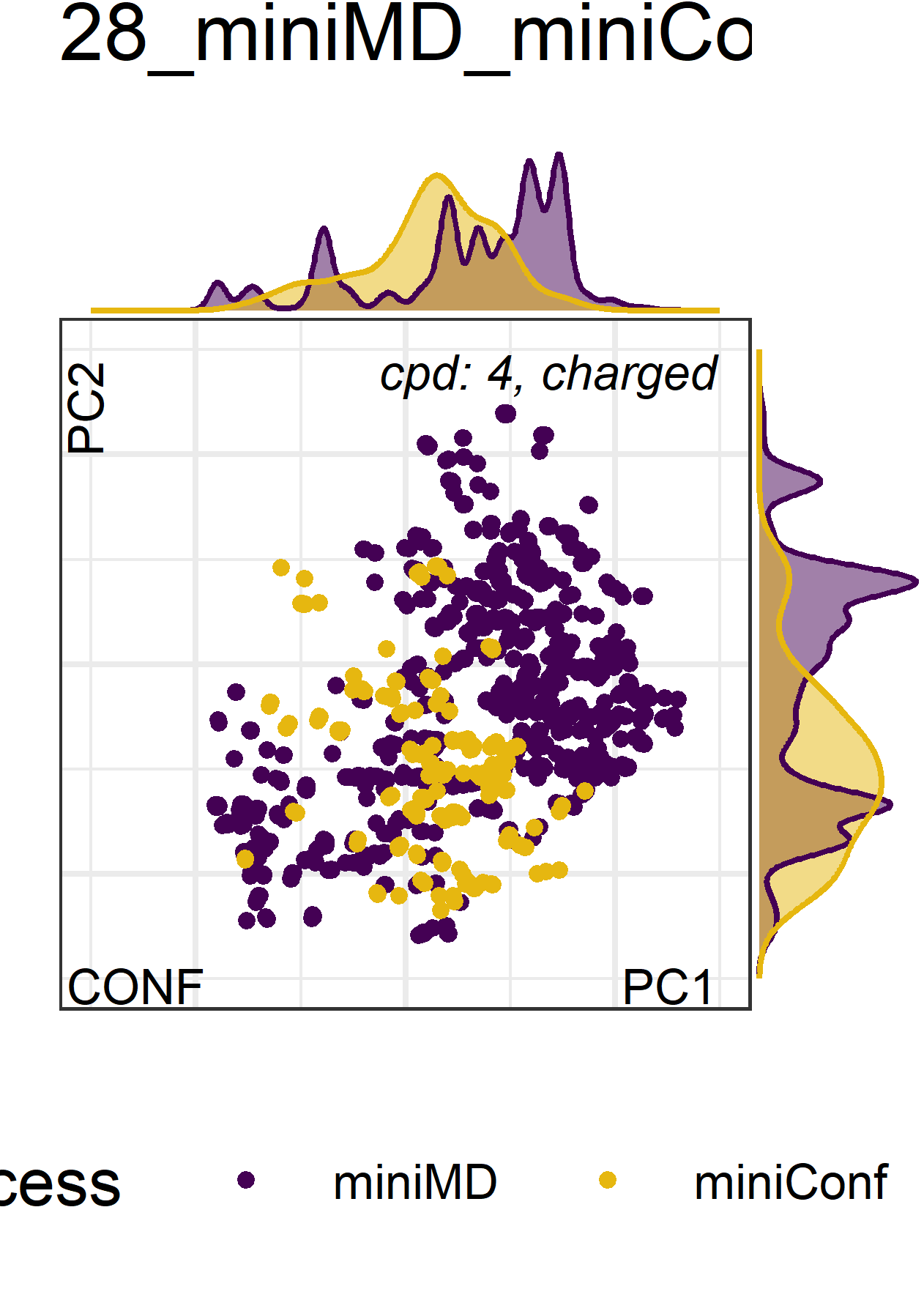
**5:**
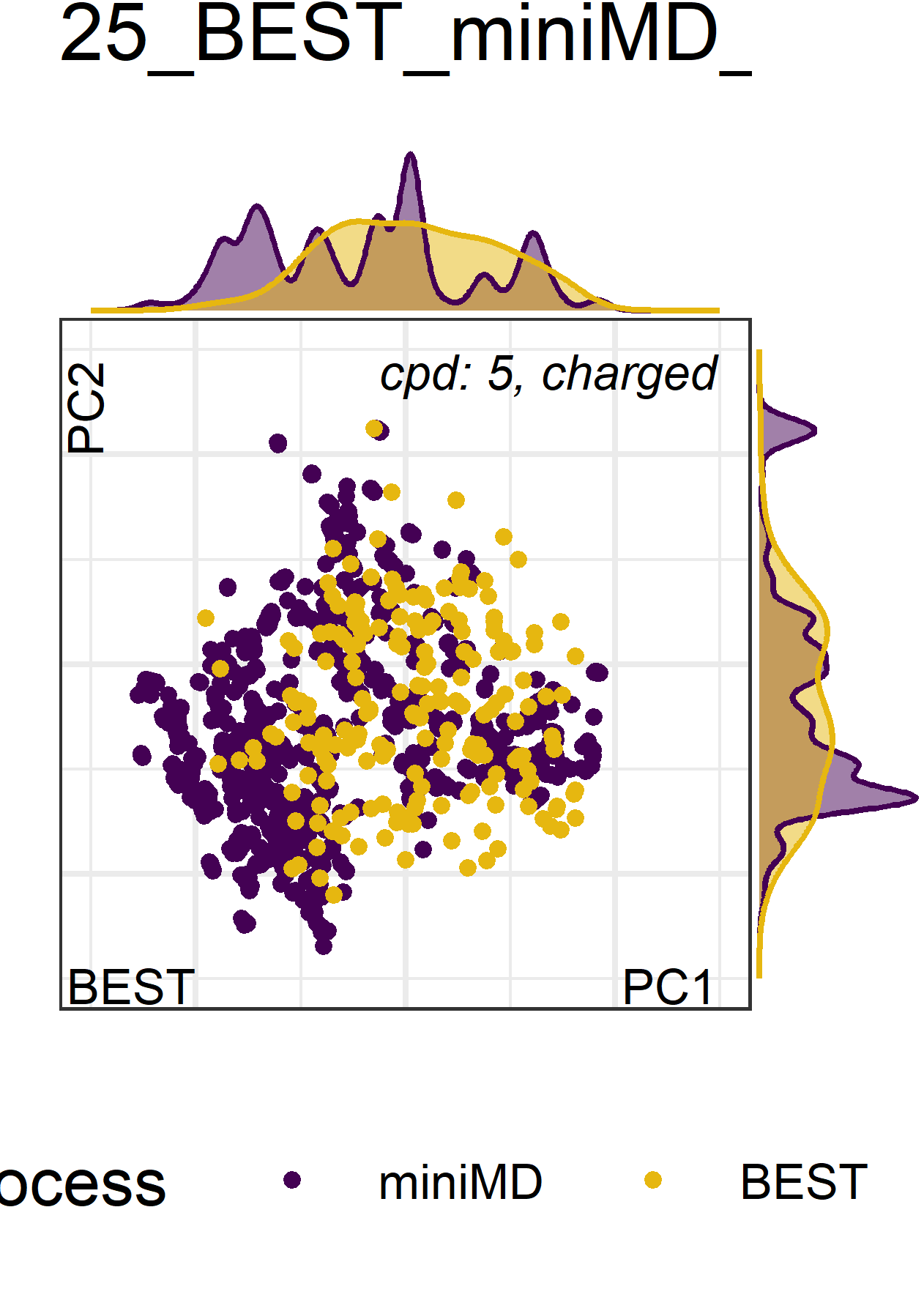

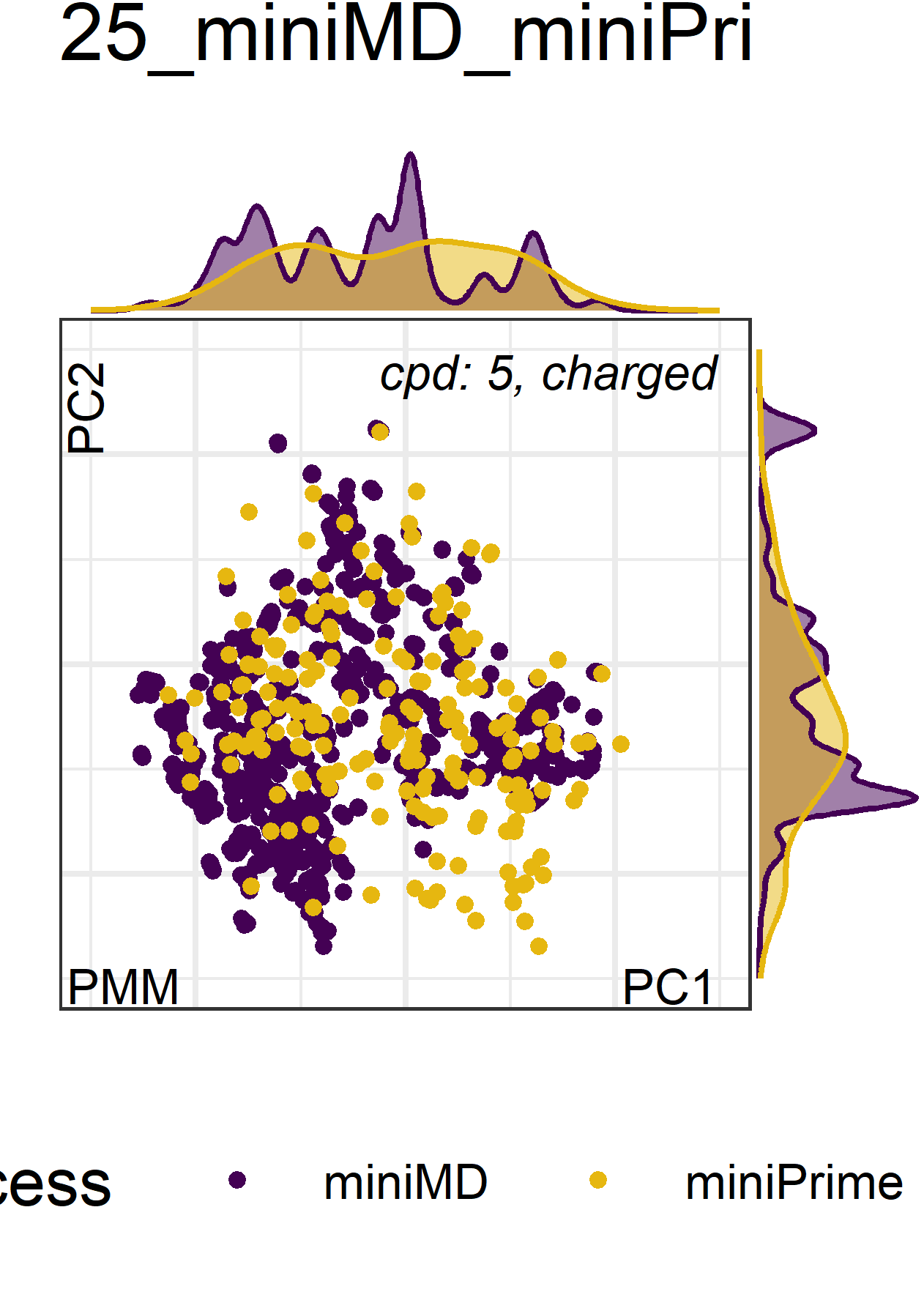

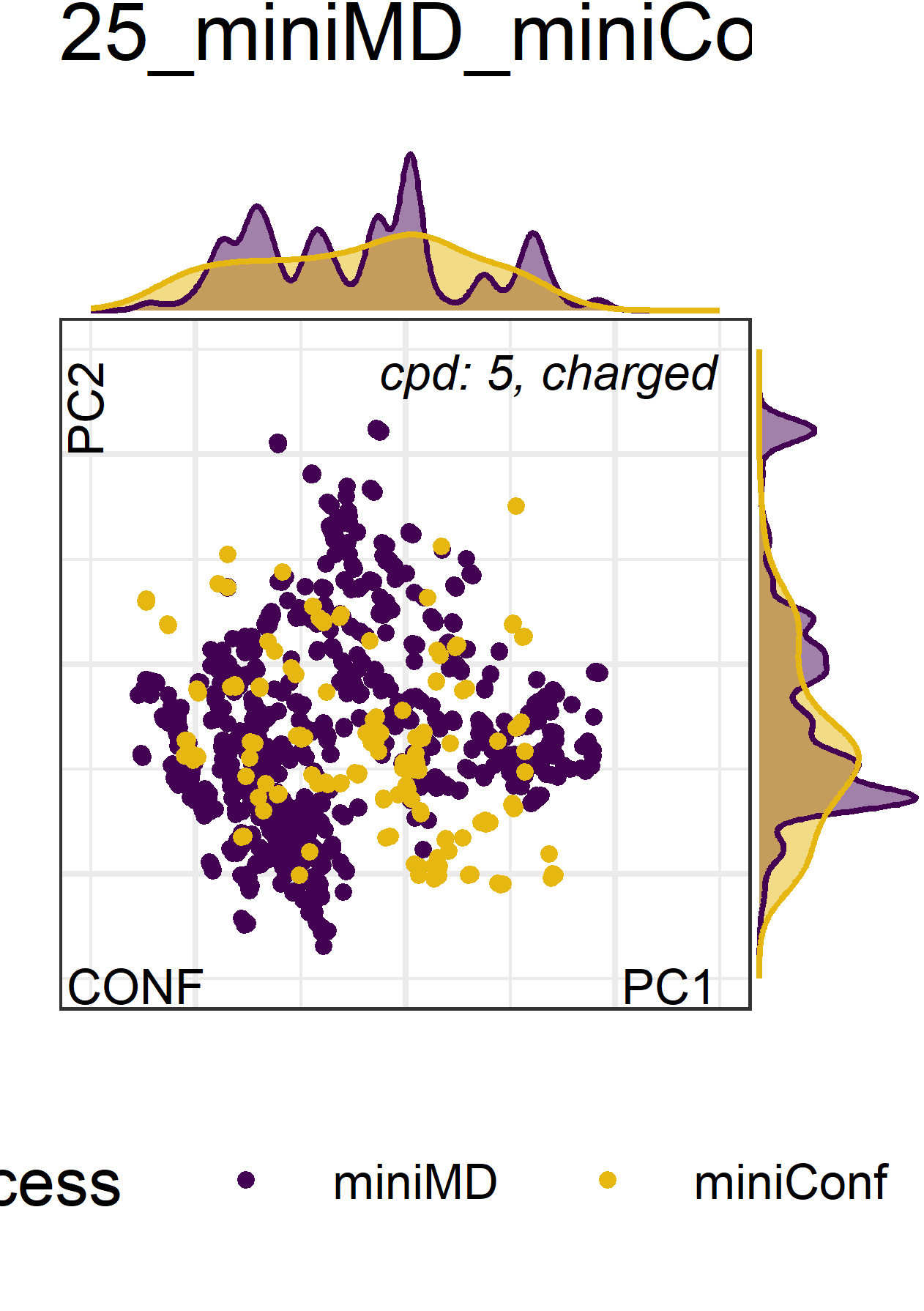


**6:**
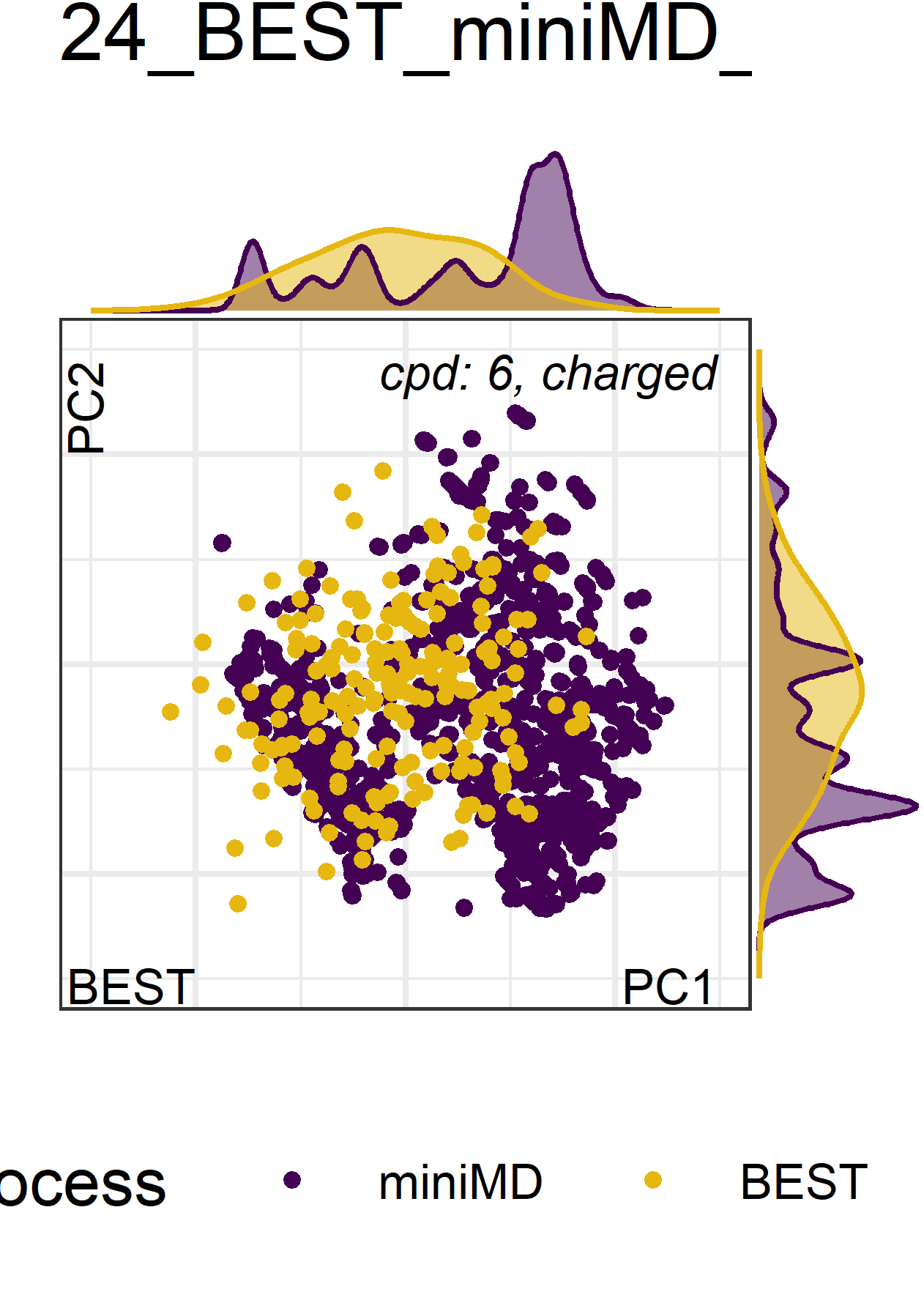

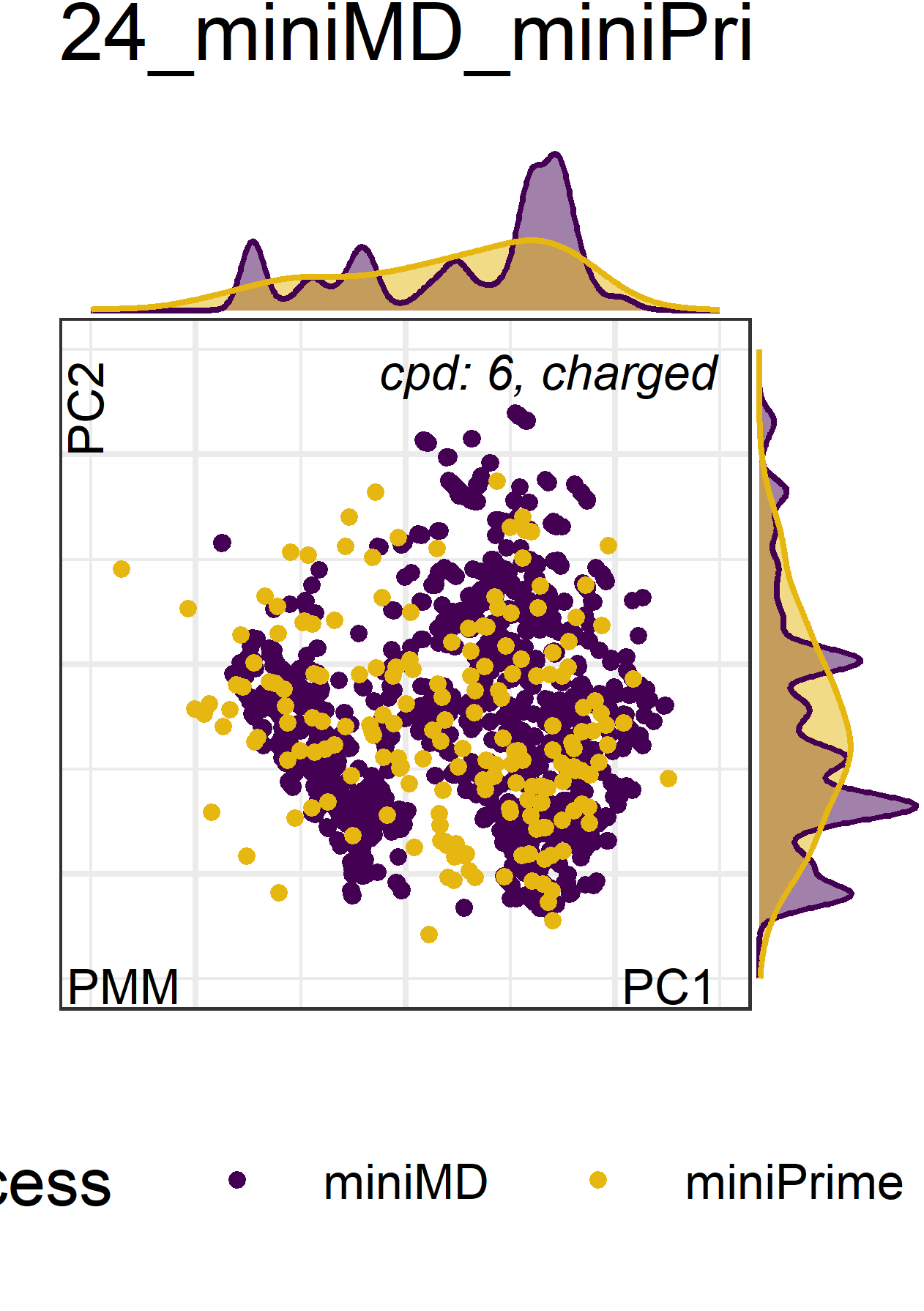

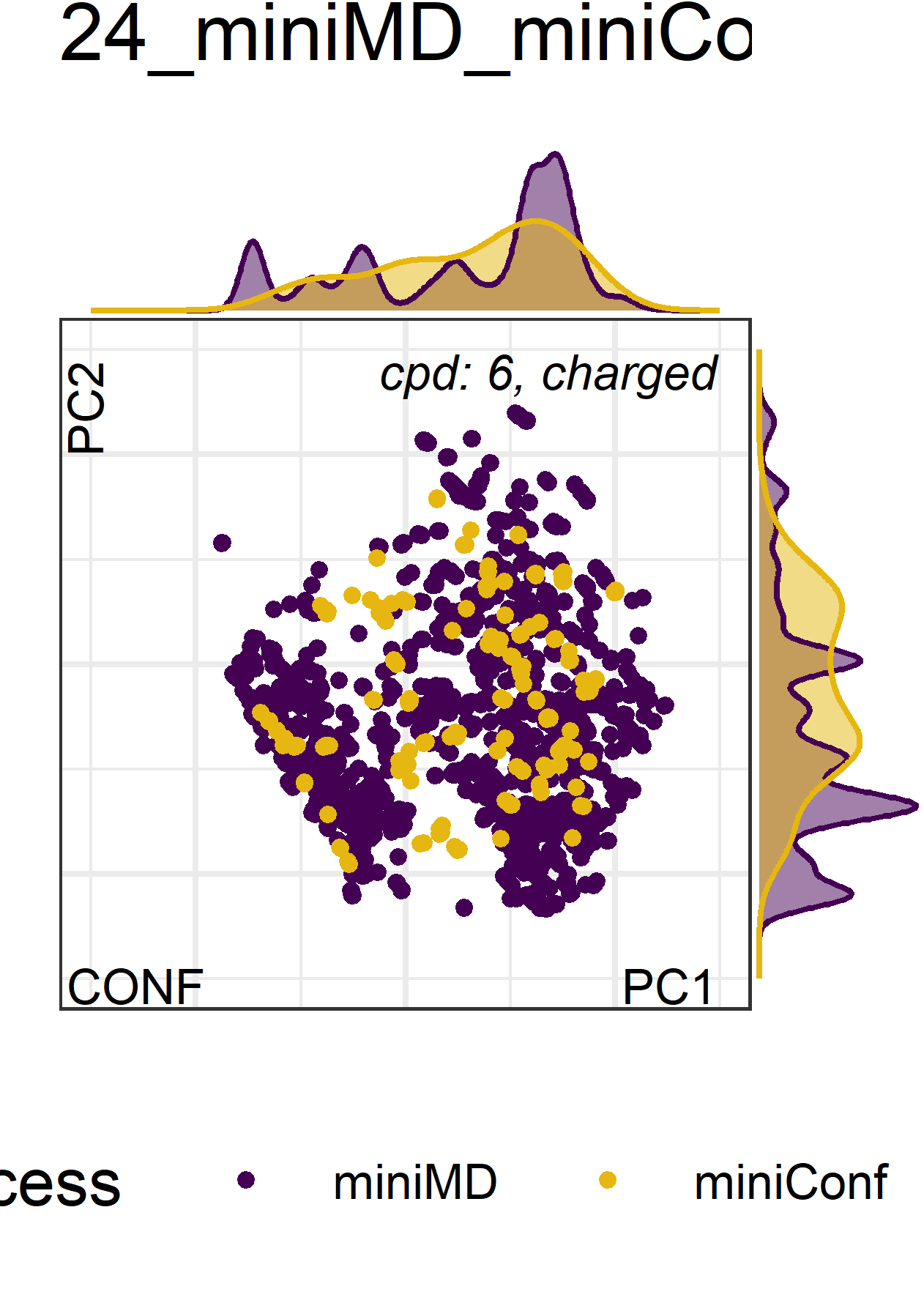
**7:**
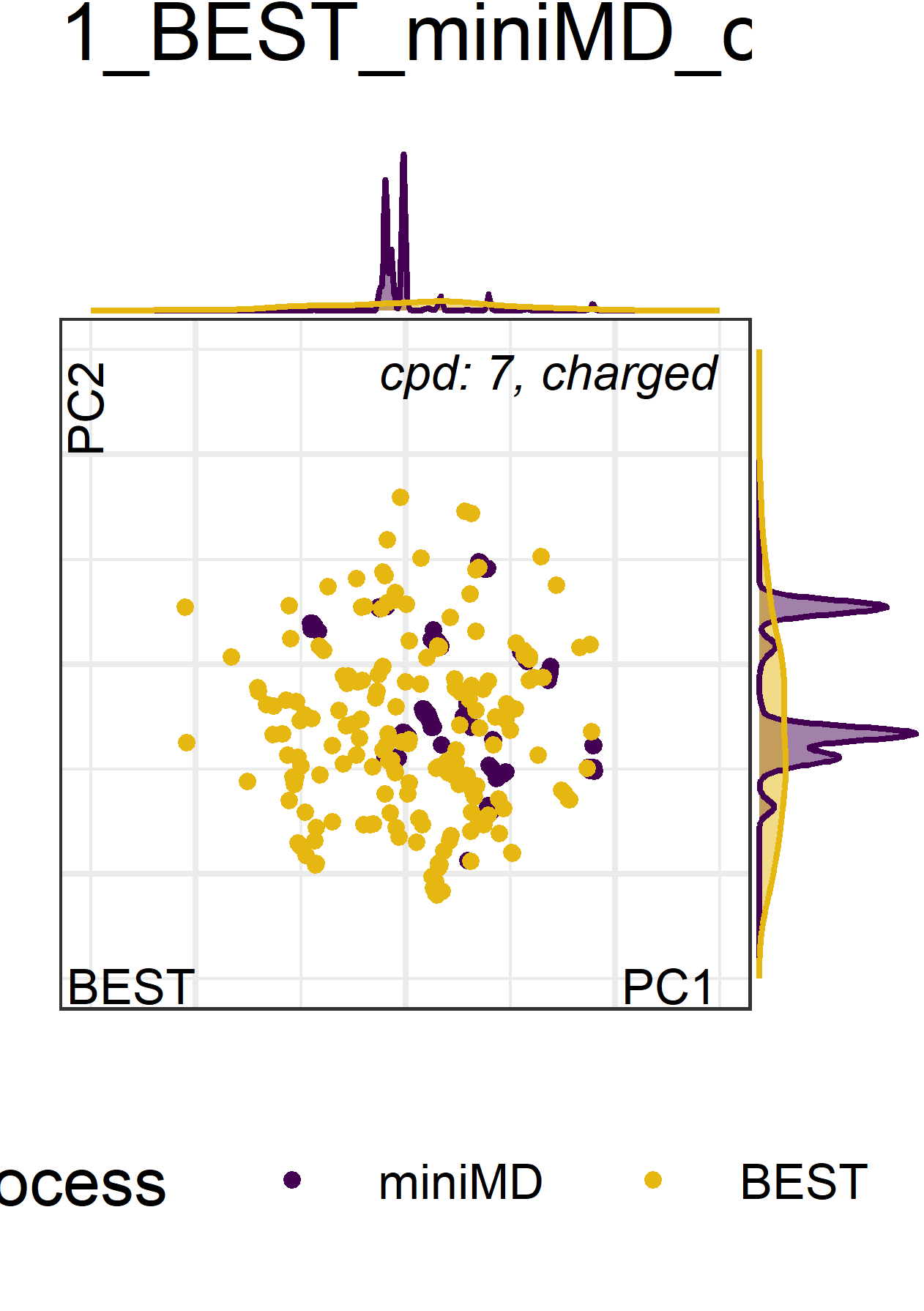

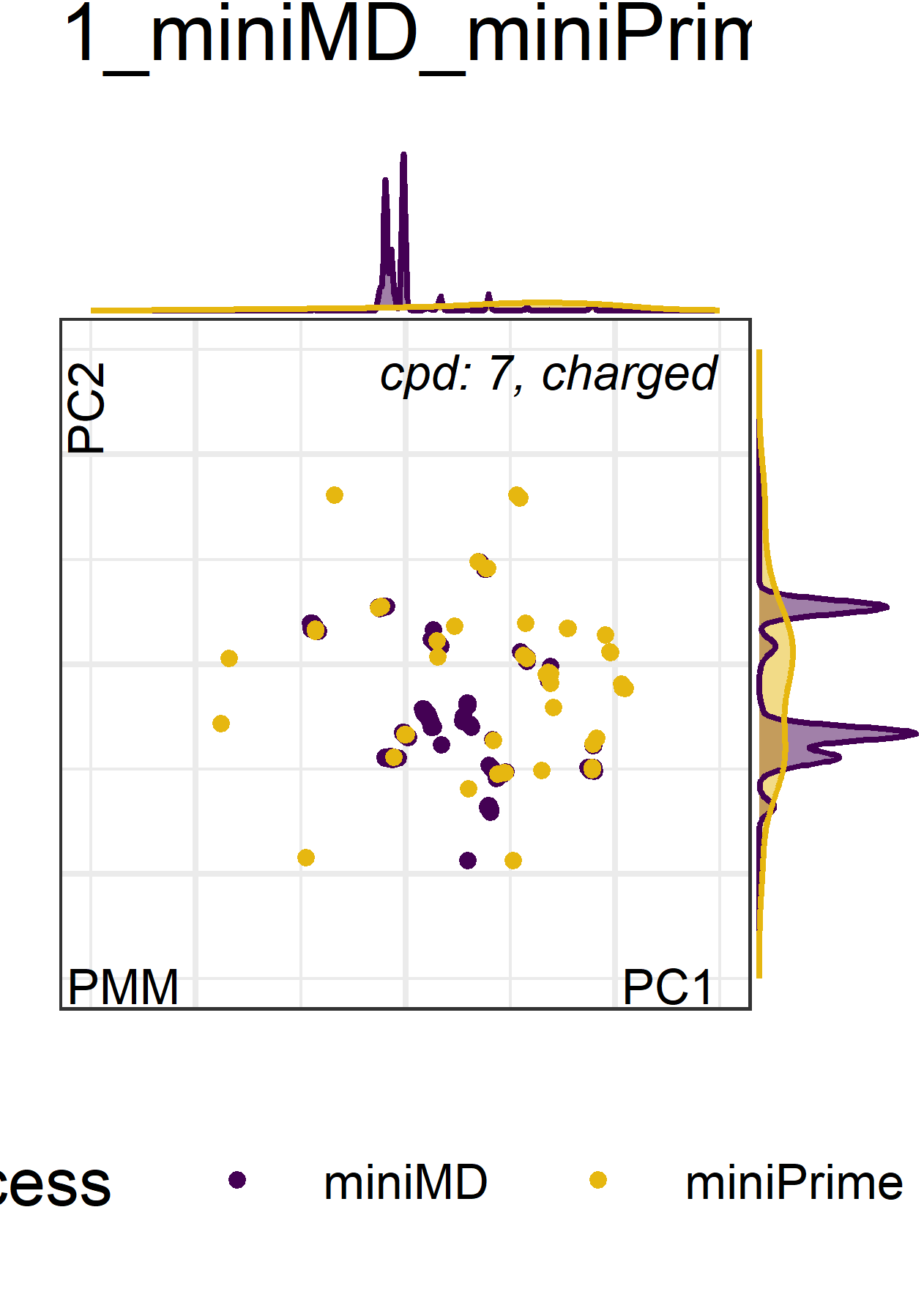

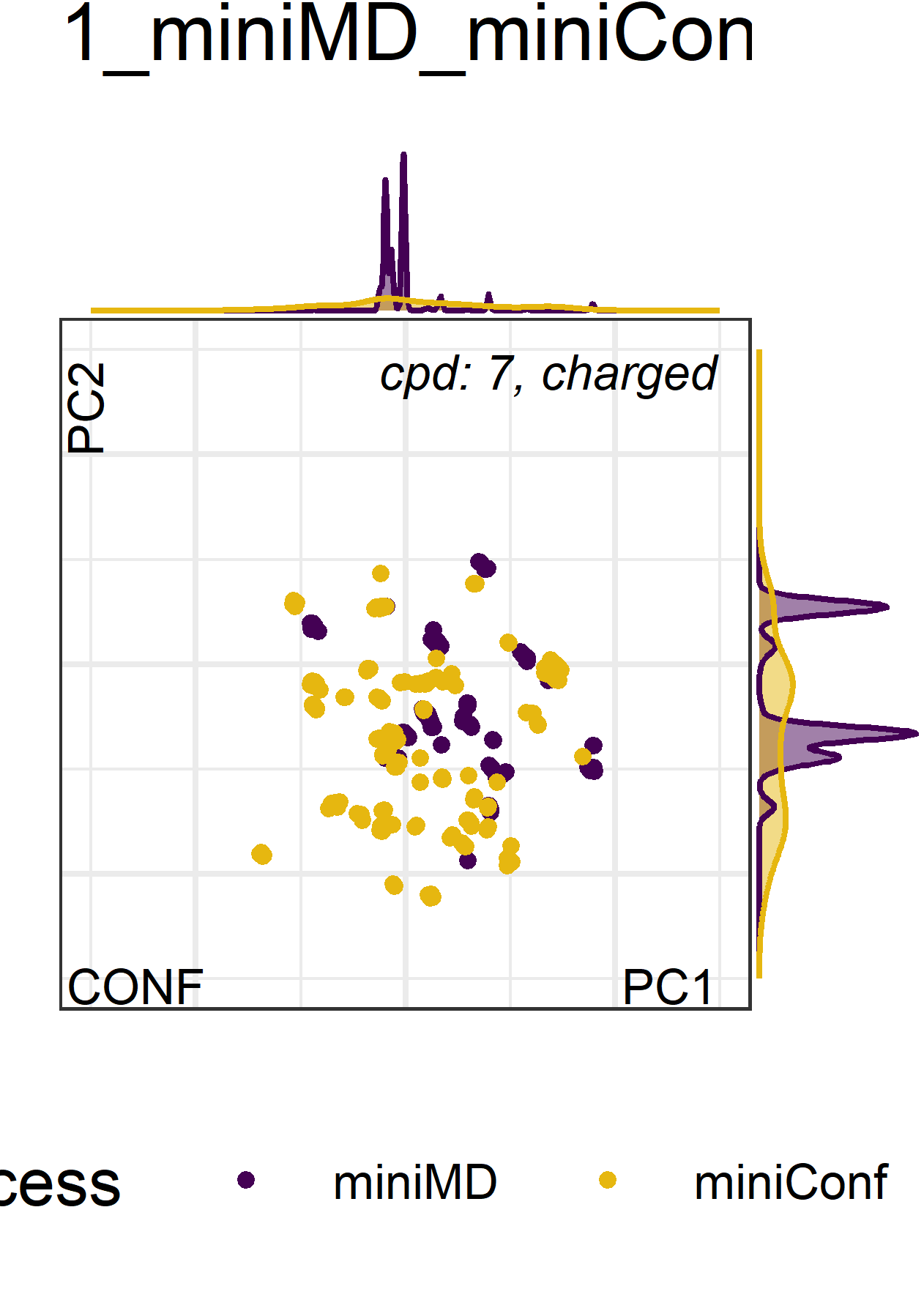


**Figure S10:** Maps of latent torsional space accessed by post-minimized MD snapshots (5 starting conformers, 300 K 100 ns each) for charged compounds **1** to **7** in SPC water in purple, overlaid with conformers created by BEST (left column), PMM (middle) and CONF (right) in orange. Note that compound **3** is missing since it is N-methylated.

**1:**
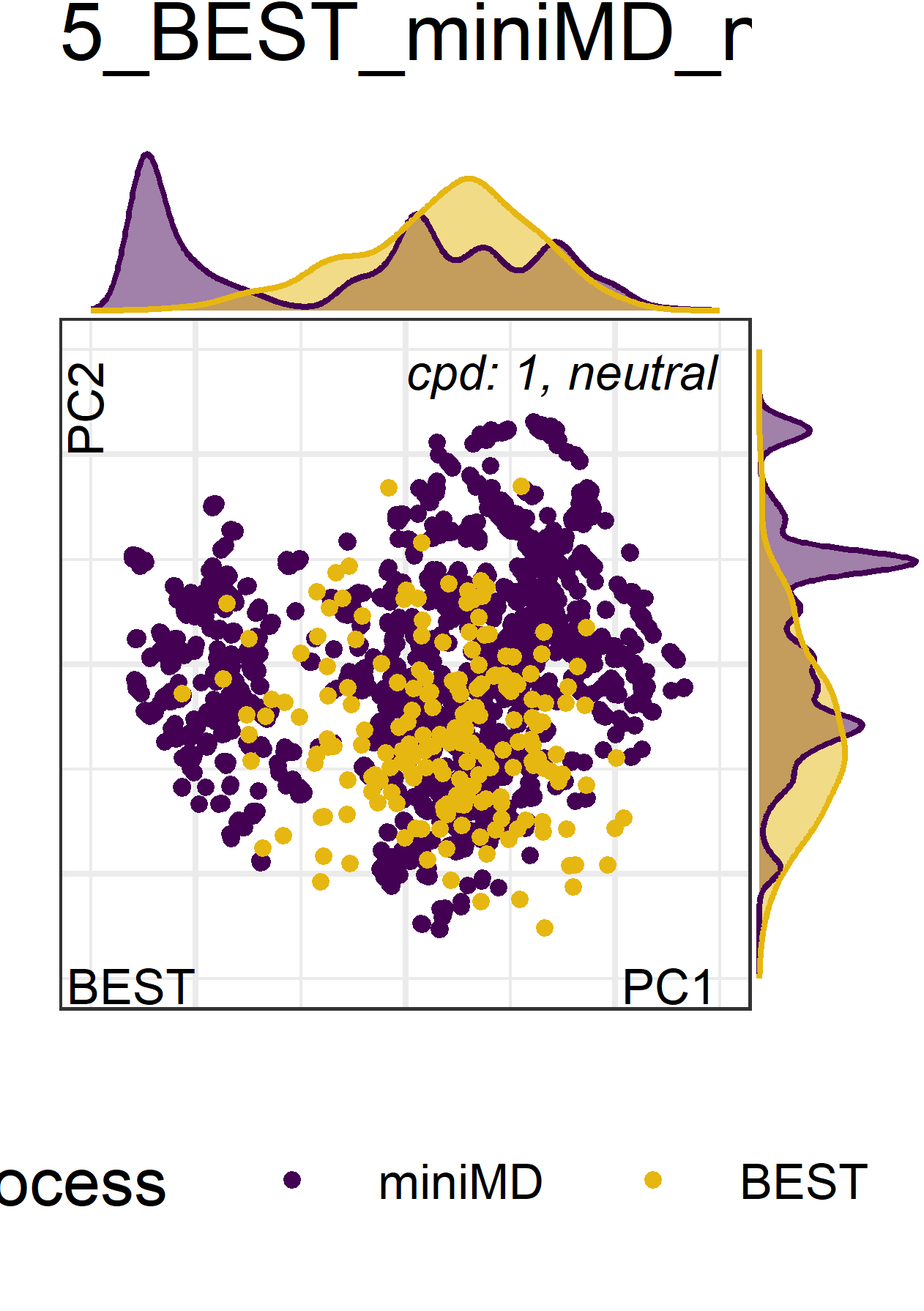

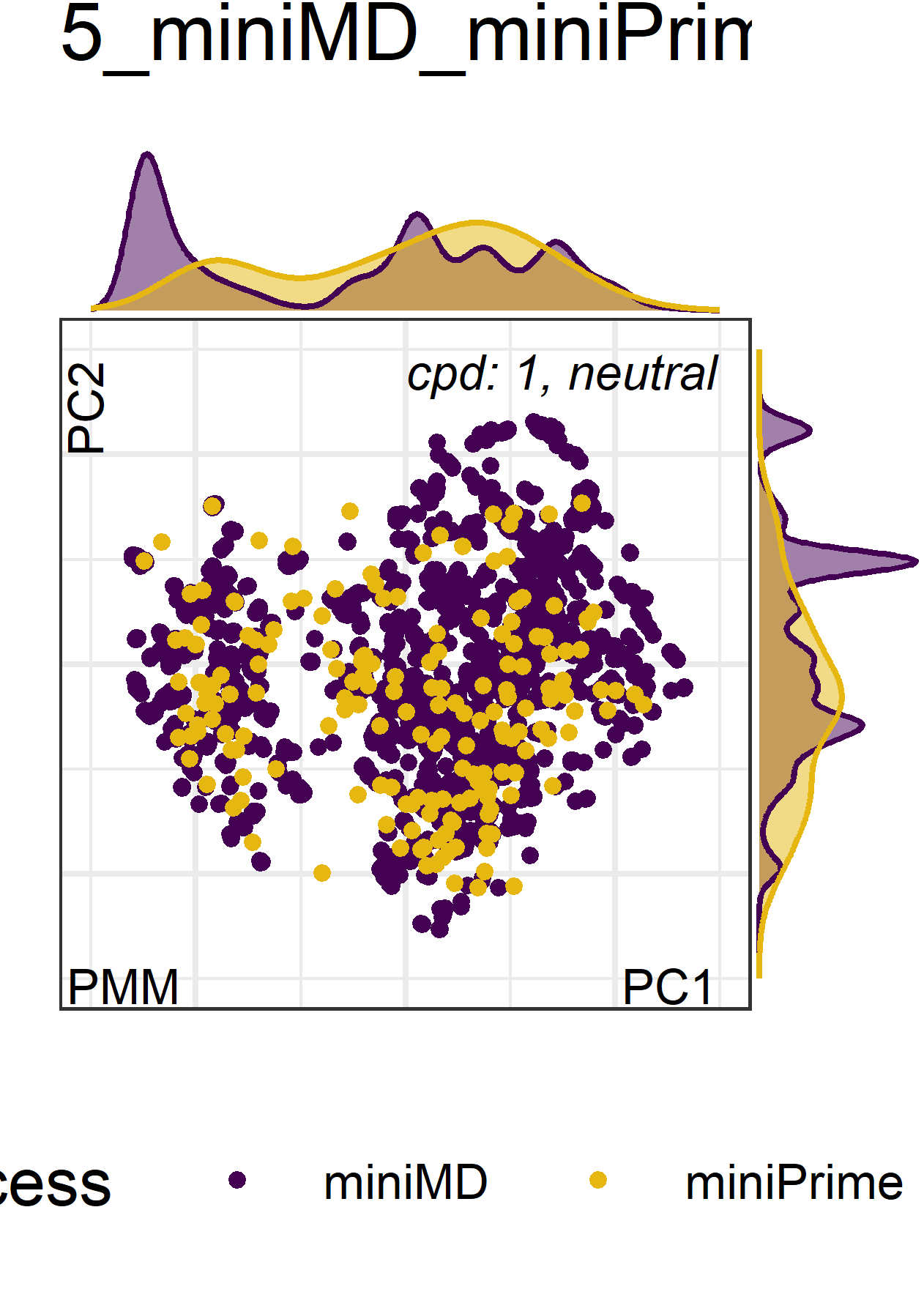

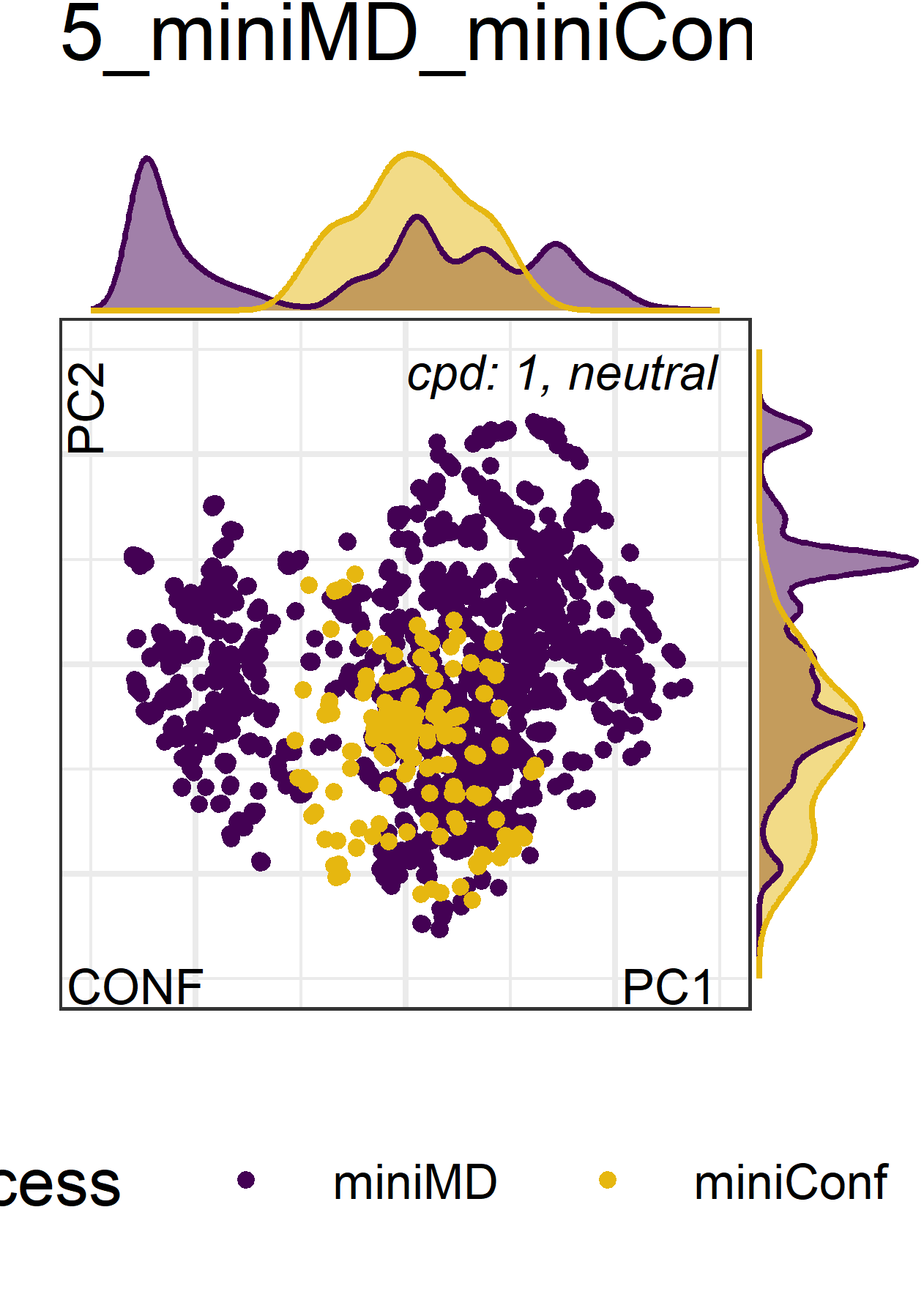
**2:**
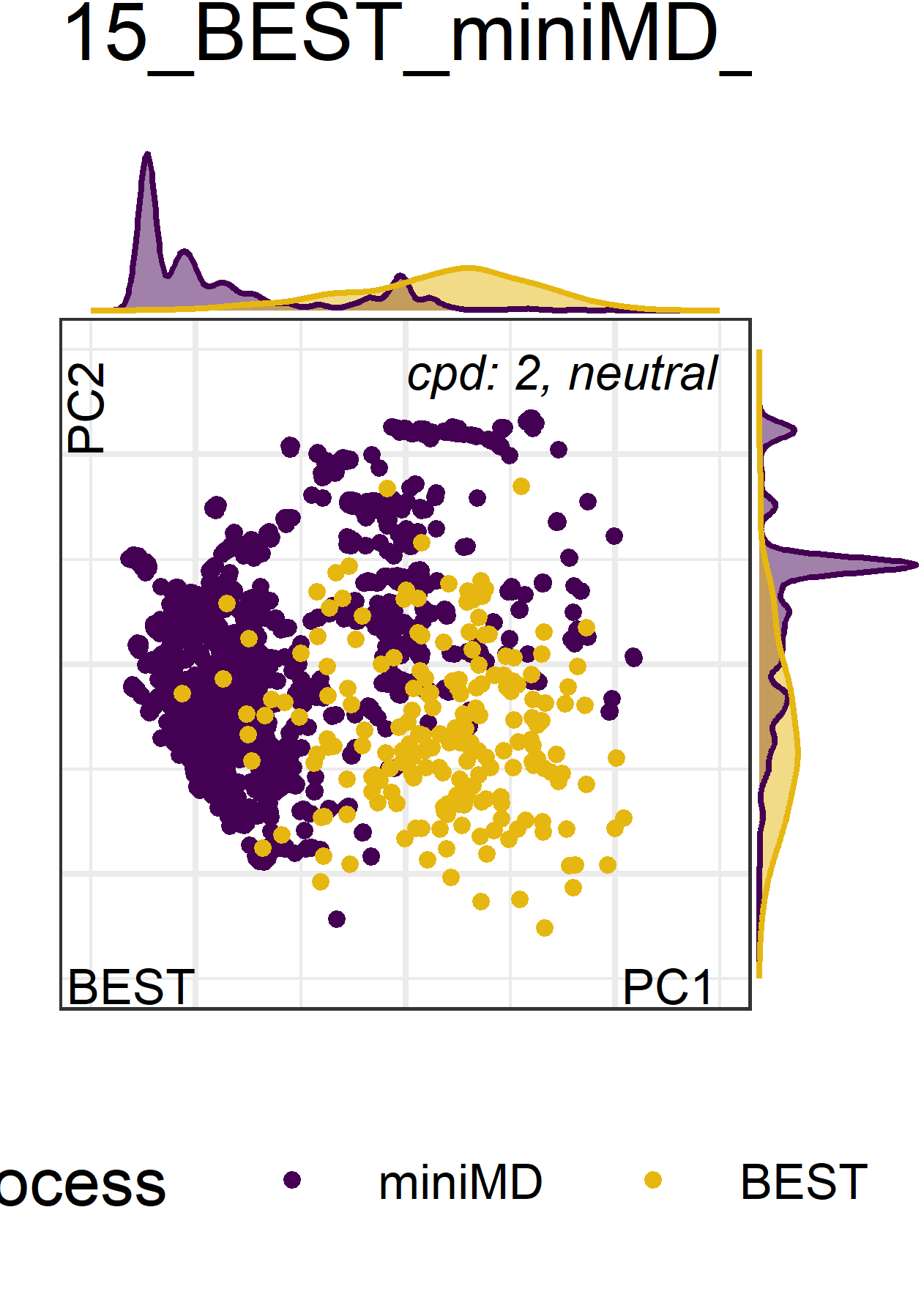
**3:** **4:****5:****6:****7:****Figure S11:** Maps of latent torsional space accessed by post-minimized MD snapshots (5 starting conformers, 300 K 100 ns each) for neutral compounds **1** to **7** in CHCl_3_ in purple, overlaid with conformers created by BEST (left column), PMM (middle) and CONF (right) in orange.

a)b)

c)d)

**Figure S12:** Maps of latent torsional space accessed by a) raw MD snapshots (5 starting conformers, 300 K 100 ns each, solvent water) for neutral compound **1**, b) raw (non-minimized) conformations by BEST, c) raw conformations by PMM, d) raw conformations by CON. Color-coding is by OPLS3e energy in implicit solvent water with color-code black (<6 kcal mol^-1^), grey (6 to 10 kcal mol^-1^), light grey above. The energy differences for CONF are defined by side chain orientations exclusively. Medium and high-energy conformations in d) therefore share the exact same ring atom dihedrals and are not visible in the plot.

**Figure S13:** 16 plots providing the complete conformer space mapping in 32 latent dimensions for **1**, neutral state in water. Projected are the minimised MD and the BEST generated conformers analogously to Figure 6. The numbers in brackets are the variance explained by the respective principal component. For PC 1 to 8 we observe no shift of the conformers.

a)

b)

**Figure S14** Dependence of total variance (trace) on the number of principal components considered; a) traces for minimized MD (blue) and BEST (green), both projected on the map created from all raw conformers, and the accumulated variance (cum. Proportion, red), showing that the curves cross at the point of about 90% of explained variance; b) traces for minimized MD (pink) and BEST (blue), with dashed lines for normal distributed noise (mean=0, sd=2*pi/180) generated for each sample for each dihedral angle. Noise effects both traces negatively.

**1**:

**2**:

**3**:

**4**:

**5**:

**6**:

**7**:

**Figure S15:** Histograms of 3D-PSA distributions for the MD snapshots (5 starting conformers, 300 K 100 ns each) of compounds 1 to 7; left column shows neutral compounds in water, center column charged compounds in water, right column neutral compounds in CHCl3. For N-methylated compound three there is no charged species.
